# MicroMetaSense: Coupling Plasmonic Metasurfaces with Fluorescence for Enhanced Detection of Microplastics in Real Samples

**DOI:** 10.1021/acssensors.4c02070

**Published:** 2024-12-27

**Authors:** Emre Ece, Yusuf Aslan, Nedim Hacıosmanoğlu, Fatih Inci

**Affiliations:** † UNAM-National Nanotechnology Research Center, Bilkent University, 06800 Ankara, Turkey; ‡ Institute of Materials Science and Nanotechnology, Bilkent University, 06800 Ankara, Turkey

**Keywords:** microplastics, nanoplastics, fluorescence microscopy, metal-enhanced fluorescence, plasmonic metasurfaces

## Abstract

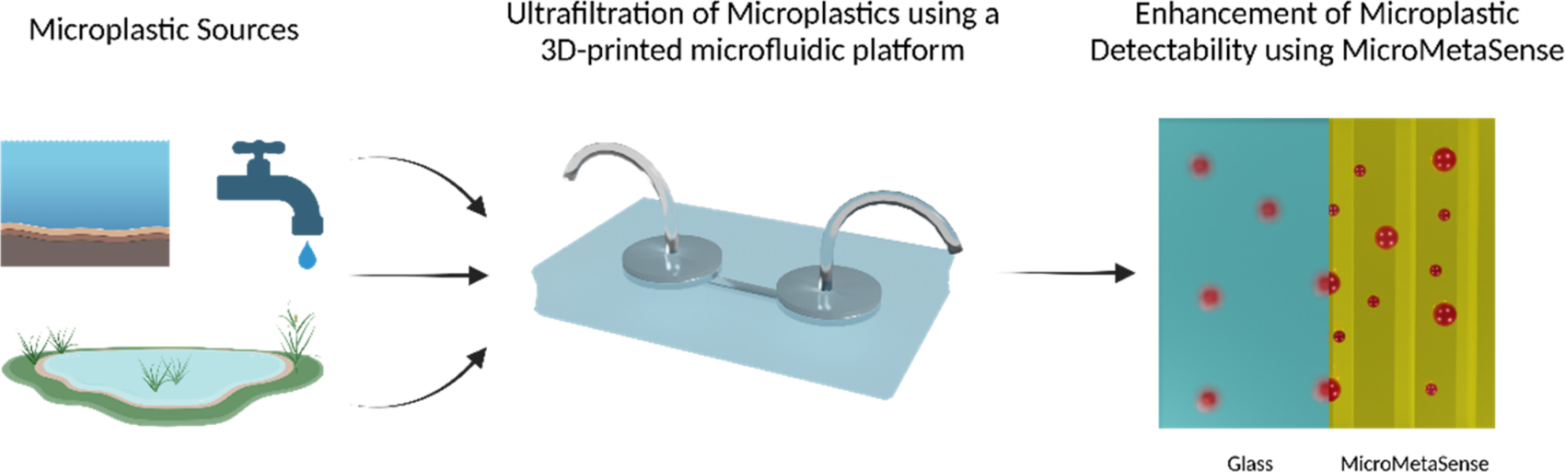

Diverse analytical techniques are employed to scrutinize microplastics (MPs)—pervasive at hazardous concentrations across diverse sources ranging from water reservoirs to consumable substances. The limitations inherent in existing methods, such as their diminished detection capacities, render them inadequate for analyzing MPs of diminutive dimensions (microplastics: 1–5 μm; nanoplastics: < 1 μm). Consequently, there is an imperative need to devise methodologies that afford improved sensitivity and lower detection limits for analyzing these pollutants. In this study, we introduce a holistic strategy, i.e., MicroMetaSense, reliant on a metal-enhanced fluorescence (MEF) phenomenon in detecting a myriad size and types of MPs (i.e., poly(methyl methacrylate) (PMMA) and poly(ethylene terephthalate) (PET)) down to 183–205 fg, as well as validated the system with real samples (tap and lake) and artificial ocean samples as a real-world scenario. To obtain precise size distribution in nanometer scale, MPs are initially processed with an ultrafiltration on-a-chip method, and subsequently, the MPs stained with Nile Red dye are subjected to meticulous analysis under a fluorescence microscope, utilizing both a conventional method (glass substrate) and the MicroMetaSense platform. Our approach employs a metasurface to augment fluorescence signals, leveraging the MEF phenomenon, and it demonstrates an enhancement rate of 36.56-fold in detecting MPs compared to the standardized protocols. This low-cost ($2), time-saving (under 30 min), and highly sensitive (183–205 femtogram) strategy presents a promising method for precise size distribution and notable improvements in detection efficacy not only for laboratory samples but also in real environmental samples; hence, signifying a pivotal advancement in conventional methodologies in MP detection.

Human beings have developed many products to advance life quality standards and shaped their lives according to these products such as polyethylene-based bags.^1^ Afterward, such plastics are encountered in clothes,^2^ food packaging products,^3^ and many other areas directly affecting our lives. In particular, microplastics (MPs) are formed when plastic wastes are below 5 mm in size, either through production processes or as a result of some breakup of large plastics.^4^ The most common polymeric forms of these hazardous impurities, classified as primary and secondary MPs, are poly(methyl methacrylate) (PMMA),^5^ poly(ethylene terephthalate) (PET),^6^ polyethylene (PE),^7^ polystyrene (PS),^8^ poly(vinyl chloride) (PVC),^9^ and polypropylene (PP).^7^ While fibers, pellets, sponges, erasers, and microbeads constitute the primary class of MPs,^10^ the secondary MPs are formed when large plastic wastes, including plastic bottles, plastic bags, tea bags, and fishing equipment, are broken into smaller pieces by external factors.^11,12^ External factors can be physical damage or UV radiation, and members of this class are also found in marine systems.^13,14^ Despite their significance in daily life, the devastating impact they cause to the environment and living organisms is inevitable.^11^ This detriment begins with the chemical processes of plastics^15^ and continues with the presence of these pollutants in food^16^ and living things.^17,18^ The hazard is expected to upsurge as the amount of plastic produced every year increases.^19^ For instance, annual plastic waste production reached approximately 400 million tons in 2023.^20^ Moreover, 0.5% of this plastic waste reaches the oceans.^21^

The fact that these small impurities spread to every point of the life cycle requires significant steps in purification and determination with high sensitivity and accuracy. For decades, many methods have aimed to sample, purify, and determine these contaminants with higher efficiency by considering savings in time and cost of analyses. In conventional methods, nets,^22^ sieving,^23^ and pump^24^ sampling methods are considered as the first step in the process of determining these impurities. After sampling, some extraction and separation methods (*e*.*g*., magnetic extraction,^25^ gel permeation chromatography (GPC),^26^ and ultrasonic methods^27^) are implemented to enhance the efficiency of MP analysis in complex environments containing many different wastes/products other than MPs. Microfluidic systems can also be implemented as an alternative technique for processing samples prior to analysis, preparing MPs for examination.^28,29^ In many studies, microfluidic systems are performed to isolate targeted nanosized samples from complex matrices^30^ and to isolate biological particles from different samples.^31^ Unlike size-based chromatography devices that are costly and time-consuming, these portable and affordable microfluidic systems are promising for collecting these impurities in a particular size distribution.^32−34^ Furthermore, the integration of filters with desired pore sizes into microfluidic systems also enables the collection of micro- or nanoplastics in a precise size range.^35^ On the other hand, in microscopic analyses, the contaminants such as sand grains similar in size to MPs and indiscernibly small MPs hold additional issues, making direct determination under the microscope challenging.^36^ For instance, as per an earlier report, sand grains and insect carapace are observed while analyzing MPs under an optical microscope.^37^

The sampled and processed MPs are identified and quantified using optical investigation, thermoanalytical, and spectroscopic methods.^38−40^ Among these, optical investigation is the simplest and most accessible method for MP quantification. However, it is prone to false-positive results.^41,42^ To address this limitation, scientists have turned to thermoanalytical and spectroscopic techniques. Pyrolysis-gas chromatography coupled with mass spectrometry (Py-GC/MS) is, for instance, a thermoanalytical method that employs the known pyrograms of pure polymers to identify thermally degraded MPs.^43,44^ Although thermoanalytical methods provide reliable results, the limitations of a minimum sample size of 100 μm, single-particle analysis per run, and the high cost of the equipment hinders their routine use for MP analysis.^40^ The most commonly used spectroscopic methods for identifying MPs are micro-Fourier transform infrared spectroscopy (micro-FTIR), micro-Raman spectroscopy, and laser direct infrared imaging (LDIR).^45−47^ Micro-FTIR spectroscopy determines the chemical fingerprints of MPs through the analysis of molecular vibrations. The operation of this method must be optimized in transmission or reflection modes to ensure sufficient absorption of interpretable spectra, as each mode is suitable for specific particle sizes and thicknesses.^46^ However, this method is time- and labor-intensive since hundreds or thousands of MPs must be measured individually.^40,46^ Moreover, the diffraction limit of infrared spectroscopy is physically restricted to 10 μm,^48^ which hinders the investigation of submicron MPs (100 nm to <1 μm). A relatively new variation of micro-FTIR is LDIR, where the sample acquisition time is reduced using quantum cascade lasers (QCLs) and focal plane arrays (FPAs).^46,49,50^ However, the high cost associated with FPAs and QCLs raises questions about the feasibility of this method for routine MP analysis. The last spectral technique is micro-Raman spectroscopy, which offers relatively faster MP analysis with automation and improved spatial resolution (100 nm to 1 μm)^46,51^ by using the vibrational fingerprints of MPs obtained from inelastic light scattering. Despite the improved scanning speed compared to micro-FTIR, this method still requires significant measurement time, and the laser can cause fluorescence interference in MPs.^46,52^ While these spectroscopic techniques are reliable in MP identification and detection, they are time-consuming and require specialized equipment. Considering the growing impact of MPs on the environment^53^ and medicine,^54^ a much accessible and facile method is required for the identification of MPs. To address this challenge, MPs can be stained with fluorescent dyes and examined under a standard fluorescence microscope or UV light photobox as a screening strategy prior to more sophisticated techniques, potentially reducing the assay cost and providing rapid approach for MP analysis.^55−60^ Research on this subject has demonstrated progress on decreasing leach outs^61^ and nonselective staining.^62^ For this purpose, specific staining of MPs with Nile Red (9-(diethylamino)-5H-benzo[*a*]phenoxazin-5-one) not only enhances their identifiability under the fluorescence microscope but also eliminates many minerals that affect the analysis, as the Nile Red dye is ineffective in staining them.^63^ However, in this scenario, poor sensitivity and impediments in detecting the small size of MPs remain some compelling challenges. Any advancements in fluorescence measurements represent a significant step forward in identifying smaller micro- or nanoplastics. These smaller particles typically emit lower fluorescence intensity due to their reduced surface area compared with larger MPs. However, there is still a need to increase the fluorescence intensity for such cases. The challenge of low fluorescence intensity of MPs can be surpassed by the integration of plasmonic substrates.

The coupling between fluorescence and metallic and/or plasmonic substrates can induce a phenomenon called metal-enhanced fluorescence (MEF) or plasmon-enhanced fluorescence (PEF), which increases the fluorescence emission intensity by several orders of magnitude.^64^ This enhancement is attributed to the formation of surface-plasmon polaritons (SPPs) on the metal–dielectric interface upon incoming radiation of plasmonic resonance, which is also designed to overlap with the excitation and/or emission spectrum of the nearby fluorophore. The surface plasmons form electric hot spots that increase the excitation rate of the fluorophore, which also increases the spontaneous emission rate. This results in enhanced fluorescence intensity and provides a better evaluation of weakly emitting minute concentration samples. The geometry and materials selection (permittivity) of plasmonic structures contribute to the design of electric hot spots formation and density.^65^ Therefore, metasurfaces, two-dimensional and subwavelength periodic counter structures of metamaterials,^66^ are widely used to utilize MEF. Recent reports on metasurfaces present precise control of light–matter interactions at subwavelength scales that can be further tuned to couple either fluorescence excitation or emission spectra.^67−69^ As an example, nanodimensional metal structures are preferred for MEF with improved excitation fields or modification of the radiative and nonradiative rates of fluorophores through the metal structure.^70−73^ In particular, the utilization of optical metasurfaces as a source for MEF is advantageous due to their ability to spatially confine the electric-field and fine-tune the field intensity through precise control of the periodic design and the selection of coating materials.^74,75^ Yet, metasurfaces-aided MEF applications typically require expensive fabrication techniques, such as laser interference lithography (LIL),^76^ focused ion beam (FIB),^69^ electron-beam lithography (EBL),^68^ and metasurfaces usually require expensive fabrication methods, such as particle-beam lithography (PBL),^77^ which significantly increase substrate costs and limit their scalability in MEF applications, which restrict their scalable utilization in MEF applications. Considerable efforts have been made to develop cost-effective metasurface-aided MEF substrates, utilizing methods like colloidal lithography (CL)^78^ and nanoimprint lithography (NIL).^79^ These advances have the potential to enable the use of a metasurface-enhanced MEF in on-site detection of MPs.

To the best of our knowledge, the integration of plasmonics-aided MEF for MP detection has only been demonstrated once, with Wei et al. successfully applying nanopillar structures to enhance intrinsic fluorescence of MPs.^63^ However, fabricating such structures requires multistep and expensive fabrication processes such as reactive ion etching (RIE) using SF_6_ (sulfur hexafluoride), oxygen plasma, and following beam evaporation of metallic layers.^80^ Minimizing the number of process steps and devices used in a clean room will aid the expansion and utility of the MP detection field. Unlike nanopillar-based systems, our metasurface substrate, MicroMetaSense, requires no nanopatterning and is fabricated through a single thermal evaporation process. This significantly reduces the number of clean-room fabrication steps and avoids the usage of costly, high-precision techniques, such as EBL^68^ and FIB milling.^67,69,71^ Our platform offers a facile (four steps: filtration, staining, sampling to the MicroMetaSense, and analyzing), cost-effective ($2), and practical solution for applying MEF, integrating seamlessly into the preliminary detection of MPs using fluorescence microscopy. This simplicity would facilitate widespread detection of MPs in environmental samples. Based on the available literature, no other study has achieved such a cost-effective deployment of MP detection through a metasurface-aided MEF strategy with environmental samples, reducing the experiment duration to less than 30 min and achieving to detect 183–205 fg of MPs.

In this study, for MP detection and examination, we present a holistic approach (i.e., MicroMetaSense) that combines plasmonic metasurfaces with fluorescently stained MPs (Figure 1). In this setting, plasmonic metasurfaces act as the MEF source since the electric-field can be spatially confined. Once coupled with fluorescence spectra (either excitation or emission), the intensity is tuned through their periodic design and coating materials (titanium, silver, and gold). They also increase the radiative decay rate of fluorescence signals derived from the Nile Red-stained MPs. In fabricating metasurfaces, we repurpose plastic optical disks with intrinsic nanograting structures that can be easily fine-tuned after a facile chemical etching step, thereby not only reducing the cost ($1) but also providing an alternative to applicability and sustainability to the methods used in MP detection studies. Prior to analyses, we process MP samples with an ultrafiltration microfluidic chip, and hence, undesired large wastes (*e*.*g*., fiber, mud, and moss) in the source are eliminated. MPs are prepared as spiked samples (*i*.*e*., PMMA and PET) and real samples from tap water, lake water, and artificial ocean water. Once they are applied on the MicroMetaSense, we calculate the signal enhancements and limit-of-detection (LOD) for spiked samples (PMMA spiked in water and PET spiked in water) and real samples (tap water, lake samples, and artificial ocean water), and compare these results to the ones derived from the standard protocol using a glass surface. Overall, we here present a promising strategy for MP detection that reduces the assay cost and duration, and at the same time, improves the sensitivity down to femtogram levels. For detecting MP contamination with minimal effort, the expansion and utility of such systems are essential to take a step forward in the routine analysis of environmental samples.

**Figure 1:**
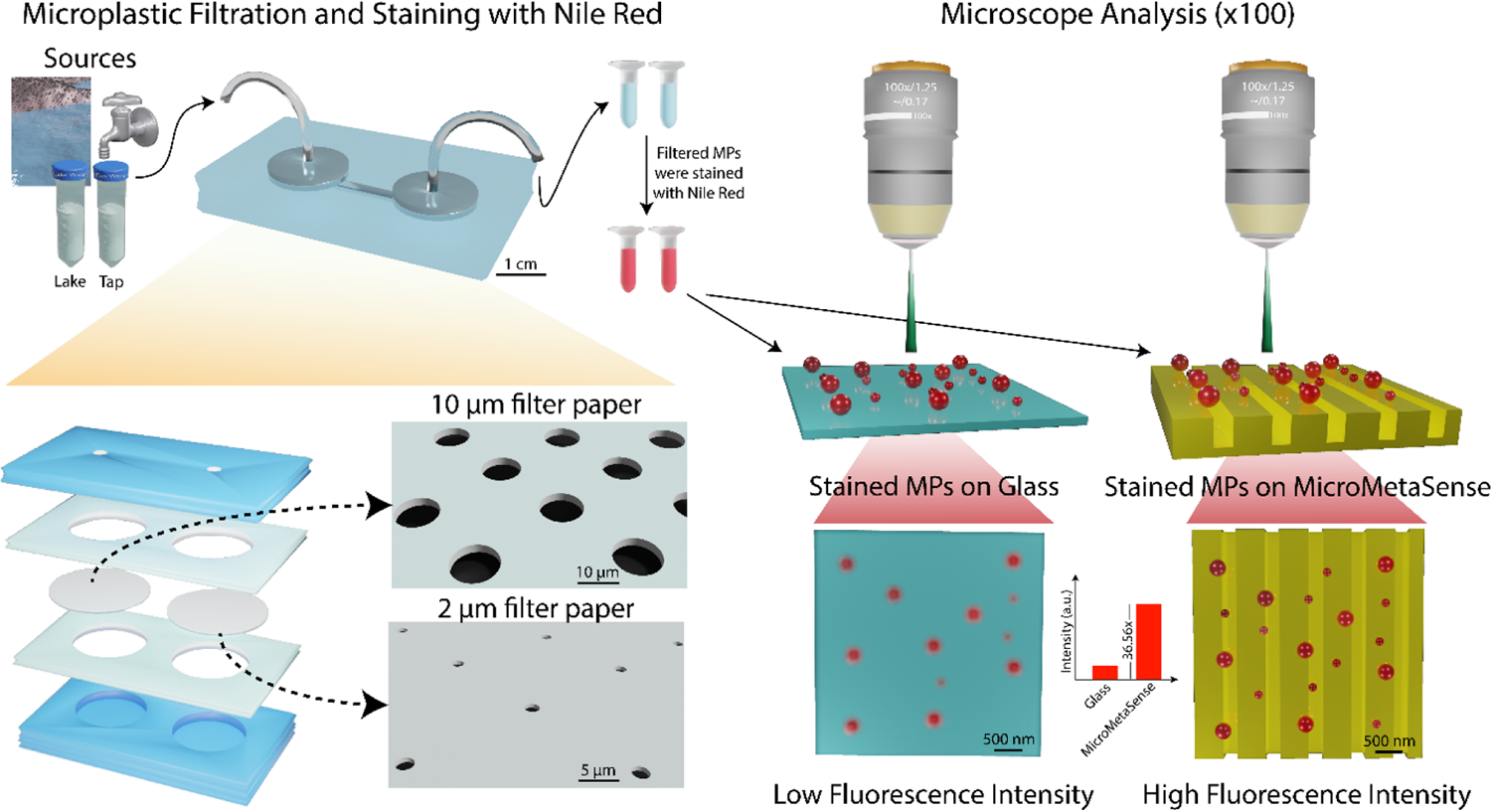
Workflow includes the ultrafiltration of MPs on a microfluidic chip, and subsequently analyses of Nile Red-stained MPs on the MicroMetaSense platform by demonstrating their dimensions with representative scale bars. As a conventional system, the results derived from a glass substrate are compared with the ones from our platform.

## Results and Discussion

### Ultrafiltration Chip

**Figure 2:**
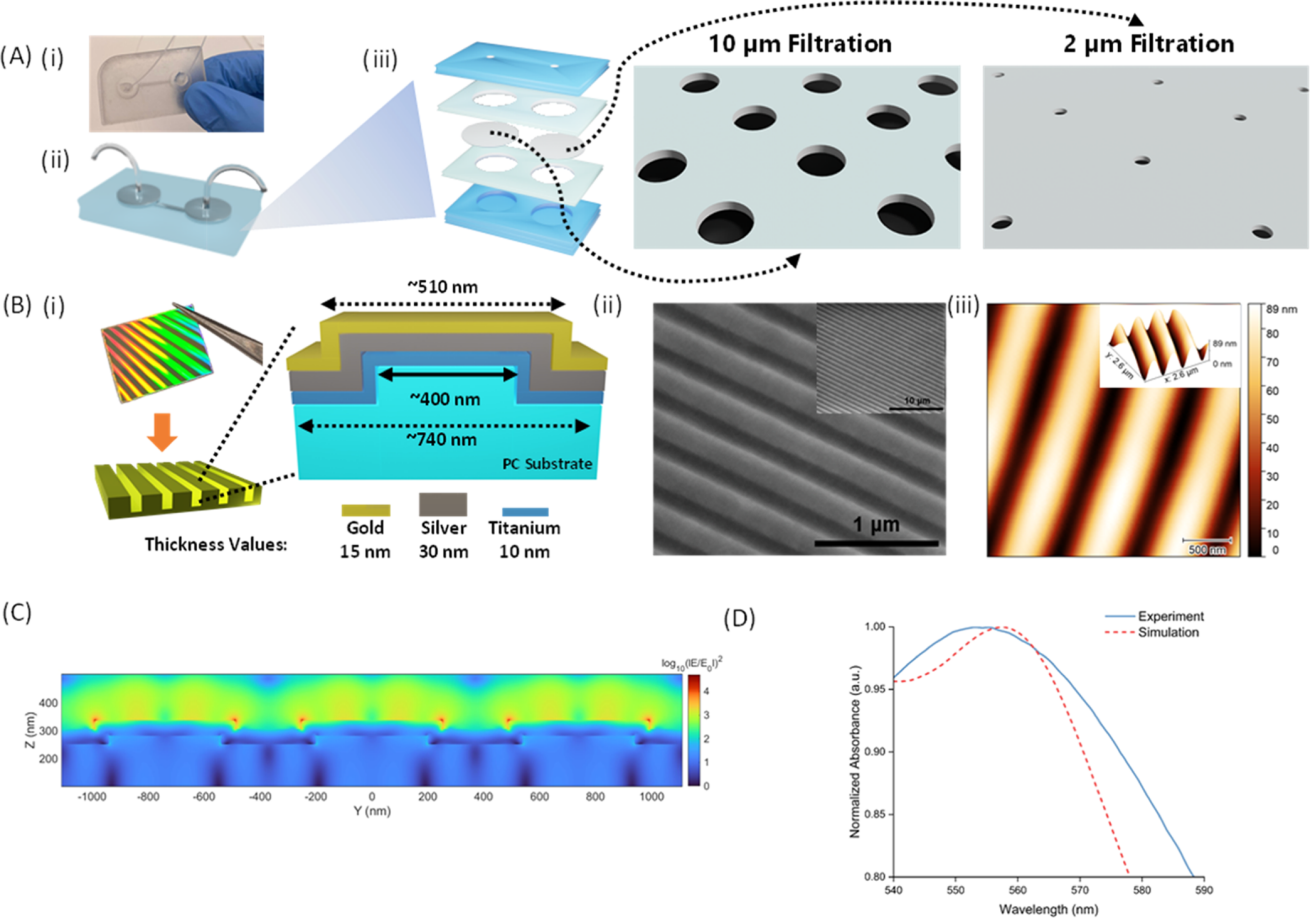
(A) (i) A photograph of the ultrafiltration chip is shown. (ii) On the right side of the photograph, a schematic for the chip components is presented, depicting different filter sizes on both the inlet and outlet ports (iii). (B) (i) MicroMetaSense consists of polycarbonate (PC) substrates and layer-by-layer metal coatings, i.e., titanium (10 nm), silver (30 nm), and gold (15 nm). The surface of MicroMetaSense features continuous and periodic grating structures with an approximate periodicity of ∼740 nm and spacing of ∼450 nm without metal coatings and ∼510 nm with metal coatings. The topography of the MicroMetaSense is demonstrated by SEM (ii) and AFM (iii). (C) Electric-field distribution of MicroMetaSense is numerically analyzed using the FDTD method. (D) Normalized absorption spectrum of the simulated MicroMetaSense demonstrates results consistent with the experimental data.

The filtration process for PET, tap, and lake sources was completed to obtain more precise size distribution using a microfluidic-based ultrafiltration platform (Figure 2A-i,ii), consisting of cellulose-based filtration papers with pore widths of 10 and 2 μm (Figure 2A-iii). In order to start the filtration process, samples were injected into the preformed chip with a syringe filter with 40 μL/min flow rate, and steady flowthrough was ensured by checking the possible leakage during the process. For all samples, microchip flowthrough was collected in a 2 mL of volume for future use and analysis.

### MicroMetaSense Characterization

Off-the-shelf digital versatile disks (DVDs) were utilized as plastic-templated substrates for clean-room-free fabrication of MicroMetaSense (Figure 2B-i). The fabrication process was performed following the methods in a previous study.^81^ The process initiated with the removal of contaminants and a photoresist layer from the optical disks. Next, the built-in grating structure on the disk surface was chemically etched. Later, the etched surface was coated with titanium (Ti, 10 nm), silver (Ag, 30 nm) and gold (Au, 15 nm) using thermal evaporation (Figure 2B-ii). This resulted in a uniformly periodic and multilayered grating structure (Figure 2B-iii and iv) of multilayer metallic films. The surface roughness analysis of the MicroMetaSense also indicates a uniform periodicity, which plays a significant role in the formation of a consistent electric-field necessary for MEF analysis of MPs (Supporting Information Figure 1). The periodic multilayers excite and form plasmonic resonance due to the coupling between waveguides and surface-plasmon polaritons (SPPs). In addition, this plasmonic response was numerically studied using the finite-difference time-domain (FDTD) method and compared with experimental results. The numerical analysis of the metasurface revealed near-field enhancement at the edges of the grating structure (Figure 2C). This near-field enhancement is able to yield metal-enhanced fluorescence when a fluorophore with a suitable excitation/emission spectrum and appropriate fluorophore–metasurface distance is present.^82^ Further, the analysis of the absorption spectrum of the MicroMetaSense revealed a plasmonic resonance between 550 and 560 nm (Figure 2D). This resonance can enhance fluorescence when coupled with the excitation spectrum of red-emitting fluorophores, such as Nile Red.^83^

### Characterization of MPs

The initial step involved characterizing functional groups using attenuated total reflection-Fourier transform infrared spectroscopy (ATR-FTIR), followed by thermogravimetric analysis (TGA) to ascertain the degradation temperatures of MPs. Subsequently, MPs were stained with Nile Red at 80 °C for 15 min before undergoing fluorescence microscopy. TGA was employed to determine the degradation temperatures and assess potential polymeric structure damage at the specified incubation temperature (Supporting Information Figure 2). ATR-FTIR analysis was specifically performed on commercial products of PMMA and PET among the four different MP sources. PMMA-based MPs were readily available as a commercial product, while PET MPs were produced in-house using a homogenizer. ATR-FTIR was not conducted on tap water and lake samples due to the presence of diverse MPs. Furthermore, the size distribution of MPs (except PMMA) was insufficiently precise for fluorescence microscopy. Consequently, PET spiked in water, tap water, and lake samples were sequentially filtered using an ultrafiltration microfluidic chip to achieve a more precise size distribution.

**Figure 3:**
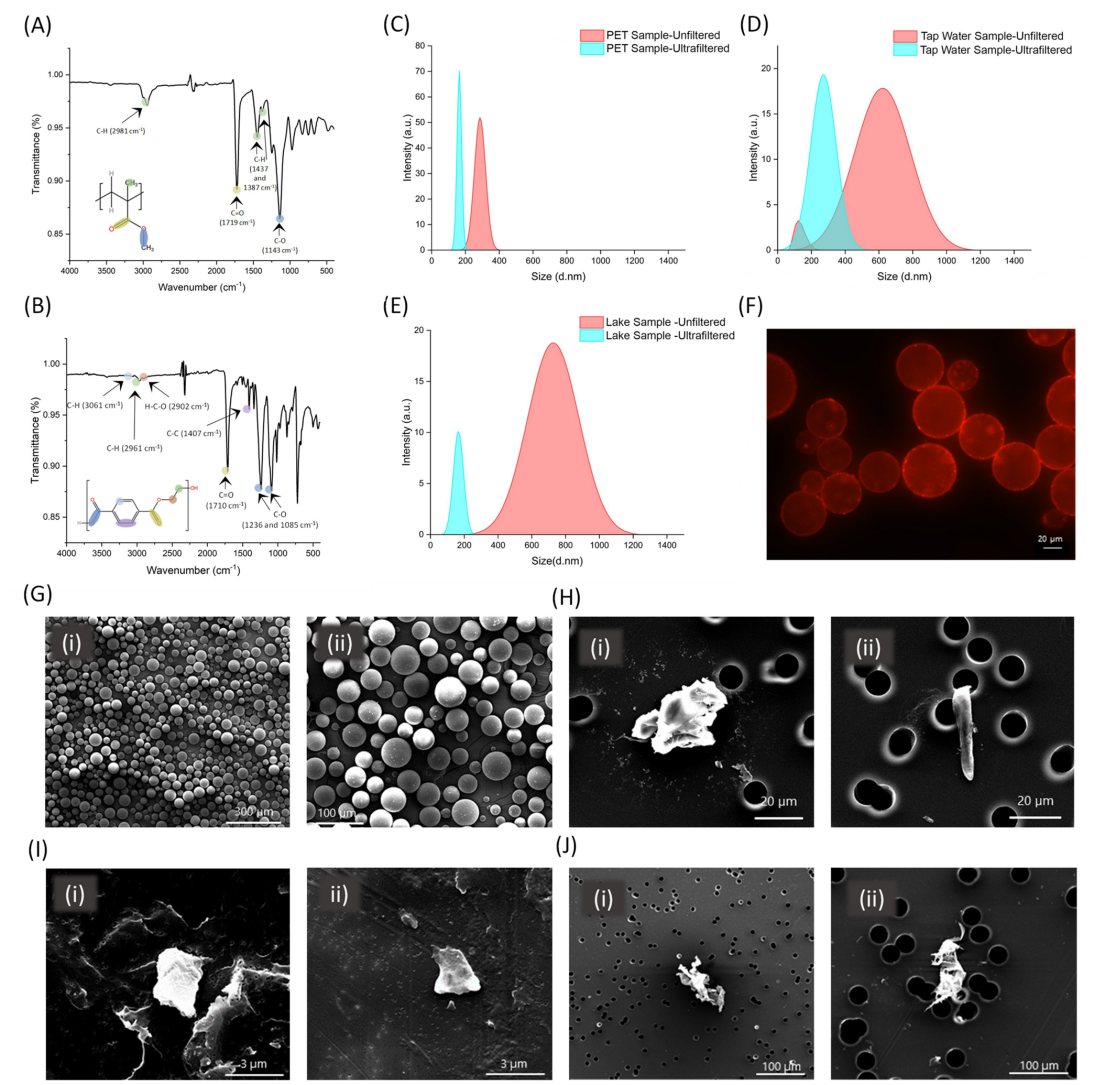
FTIR analyses of (A) PMMA and (B) PET MPs are presented. (C) Size distribution (d. nm) and intensity (%) of (C) PET, (D) tap MPs, and (E) lake MPs before and after ultrafiltration steps are measured. (F) Nile Red-stained PMMA MPs are imaged using an inverted microscope. SEM images of (G) PMMA MPs, (H) trapped PET MPs, (I) tap MPs, and (J) lake MPs are demonstrated.

In the chemical characterization, the characteristic peaks of functional groups were analyzed to elucidate the molecular structures. Both PMMA and PET polymers consist of ester functional groups. Moreover, this analysis aims to detect the C–H aromatic bonds expected from the benzene ring in PET MPs, alongside the C–H aliphatic bonds present in both polymers. For PMMA, two significant indicative peaks of the ester functional group were expected in its molecular structure (Figure 3A). As a result, the carbonyl (C=O) peak at 1719 cm^–1^ corresponds to this functional group. The sharp peak at 1143 cm^–1^ represented the C–O bond associated with another ester group. Furthermore, the peak at 2981 cm^–1^ supported the presence of C–H aliphatic bonds. In PET characterization (Figure 3B), a peak attributed to aromatic C–H bonds was observed at 3061 cm^–1^. The C–H aliphatic bonds appeared at 2961 cm^–1^. Similar to PMMA, PET exhibited characteristic ester peaks. The carbonyl (C=O) peak was detected at 1710 cm^–1^. The peaks at 1236 and 1086 cm^–1^ confirmed the presence of C–O bonds within the ester groups, attributable to the two distinct ester environments in the PET molecule.

Next, PET, tap water, and lake samples were initially filtered using an ultrafiltration chip equipped with 10 and 2 μm cellulose-based filters, respectively. Before the ultrafiltration, we measured PET MPs as 285.46 ± 116.62 nm, and this was further reduced to 164.52 ± 17.6 nm postfiltration (Figure 3C). In all measurements, we used the hydrodynamic range measured with Dynamic Light Scattering (DLS, Zetasizer Nano ZS, Malvern, Country). For the tap water samples (Figure 3D), the hydrodynamic size range changed from 625.76 ± 556.84 nm to 273.24 ± 254.13 nm after the ultrafiltration. Likewise, in the lake samples, the initial hydrodynamic size range of 724.35 ± 492.38 nm was refined to 164.52 ± 86.38 nm once ultrafiltration was performed (Figure 3E). This fine-tunning in size distributions of MPs is critical for an accurate observation of smaller MPs under a microscope. Larger MPs can potentially interfere with the detection of smaller MPs due to the intense fluorescence they emit. Additionally, different polymers and living organisms may interfere with Nile Red measurements as previously reported,^84^ and hence, the ultrafiltration chip will have significant improvement in the sample preparation step.

In order to establish a versatile filtration protocol before fluorescence microscope analyses, Nile Red was utilized to stain all of the samples for 15 min at 80 °C after the ultrafiltration process. Since PMMA was purchased commercially, we did not ultrafiltrate but only stained them. Subsequently, it was examined under an inverted microscope, and stained PMMA MPs were employed to confirm the staining procedure (Figure 3F). To determine whether there had been any polymer degradations at the incubation temperature, nonstained PMMA and PET MPs were examined using the TGA instrument. PMMA and PET were shown to begin to degrade at 254 and 385 °C, respectively (Supporting Information Figure 2). Afterward, the images of PMMA (Figure 3G), the filtered PET (Figure 3H), the filtered tap water (Figure 3I), and the filtered lake water (Figure 3J) were captured for morphological analysis using SEM. This disintegration process was the reason for the structural defect in PET MPs obtained using a homogenizer, even though PMMAs produced in the form of beads may be easily differentiated.

### Comparative Detection of MPs

To assess the efficacy of MicroMetaSense, we compared their results to those derived from the commonly used glass substrate in microscopy. Initially, Nile Red-stained MPs were drop-casted onto both glass and the MicroMetaSense platform at a volume of 10 μL, and the MPs were observed under an upright microscope at 100× magnification. ImageJ software was used to calculate gray values in the region of interest (ROI) by using the histogram command. The generated histogram represents the unweighted 8-bit gray values with respect to the pixel distribution. ImageJ converts individual RGB pixels into unitless grayscale intensity values ranging from 0 to 250 using the following formula.^85,86^
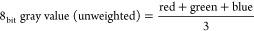
1

These grayscale intensity values were used to evaluate the fluorescence intensity in the images. All analyzed images were captured under consistent parameters. The effectiveness of MicroMetaSense versus a conventional glass substrate was compared by determining the EF values. To calculate the EF, background signals (N_G_ for glass and N_M_ for metasurface) from each surface were measured as outlined in eq 2. For these calculations, signals from five different points on the glass (S_G_) and metasurface (S_M_) from each MP source were analyzed to obtain EF values (Table 1).
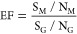
2

**Table 1:** Enhancement Factors (EFs) of Five Different MP Sources

Sample	EF 1	EF 2	EF 3	EF 4	EF 5	
PMMA	3.40	2.65	2.28	1.73	1.22	supporting information figures 5–9
PET	8.79	5.94	3.40	2.78	1.87	supporting information figures 10–14
Real tap water	6.52	4.90	2.63	2.06	1.42	supporting information figures 15–19
Real lake water	1.68	1.61	1.30	1.27	1.21	supporting information figures 20–24
Artificial ocean water	3.78	8.97	12.11	23	36.56	supporting information figures 25–39

**Figure 4:**
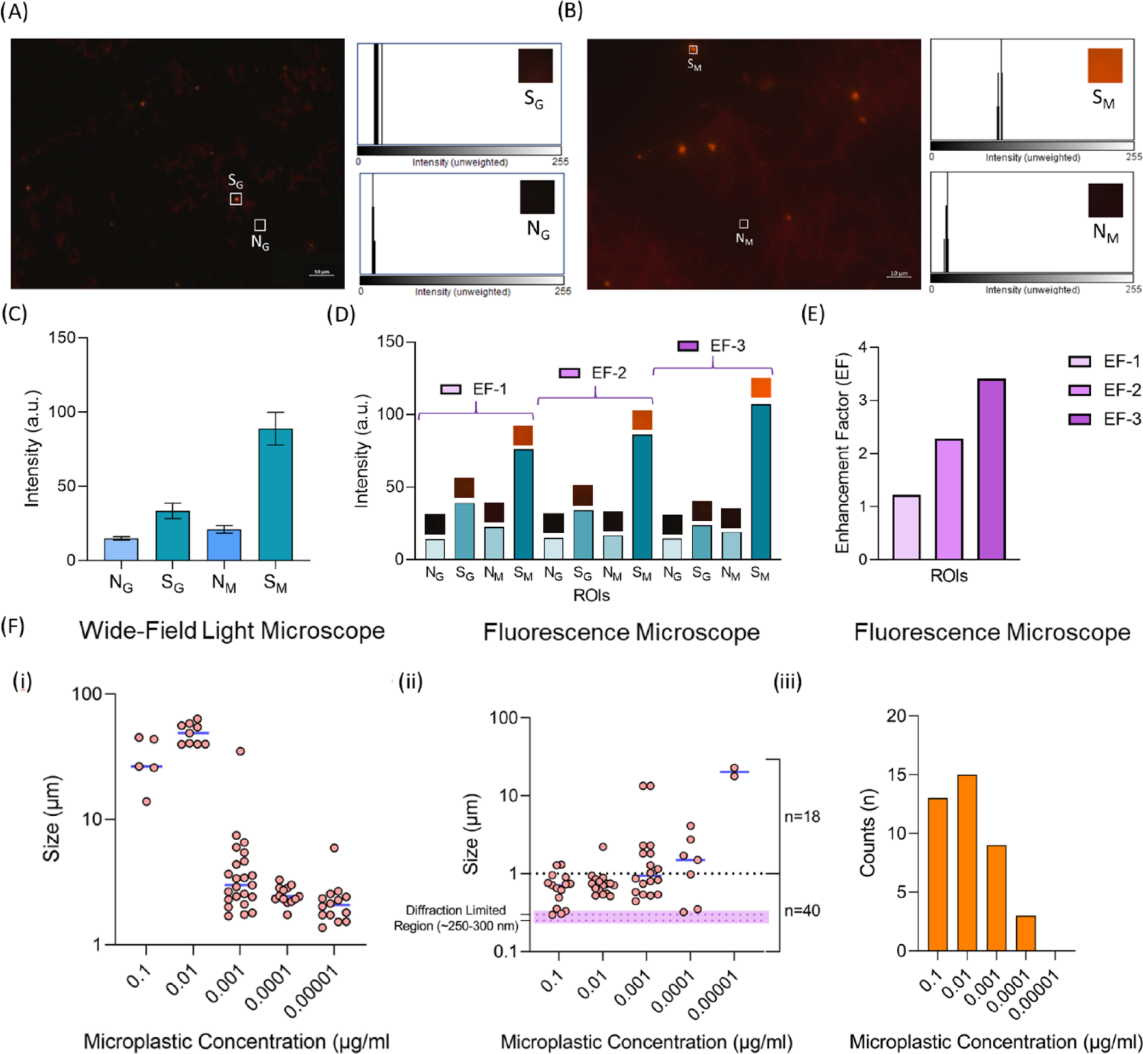
Upright fluorescence imaging and size analysis of PMMA MPs are presented. The fluorescence images of Nile Red-stained PMMA MPs are examined under (A) glass substrate and (B) MicroMetaSense at 100× magnification. The fluorescent images include two different types of ROIs: one for measuring the MP fluorescent signal (S) and another for measuring the background noise (N). The grayscale intensity values in these ROIs are measured for MP fluorescence signal (S_G_ and S_M_) and background noise (N_G_ and N_M_). The histogram plots of the measurements are placed next to the fluorescence images. (C) Mean value of grayscale intensity values of N_G_, S_G_, N_M_, and S_M_ for PMMA MPs are plotted in a bar graph using five different ROIs for each S and N. (D) EFs on a set of ROIs are demonstrated, capturing S and N for PMMA MPs on both the glass substrate and MicroMetaSense. (E) Calculated EFs for PMMA MPs are demonstrated on a bar graph. (F) The number of PMMA MPs are visually sorted by the particle size across a range of serial dilutions (up to 10^4^ times dilution factor). The size distribution of PMMA MPs is determined on a micron scale using (i) wide-field light microscope and down to diffraction-limited region (250–350 nm) using (ii) fluorescence microscope. (iii) The number of detected submicron PMMA NPs at each dilution is separately displayed on a bar graph.

In the initial analysis, the observability of PMMA MPs on glass (Figure 4A) compared to that of the MicroMetaSense (Figure 4B) was noticeable. The impurities appeared more distinctly in the microscope images, indicating that the implementation of MicroMetaSense for MP detection is a promising strategy. Consistent with these observations, the average intensity values of MPs were measured at 32.38 a.u. for glass and 91.47 a.u. for MicroMetaSense (Figure 4C). Additionally, the average background signal values were 14.98 a.u. for glass and 20.16 a.u. for MicroMetaSense. It is claimed that the difference in signal intensity between background signals (N_M_ and N_G_) is much lower than the difference between MPs’ signals (S_M_ and S_G_). The highest intensity value among PMMA MPs was recorded as 107.11 a.u. on the MicroMetaSense, compared to 39.2 a.u. on the glass substrate. Consequently, the EF range for PMMA measurements was calculated to be between 1.22 and 3.40 a.u. (Figure 4E). Considering PMMA MPs, MicroMetaSense demonstrated superior sensitivity and detection limits, enhancing the measurement by a factor of 3.40.

PMMA MPs were sorted based on their smallest detectable size using both wide-field light microscopy and fluorescence microscopy across various dilution ratios (10–10^6^ times). The distribution of PMMA MPs demonstrated distinct variations between the two methods, underscoring the distinct capabilities of each technique. Wide-field optical microscopy enabled the determination of MP sizes ranging from 1 to 100 μm (Figure 4F-i), whereas fluorescence microscopy could also be able to detect particles smaller than 1 μ, within a detection range extending from 300 nm (diffraction-limited region) to 30 μm (Figure 4F-ii). As a result, fluorescence microscopy with MicroMetaSense as a substrate enabled the examination of NPs (Figure 4F-iii) due to enhanced optical contrast from the MEF between the MicroMetaSense and Nile Red. This finding was significant, as recent literature has emphasized the potential health impacts of NPs, which could initiate various interaction pathways.^87^

**Figure 5:**
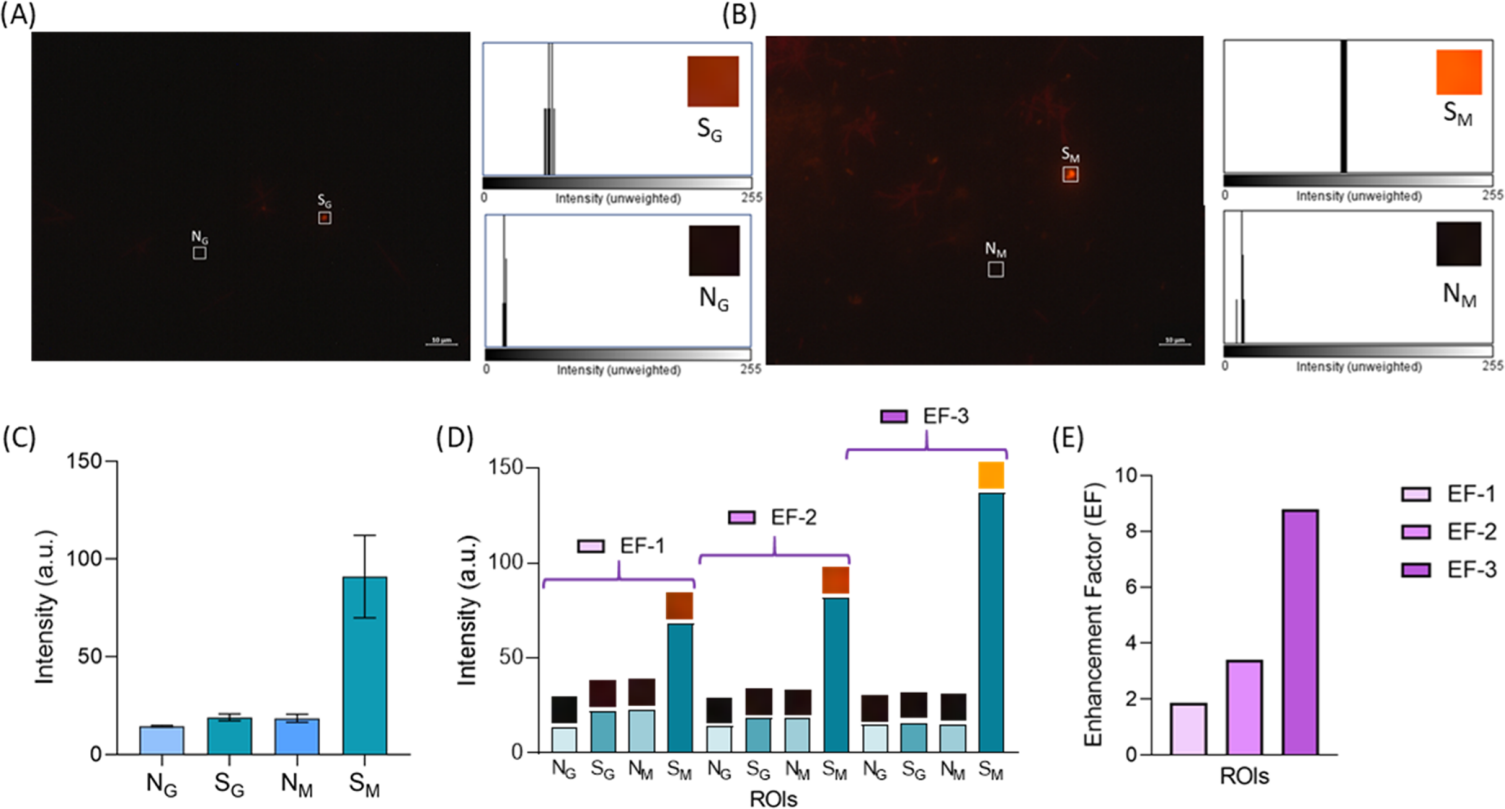
Upright fluorescence imaging of PET MPs is presented. The fluorescence images of Nile Red-stained PET MPs are examined under (A) glass substrate and (B) MicroMetaSense at 100× magnification. (C) Mean values of grayscale intensity of N_G_, S_G_, N_M_, and S_M_ for PET MPs are plotted in a bar graph using five different ROIs for each S and N. (D) EFs on a set of ROIs are demonstrated, with S and N for PET MPs captured on both the glass substrate and MicroMetaSense. (E) Bar graph provides a summarized depiction of the EFs for the PET MPs.

Regarding the PET samples, we observed a very distinguishing effect while analyzing these MPs with the MicroMetaSense compared to the glass substrate (Figure 5A,B). Here, the average intensity values of MPs were measured as 18.78 and 95.49 a.u. for glass and metasurface, respectively, while the background averages of these surfaces were calculated as 14.42 and 18.36 a.u. Likewise in PMMA, while the difference between background signals was quite low, the average intensity difference in PET MPs was quite higher compared to PMMA MPs. This difference was also observed between the highest intensity values between PMMA MPs and PET MPs. For the case of PET MPs, the highest intensity on the MicroMetaSense was measured as 137 a.u. This difference in the highest intensity value between PMMA and PET may be due to the shape of the MPs. Deformities on the PET surface (Figure 3H) may cause an increase in the fluorescence reflections. Therefore, the EF range for PET was calculated as 1.87–8.79 a.u.

**Figure 6:**
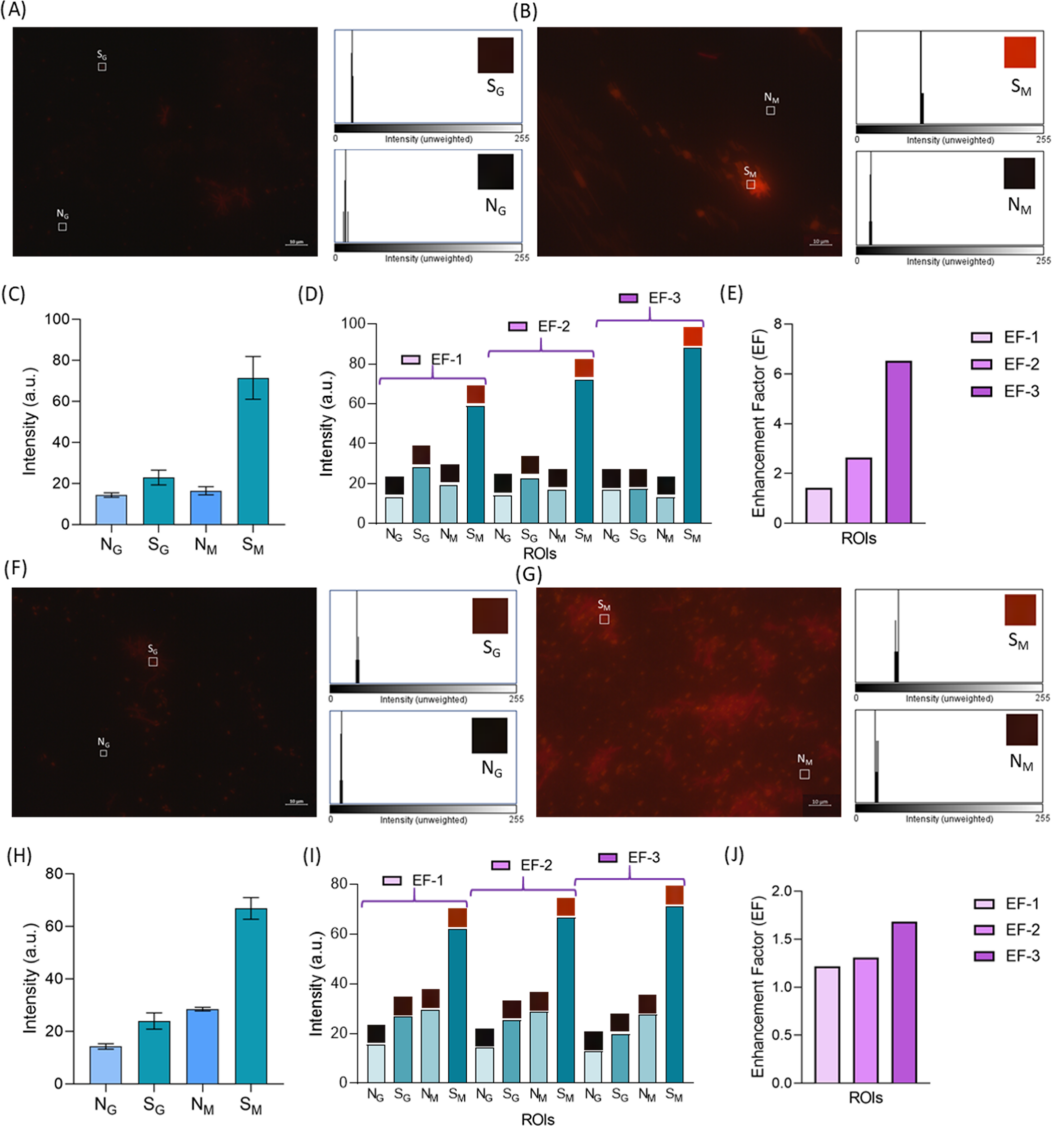
Upright fluorescence imaging of tap and lake samples are presented. Nile Red-stained tap samples are examined under (A) glass substrate and (B) MicroMetaSense at 100× magnification. (C) Mean value of grayscale intensity values of N_G_, S_G_, N_M_, and S_M_ for tap samples are plotted in a bar graph using five different ROIs for each S and N. (D) EFs on a set of ROIs are demonstrated, with S and N for tap samples captured on both the glass substrate and MicroMetaSense. (E) Bar graph provides a summarized depiction of the EFs for tap samples. Nile Red-stained lake samples are examined under (F) glass substrate and (G) MicroMetaSense at 100× magnification. (H) Mean values of grayscale intensity values of N_G_, S_G_, N_M_, and S_M_ for lake samples are plotted in a bar graph using five different ROIs for each S and N. (I) EFs on a set of ROIs are demonstrated, with S and N for lake samples captured on both the glass substrate and MicroMetaSense. (J) Bar graph provides a summarized depiction of the EFs for lake samples.

**Figure 7:**
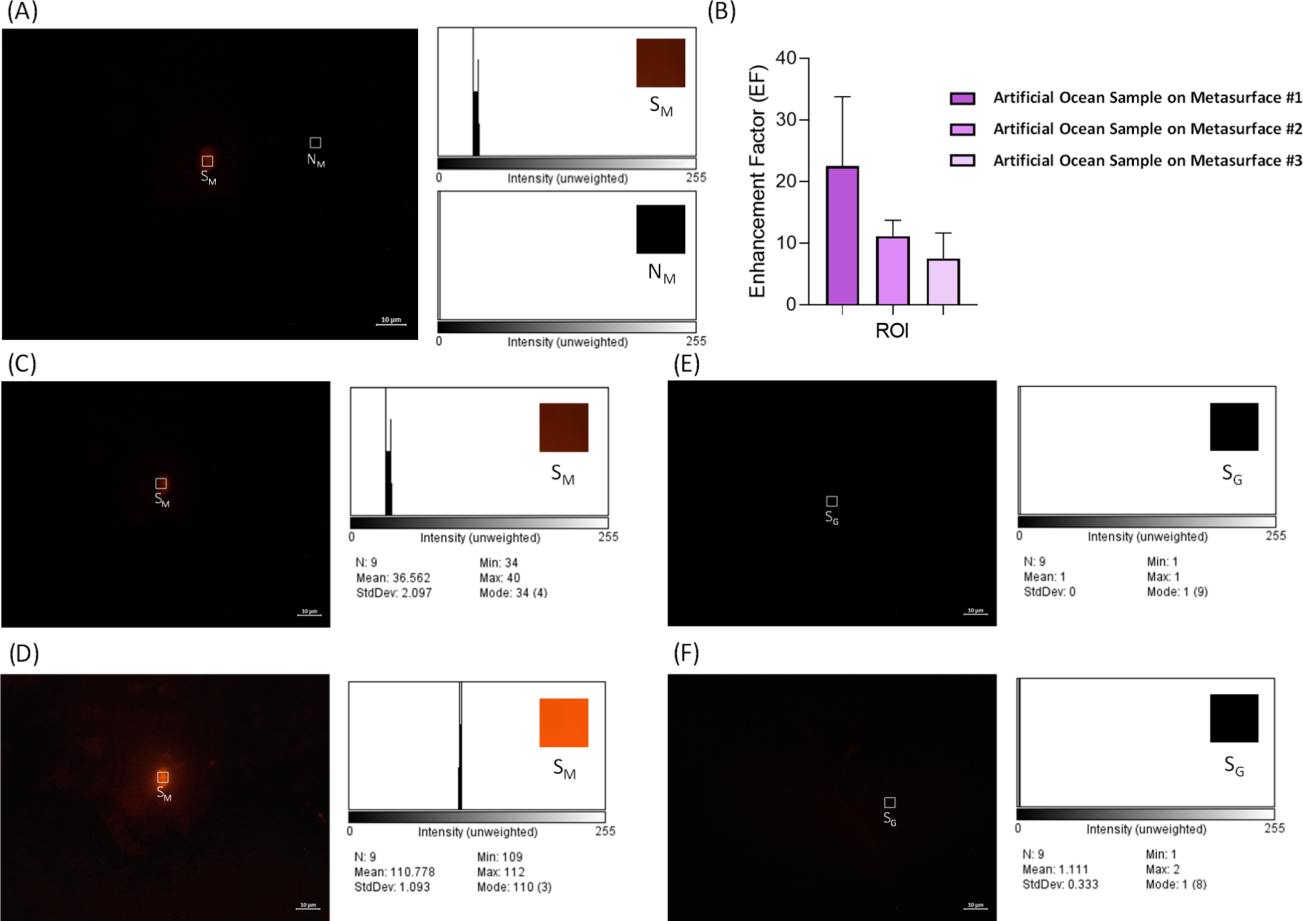
Upright fluorescence imaging of MP-loaded artificial ocean water is presented. (A) S_M_ and N_M_ intensity values of MPs on MicroMetaSense are calculated. (B) Using three MicroMetaSense surfaces produced by the same method, five measurements per surface are taken. (C) 20% and (D) 100% intensity levels are utilized to observe the MPs on MicroMetaSense. (E) A 20% and (F) 100% intensity levels were utilized for MP detection on glass, but no MPs are observed.

Finally, the most significant performance evaluation of this system was conducted using tap water (Figure 6A,B) and lake samples (Figure 6F,G) collected as real-world samples. Moreover, artificial ocean water (Figure 7A–F) was prepared to observe the effect of salty water on MP detection. While significant EFs for MicroMetaSense were observed in the previous MP sources, it is imperative to validate its efficacy with real samples to underscore its potential for future applications. Regarding the tap water sample, the average intensity values of MPs were recorded as 21.98 a.u. for glass and 73.16 a.u. for MicroMetaSense (Figure 6C). Background signals were measured at 14.58 a.u. for glass and 16.26 a.u. for MicroMetaSense, indicating a low background interference. The highest intensity in the tap water was 88.33 a.u. for MicroMetaSense, compared to 17.3 a.u. for the glass substrate (Figure 6D). The EF range for the tap sample was calculated to be 1.42–6.52 a.u., demonstrating the superior performance of MicroMetaSense with real samples.

Similarly, regarding the lake samples, average intensity values were 23.94 a.u. for the glass substrate and 66.82 a.u. for MicroMetaSense (Figure 6H). Although a significant difference was observed between the intensity averages, a corresponding difference was also noted in the background signals (14.28 a.u. for the glass substrate and 28.47 a.u. for MicroMetaSense), leading to a reduced EF range compared to other MP sources. This reduction was primarily attributed to the presence of fiber-like structures in lake samples, originating from plants such as moss, which increased the background signal and affected the MicroMetaSense’s efficiency in detecting MPs. Consequently, the highest signal value in the lake samples was 71.33 a.u. for MicroMetaSense and 26.9 a.u. for the glass substrate, resulting in an EF range of 1.21–1.68 a.u. (Figure 6I). To mitigate this challenge in lake samples, the previously employed ultrafiltration on-a-chip process would be repeated using filtration papers with smaller pore sizes to eliminate unwanted fibers, or a multichannel filtration strategy would be adopted in future studies.

In the final MP analysis, the initial salt concentration of an MP-loaded artificial ocean solution prepared with laboratory standards was set at 3%. Under the microscope, no MPs were observed with either the use of MicroMetaSense or glass due to the dense salt crystals. Therefore, the artificial ocean solution was diluted at a ratio of 1:100, and the microscope analysis was conducted again. As a result of this dilution, MPs were observed on MicroMetaSense at both 20 and 100% intensity levels (Figure 7A). Since a 20% intensity level was utilized in all other analyses, the measurements for the artificial ocean sample were also conducted using this intensity. Unlike the other measurements, three MicroMetaSense surfaces were produced using the same production method and five measurements were taken using each surface. The high observability of MPs in each measurement demonstrated the reproducibility of the production method. The differences in measurement results were affected by the distance of the MP from the MicroMetaSense surface, the Nile Red binding rate, and the size of the MP (Figure 7B). As a result, the highest MP intensity value was recorded as 36.562 a.u. (S_M_) (Figure 7C). The background signal (N_M_) was calculated as approximately 1 a.u.. On the other hand, no MPs were observed on the glass surface at both 20% and 100% intensity levels, and all intensity measurements were close to 1 a.u. (Figure 7E,F). Based on these results, the EF values of MPs found in ocean water were calculated between 3.789 and 36.562 a.u. Additionally, when the intensity value was increased from 20 to 100%, while no MPs were observed on the glass surface, the intensity value on the MicroMetaSense surface increased from 36.562 to 110.778 a.u. (Figure 7D). As with previous analyses, the performance of MicroMetaSense in ocean water significantly enhanced the detectability of these pollutants.

As stated in earlier studies,^63,88^ we herein calculated the MP analyte mass for determining the LOD and detection range parameters. In our study, the ROI volume was selected as 3 × 3 × 3 pixels for an isolated MP quantification, considering a cubical volume of the fluorescence signal and 3D symmetry of the selected individual MP. The equivalent volume for 3 × 3 × 3 pixel was determined as 0.155 μm^3^ and the density of the MPs for PET and PMMA were assumed as 1.38 and 1.18 g/cm^3^, respectively. Using these values, the LOD values were calculated as 205 and 183 fg for PET MPs and PMMA MPs, respectively. This approach relies on many technical factors, including pixel amount in a single ROI volume, detector type, and filter type. Furthermore, the integration of the detection limit approach with super-resolution microscopy could improve the LOD values to the single molecule level.^89^ On the other hand, in addition to LOD, the reproducibility of our platform could be affected by various factors, including Nile Red staining efficiency, potential thickness defects of the metasurface coming from the production challenges, and aggregation of MPs that could change the signal measured from similar size-range particles. This challenge could be addressed by also automating the Nile Red staining protocol, as well as the metasurface production. On the other hand, MicroMetaSense protocol does not include the reuse of the metasurface to prevent contamination of particles from previous measurements. An advanced cleaning protocol could also be adopted in the future for metasurfaces to further reduce experimental costs.

In future studies, two important aspects of this work can be developed. A key step in these developments is the high-efficiency extraction of MPs from environmental samples. In order to achieve this, Faramarzi et al. captured MPs using the surface nanodroplets method in a microfluidic system at low flow rates.^33^ This approach presented a simpler system compared with the complex purification methods. Integrating this system into the microfluidic filtration that we conducted can provide a significant advantage in the elimination of various algae and wood particles that are found in lake and ocean samples.

In addition to the extraction and detection of MPs, the fluorescence enhancement phenomenon can be utilized for some chemicals present in the environment. For instance, Thacharakkal et al., for instance, observed a 1517-fold enhancement in the detection of the perfluoroalkyl substance (PFAS), specifically perfluorooctanesulfonic acid (PFOS), which poses serious health risks.^90^ In this study, Ag–Au (gold–silver) heterometallic nanohybrids were produced to utilize the photothermal properties of porous nanocarbon frameworks (NCFs). These heterometallic structures significantly increased the detection aspect, enhancing the surface-plasmon-coupled emission (SPCE) property.

Moreover, not only for pollutants but also for monitoring important drugs such as perindopril erbumine, Bhaskar et al. achieved a 310-fold enhancement in SPCE using the nanocarbon florets (NCFs), enabling the detection at the attomolar level.^91^ Integrating these materials and filtration methods into our system holds potential to expand the applications’ limits. The binding of these materials to the produced MicroMetaSense surface and the integration of a low-cost, portable, and high-efficiency MP ultrafiltration method throughout the system can significantly enhance the detection of these MPs in the future.

## Conclusions

In this study, we demonstrate a holistic strategy including an ultrafiltration on-a-chip and MicroMetaSense platform in order to resolve challenges in the size distribution and sensitivity of MPs. Prior to the ultrafiltration process, MPs were distributed in sizes of 285.46 ± 116.62 nm, 625.76 ± 556.84 nm, and 724.35 ± 492.38 nm for PET spiked in water, tap water, and lake samples, respectively. Once they were pressed through the chip, we were able to improve the size distribution and standard deviations of MPs down to 164.52 ± 17.6, 273.24 ± 254.13, and 164.52 ± 86.38 nm for these samples, respectively. These numbers were smaller than those of our filter pore sizes, pointing out a successful ultrafiltration process. In addition, the intense emission led by larger MPs (1–5 μm) and their larger surface area compared to smaller MPs (<1 μm) were eliminated with this procedure, as well as any microscopic organism (e.g., bacteria and algae) that may potentially present and interfere with Nile Red staining. The current filter configuration of the chip was only demonstrated as the model system, and it would also be modified with different pore sizes of filter papers for targeting smaller MPs if interested. In addition, in the future, this strategy would be employed for continuous monitoring of MPs without any significant interference of larger contaminants.

In addition, MicroMetaSense enabled us to detect and determine small sizes of MPs and NPs (down to 250 nm in size), which can potentially penetrate into cells. In order to understand any advances in terms of the size parameter, the measurements were taken under the same conditions on glass surfaces—frequently utilized strategy as a conventional method. The fluorescence measurements for each surface were collected, and subsequently, the fluorescence signals were calculated in terms of the Nile Red emission and the background noise (eq 1). After that, EF was calculated, and we demonstrated superior signals observed from the MicroMetaSense platform by comparing the conventional method. As a result, we calculated 3.40 (PMMA), 8.79 (PET), 6.52 (tap water), 1.68 (lake), and 36.56 (artificial ocean water) times the emission enhancements for spiked samples (PMMA and PET) and real samples (tap water, lake water, and artificial ocean water), respectively.

Considering the cost perspective of the entire strategy, in the first part, a reusable (up to 5 times) microfluidic system implemented for MP filtration costs around $1. Additionally, MicroMetaSense, which enables the detection of smaller MPs and even NPs through the MEF phenomenon, costs only $1. This represents a significantly lower expense compared with the high cost associated with common lithography methods. Accordingly, the entire system is expected to cost a total of $2. Beyond affordability, these methods are easy to operate, with the production stages that are clean-room-free and do not need any harmful chemicals. Such ease of production supports widespread use of these systems.

In addition to its cost efficiency, the proposed system offers substantial time savings. In detail, the filtration system is completed in under 20 min, providing a significant advantage over the long-lasting duration of conventional methods. While only the sample preparation step with a centrifuge^92^ or incubator^93^ takes 30 min, the sample is prepared for MicroMetaSense in ∼10 min, making the total detection process around 30 min, which is significantly lower when we compare the conventional systems.

On the other hand, the presented strategy combining the ultrafiltration process and MicroMetaSense is still in development, and in the future, we consider that the following updates in the design and workflow would improve its applicability in a broader sense. First, the current microfluidic-based ultrafiltration chip contains filter papers with pore sizes of 10 and 2 μm. A general challenge for filtration processes, clogging, can be observed as a result of the accumulation of large-sized microplastics and other contaminants on these filter papers. Including a prefiltration chamber with larger pore sizes rather than the current design containing only two filtration chambers may facilitate the elimination of larger fragments. As we demonstrated for bionanoparticles earlier,^31^ backwashing would be another potential solution for minimizing the clogging challenge. Second, despite MicroMetaSense having achieved the detection of MPs and NPs with high sensitivity, the current platform is not compatible with real-time measurements. This asset would be enabled with video recordings for more detailed, timely investigations. In this regard, a transparent microfluidic platform can be utilized for real-time assessment of the Nile Red-stained MPs under a fluorescence microscope. Third, in relation to this real-time measurement, although the microfluidic system and MicroMetaSense can fit in the palm of a hand and have the potential to be used in the field, they are not portable. The integration of portable micropump devices for a filtration stage and a miniaturized imaging system for MicroMetaSense would play a crucial role in making this system fully portable and autonomous. Lastly, different types of MP dyes that have changing colors according to the origin of the MP material would also be adopted for the system for making multichannel measurements for different MPs from the same source.

Overall, MicroMetaSense presents many advantages for MP detection including the reduced experimental effort (under 30 min with 4 simple steps), cost-effectiveness ($2), and improved sensitivity (183–205 femtogram of MPs). When compared to the most-cited literature studies, it is shown that MicroMetaSense is one of the few studies that could provide submicron detection of microplastics in a broad size range (250 nm to 100 μm) and with minimal instrumentation (only a standard fluorescence microscope and laboratory supplies) (Supporting Information Table 1). Although MicroMetaSense is not capable of detecting microplastic types, the overall platform provides an easy to use, low-cost, and widely deployable strategy to monitor environmental samples for further analysis with plastic type and origin. The current methodology for microplastic detection includes standardized and well-documented methodologies that are also shown in ISO guidelines and EU Regulations. AFNOR guideline (XP T 90-968 1 Water quality), ISO guidelines, and EU Regulations on microplastic detection recommend different techniques including micro-FTIR, micro-Raman, LDIR, or combination of analytical techniques such as Py-GC/MS.^94,95^ However, the requirement of skilled personnel for their proper operation and expensive instrumentation hinder the expansion of these technologies and limit the sample number that can be processed per turn. Rather than being a standard methodology, fluorescence microscopy could be adopted at this point as a rapid screening method and could be deployed for MP detection easily for a later stage of sample verification with analytical techniques. MicroMetaSense, hence, would be employed to test many samples possible in a short time for estimating the amounts of microplastics and deciding which samples should be transferred to the standard workflow. This will reduce the effort and burden in classical methodologies and will be a useful alternative for marking microplastic-contaminated samples. In addition, reuse of both the ecofriendly microfluidic system and the off-the-shelf products as sensor surfaces also brings the process closer to a self-sustainable and environmentally friendly workflow for MP detection or pre-evaluation of the samples. The main advantage of the microfluidic system used in this study is that, although classical methodologies require filtration of the large volume of samples in liters, our platform could even analyze a few milliliters of the sample, which could decrease sample collection efforts and could increase samples processed from a source for more accurate MP assessment. If required, the microfluidic platform could be connected for a parallel flow to reduce the isolation time for a larger volume of samples. Due to the simple design of the microfluidic chip, it can be easily disassembled for cleaning in order to reuse the chip components. The way to fabricate these chips also allows the constructed platform to be reusable since the DSA film is not permanently bonding 3D filter structures and filters. Although commercial filter systems (syringe filters or pump-based filtration systems) could be used to filter microplastic samples sequentially as we applied in our design, they are not cost-effective, usually built for single use, and their accessory parts cause more plastic waste. As a prominent advantage, our microfluidic ultrafiltration platform could be built from plastic-free materials and prevent increasing plastic waste generation from the laboratories. In addition, after usage, all of the parts, except the DSA layers, could be cleaned and used for rebuilding the chip for many rounds of usage. One should also keep in mind that there would be potential contaminations. Hence, single-time use of these chips would be more beneficial, as the chip fabrication is facile and inexpensive. In conclusion, this study sheds light on portable MP detection strategies in future studies.

## Materials and Methods

### Fabricating MicroMetaSense

The grating structure on an optical disc was conducted as described in our previous study.^81^ For the metasurface template, an HP brand digital versatile disc recordable (DVD-R) was used. First, the plastic layer was carefully removed with a knife. Next, the metal reflector layers was detached using pressurized air, and the remaining dye was eliminated with a 1:1 mixture of ethanol and methanol. This was followed by a 1 min etching process in a 1:4 mixture of acetone and isopropanol. Subsequently, the optical disc underwent a sequential coating process involving the deposition of titanium (10 nm), silver (30 nm), and gold (15 nm) via thermal evaporation (PVD-Vapor-3S_Th, Vaksis R&D and Engineering, Turkey) as schemed in Figure 2B. The initial titanium layer was employed, owing to its adhesive properties, facilitating the deposition of subsequent metal layers. The intermediate silver layer, in conjunction with the gold layer, establishes plasmonic resonance. The gold layer, in particular, is selected not only for its contribution to plasmonic resonance but also for providing an inert surface, which is essential for interaction with samples and the surrounding medium. The resultant metasurface was then cut into smaller sections (1 cm x 1 cm) and affixed to microscopic slides for the examination of MP samples.

### Characterizing MicroMetaSense

The surface morphology of MicroMetaSense was characterized by using atomic force microscopy (AFM, Asylum, Oxford Instrument, UK) and scanning electron microscopy (SEM, Quanta 200F, FEI). Both techniques were employed in accordance with established standard protocols for device characterization.

### FDTD Simulations

The reflection spectrum of the MicroMetaSense was modeled employing a finite-difference time-domain (FDTD) software package (Lumerical Inc.). The simulation model was reliant on a 2D geometry of a unit cell structure, comprising a grating substrate with dimensions of 400 nm width, 30 nm height, and a periodicity of 740 nm. Periodic boundary conditions were implemented along with a TM-polarized plane source. The optical properties of the metal layers were derived from Palik’s handbook,^89^ while the refractive index of the PC substrate was specified as 1.58.

### Preparing Microplastic Samples

Commercial PMMA beads used in this study were purchased from a commercial supplier, offering a range of plastic beads from nanometer to millimeter scales. PMMA MPs were prepared by dispersing 10 mg of PMMA beads in 1 mL of ddH_2_O to adjust the concentration to 1%. This stock solution was subsequently diluted to obtain concentrations of 0.1%, 0.01%, 0.001%, 0.0001%, and 0.00001%. On the other hand, for making PET MPs, a commercial water bottle was cut into fragments using scissors and further reduced in size using an Ultraturrax homogenizer (ISOLAB, Germany) operating at 10,000 rpm for three cycles of 1 min intervals. The homogenized samples were then filtered using a Whatman filter paper prior to initial testing. Moreover, in order to investigate MPs in real-world settings, samples were collected from lakes and tap water. The samples were then taken into 50 mL Falcon tubes and filtered through Whatman paper to remove extraneous particles, such as debris. On the other hand, MPs were spiked in the artificial ocean sample. For 100 mL of artificial ocean water solution with 3% salt^96^ and 0.1% MPs concentration, 0.38 g of magnesium chloride (MgCl_2_) and 2.62 g of sodium chloride (NaCl) were dissolved using a magnetic stirrer. Then, 100 mg of PMMA MPs were added to obtain MP-contaminated ocean water samples. All samples were stored at 4 °C for further characterizations and analyses.

### Chemical Characterization of MPs

PMMA and PET MPs were characterized by FTIR (Bruker Tensor 37, Germany) spectroscopy for chemical verification. For this characterization, 100 mg of each sample was dried using an oven at 50 °C for 24 h. Attenuated total reflectance (ATR) mode was utilized for the chemical characterization. After the surface of the ATR crystal (diamond) was cleaned using isopropyl alcohol (IPA), each MP was analyzed by utilizing 128 scans. Moreover, thermogravimetric analysis (TGA) (TA Instruments Q500) was carried out to observe any heat-related decomposition during the staining of these targeted impurities with Nile Red (Sigma-Aldrich). This heating process was carried out from 35 to 950 °C with an increase of 10 °C per minute. In this decomposition analysis, first, the surface of the platinum pan was cleaned using a blowtorch. After taring the platinum pan using the instrument’s software, 8 mg of each sample was weighed and analyzed, respectively.

### Optimizing MP Size Distribution via Ultrafiltration On-a-Chip

An ultrafiltration microfluidic chip system was designed to optimize the MP size distribution. The ultrafiltration on-a-chip was developed using Shapr3D software (Shapr3D, Hungary) as shown in an earlier study.^31^ The generated models were subsequently 3D-printed using the Halot One CL-60 (Creality, China) device with resin parameters provided by the manufacturer (Anycubic, China) for a plant-based, UV-curable, clear resin. After production process, the chip components were cured for 25 min in the Creality UW-02 device. The assembled chip parts were combined using a 0.2 mm double-sided, optically clear adhesive DSA layer (8146-2-ND, 3M), which was precisely cut with a laser cutter (LazerFix, Türkiye) according to the 2D blueprints exported from Shapr3D software as .dxf files and transferred to the RDWorksV8 software (RuiDa Technology, China) for laser cutting operations (Supporting Information Figure 4). The laser cutter parameters were set to 100% speed and 20% of power. PET, lake, and tap water samples were processed through this chip using a syringe pump at a flow rate of 40 μL/min, employing 10 and 2 μm filter papers to achieve a more precise size distribution. Additionally, 0.65 mm polytetrafluoroethylene (PTFE) tubing (Adtech Polymer Engineering, UK) was used to facilitate the filtration process.

### Dynamic Light Scattering Analysis

DLS (Zetasizer Nano ZS, Malvern, Country) instrument was utilized to demonstrate any improvements in the size distribution of PET, lake, and tap samples once they were processed through an ultrafiltration step. For this purpose, DLS analysis was operated with water as a dispersant (viscosity cP: 1.0016, refractive index: 1.330, temperature: 20 °C) and 120 s of equilibration time and 25 °C as the equilibration temperature in a ZEN0040 disposable cell. The refractive index value for PET measurements is 1.636, and the absorbance is 0.010. Since the ingredients of the lake and tap samples were unknown, measurements were carried out without any refractive index and absorbance value.

### Scanning Electron Microscopy Analysis

For the analysis of PMMA MPs, beads were initially sampled directly onto a carbon tape. Afterward, PET, lake, and tap samples were analyzed after the ultrafiltration. The filtration papers utilized in the ultrafiltration chip were glued directly to the carbon tape to observe the impurities captured in the ultrafiltration process. In addition, 10 μL of other samples in solution were taken, dropped onto a carbon tape, and dried in a chemical hood. All samples were coated with 10 nm gold–palladium (Au–Pd) with a Gatan 682 precision etching and coating system (PECS). Imaging was operated with an FEI Quanta 200F device (FEI) using a 10.00 kV acceleration speed and a 3.0 spot size.

### Comparative Analysis of MPs with a Conventional Method and MicroMetaSense

Nile Red (Sigma-Aldrich) was utilized to visualize the MPs under two different microscopy setups. Nile Red has excitation and emission wavelengths of 460 and 525 nm, respectively.^97^ The dye stock solution was prepared by dissolving 10 mg of Nile Red in 10 mL of methanol. For staining the MPs, a volumetric ratio of 1:100 (v/v) was used, and the samples were incubated at 80 °C for 15 min. Due to the transparent asset of the glass substrate for the conventional method, MPs were observed using an inverted microscope (Zeiss AXIO equipped with an HBO 100 laser source) to confirm the efficiency of the staining process. In contrast, the stained MPs were placed on a MicroMetaSense, and the reflected fluorescent light from the MPs on this plasmonic metasurface was examined using an upright microscope (Axiovision Zeiss Scope-A1 microscope, Zeiss Colibri 7 LED light source, and Zeiss Filter Set 20) at various magnifications (5×, 20×, 40×, and 100×). In addition, the size variations of PMMA MPs were particularly examined to determine the detection ranges of wide-field light and fluorescence microscopes. In this regard, five different concentrations of stained PMMA MPs on MicroMetaSense were evaluated, depending on the particle size.

### Fluorescence Image Analysis

The fluorescence image intensity was analyzed using NIH ImageJ software (NIH). Initially, ROIs were defined, with each ROI having fixed dimensions of 3 × 3 pixels. ROIs were selected from both the background (noise, N) and the areas emitting fluorescence from MPs (signal, S). The average unweighted intensity values of these ROIs were then calculated and used as indicators of the fluorescence intensity. For comparative analysis, intensity measurements were taken from both glass substrates (N_G_ for noise and S_G_ for signal) and MicroMetaSense (N_M_ for noise and S_M_ for signal). Five distinct ROIs were utilized for each category to ensure accuracy: background noise (N_M_ and N_G_) and fluorescence signal (S_G_ and S_M_).

## Supporting Information Available

AFM analysis of MicroMetaSense, TGA results of PMMA and PET MPs, 3D-printed microfluidic design with dimensions, ROI analyses of MPs on glass and metasurface, and a table comparing the LOD values of MicroMetaSense and other studies (PDF)
