# Supplementary material for: MicroMetaSense: Coupling Plasmonic Metasurfaces with Fluorescence for Enhanced Detection of Microplastics in Real Samples
Source: ACS Sens. 2024 Dec 27;10(2):725–40. doi: 10.1021/acssensors.4c02070 (PMC11877509; doi:10.1021/acssensors.4c02070)
Supplement: Supplementary file 1 — se4c02070_si_001.pdf [file se4c02070_si_001.pdf]

Supporting Information

MicroMetaSense: Coupling Plasmonic Metasurfaces with Fluorescence for Enhanced Detection of Microplastics in Real Samples

Emre Ece[1,2,#], Yusuf Aslan[1,2,#], Nedim Hacıosmanoğlu[1,2,#], Fatih Inci[1,2,*]

[1] UNAM-National Nanotechnology Research Center, Bilkent University, 06800, Ankara, Turkey

[2] Institute of Materials Science and Nanotechnology, Bilkent University, 06800, Ankara, Turkey

[#] Contributed equally

[*] Corresponding author: finci@bilkent.edu.tr

ORCID ID: 0000-0002-9918-5038

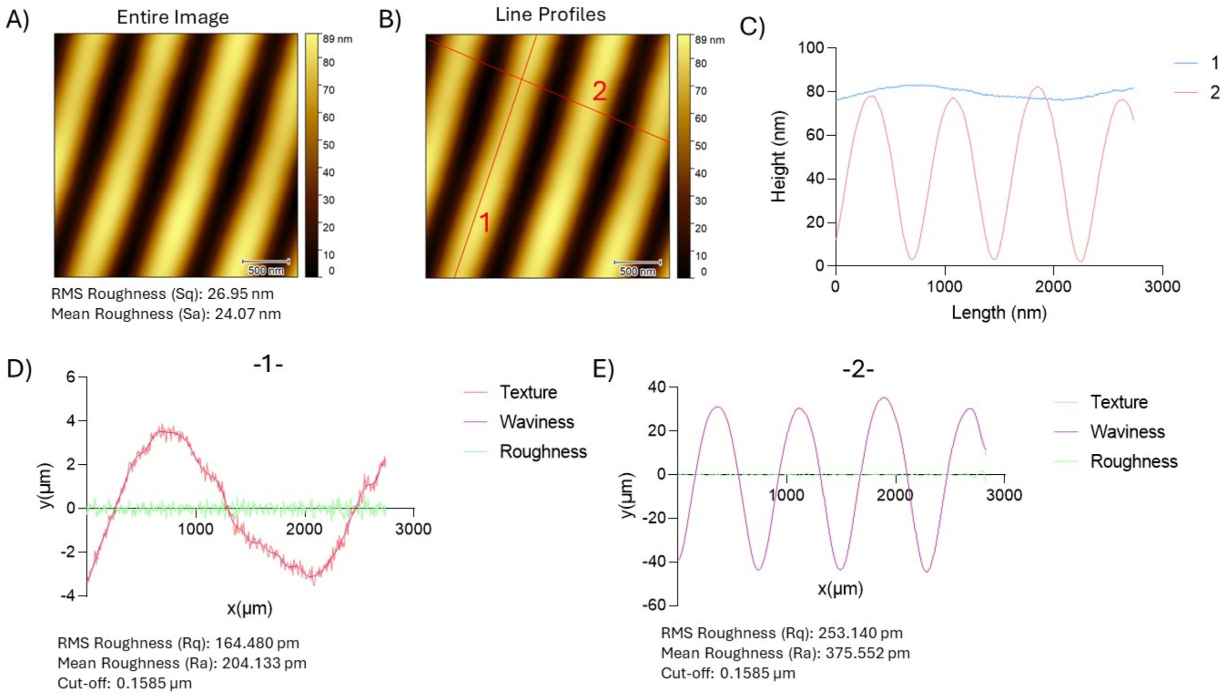

Supplementary Figure 1. Surface roughness analysis of MicroMetaSense. (A) The root mean square (RMS) roughness (Sq) and mean roughness (Sa) values are calculated for the entire AFM image of MicroMetaSense. (B) Line profiles are taken both along (1) and across (2) the grating structure. (C) Height profiles are plotted as a function of distance for both line profiles (1 and 2). (D) Texture, waviness, and roughness plots are shown for the line profile along (1) the grating structure, using a cut-off value of 0.1585 µm. (E) Similarly, texture, waviness, and roughness plots are shown for the line profile across (2) the grating structure, with the same cut-off value of 0.1585 µm. RMS roughness and mean roughness values for both line profiles are presented below the respective figures.

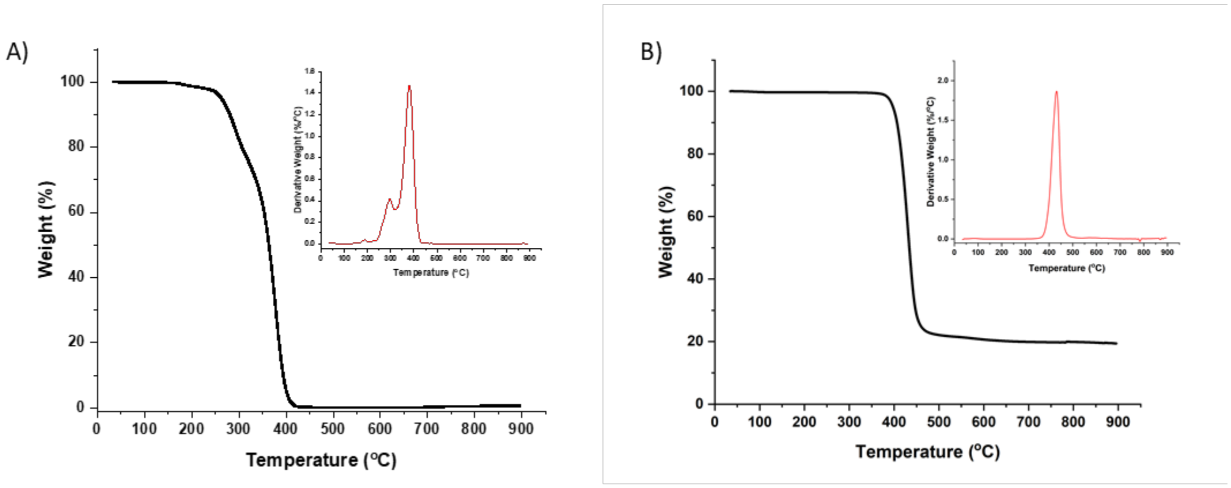

Supplementary Figure 2. Thermogravimetric analysis (TGA) of A) PMMA and B) PET MPs with their derivative weight (%/°C) results.

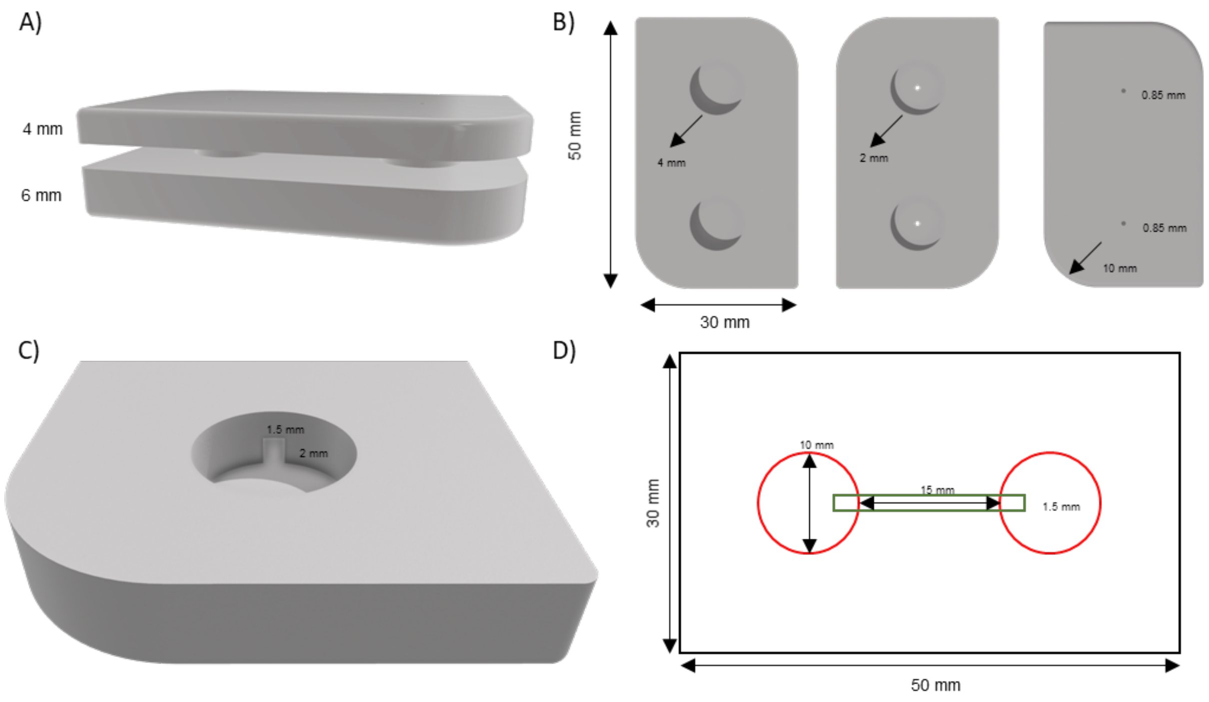

Supplementary Figure 3. Dimensions and 3D model illustration of microfluidic ultrafiltration chip. A) Upper (4 mm) and below (6 mm) part of the chip, B) dimensions for below (left) and upper (middle, from bottom) and left (from top) part, C) channel dimensions, D) 2D dimensions for DSA cutting.

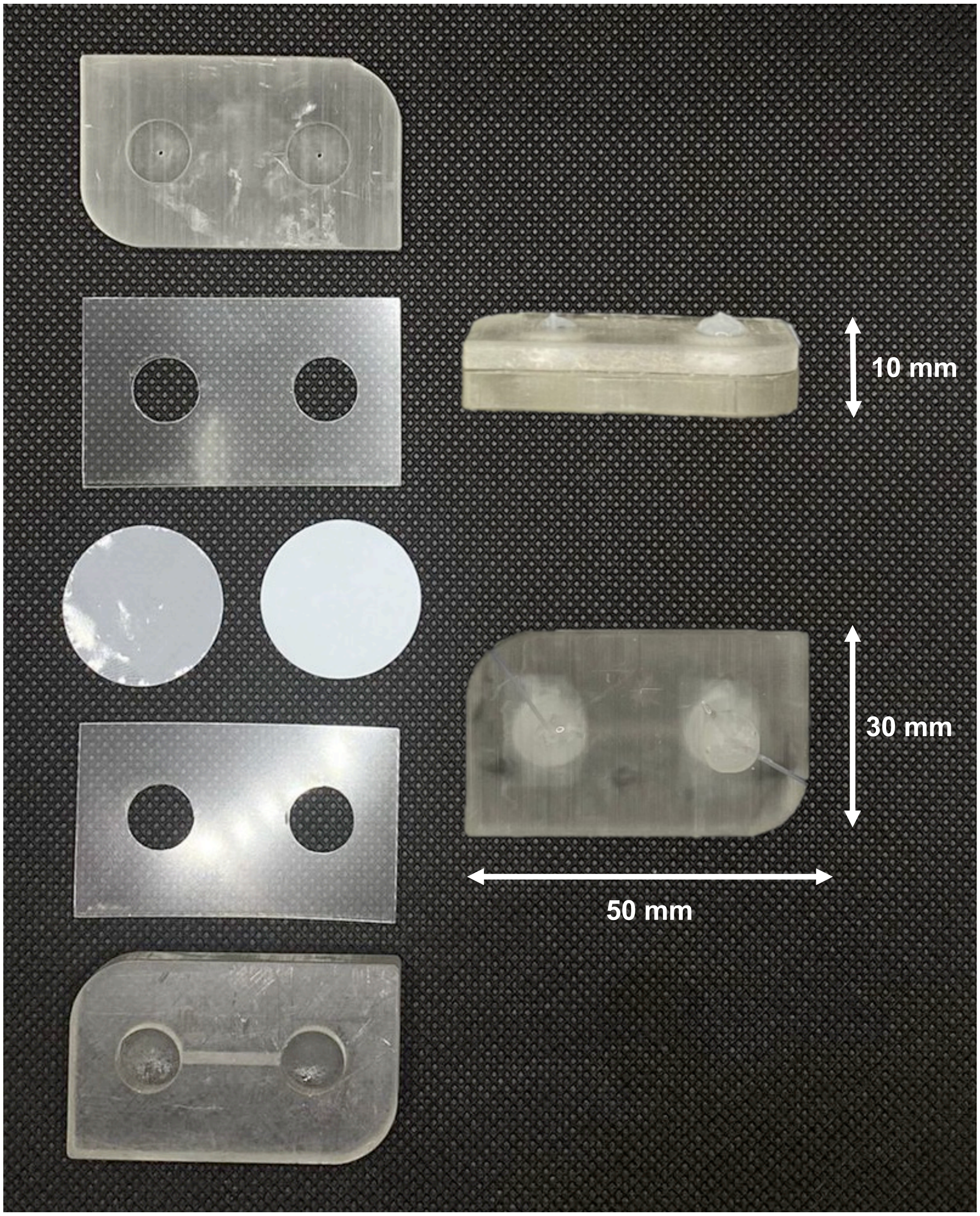

Supplementary Figure 4. Parts of microfluidic ultrafiltration chip, left side includes upper part, DSA, filters, DSA, and bottom part in order. Right side includes integrated chip from side and top view. 1 TL coin used for size comparison.

A)

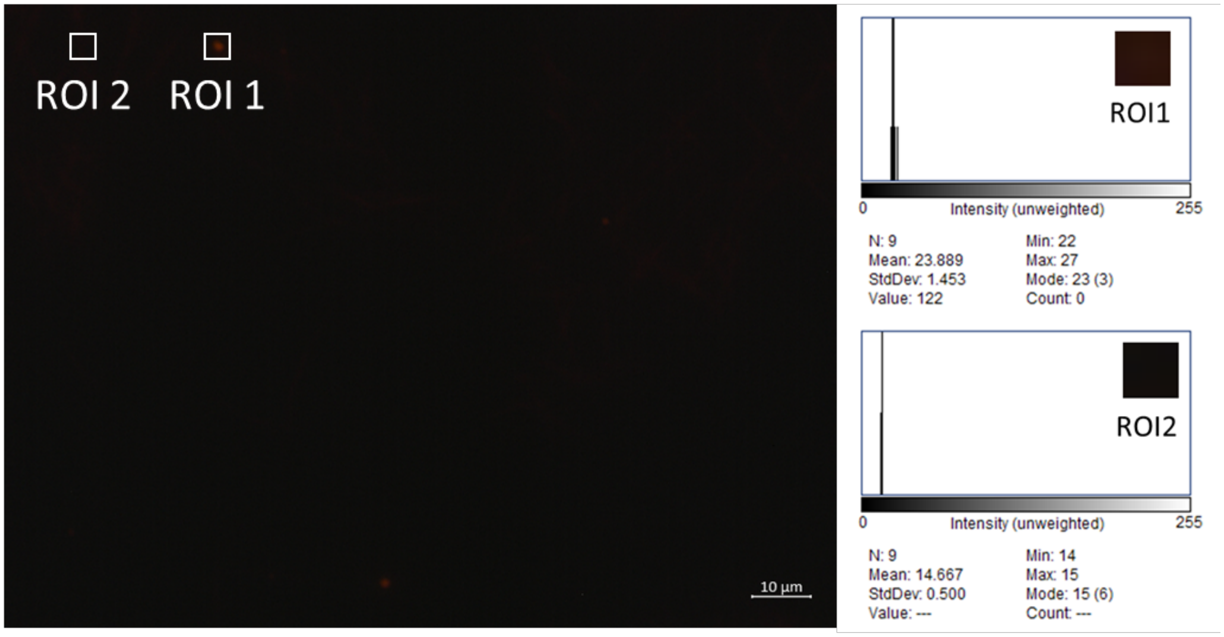

B)

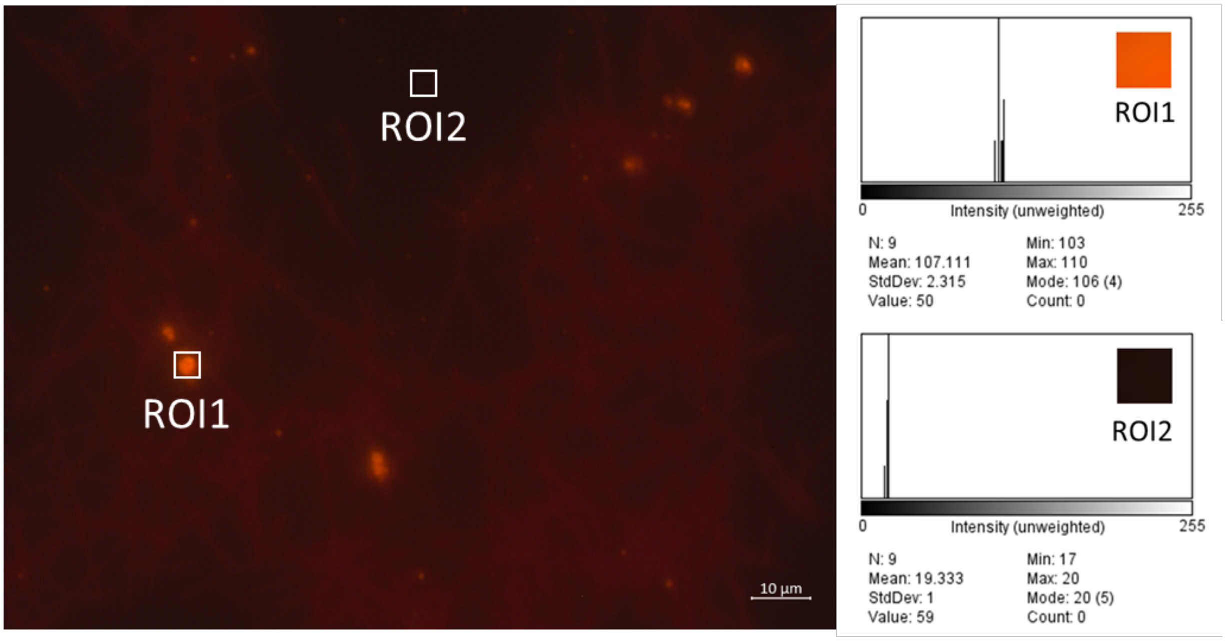

C)

$$EF = \frac{S_M/N_M}{S_G/N_G} \qquad EF = \frac{(107.111)/(19.333)}{(23.889)/(14.667)}$$

EF1 for PMMA MP: 3.40

Supplementary Figure 5. Determination of EF1 for PMMA MPs on a) glass and b) metasurface using noise (ROI 2, NG and NM) and signals (ROI 1, SG and SM) by c) calculating signal noise ratios.

A)

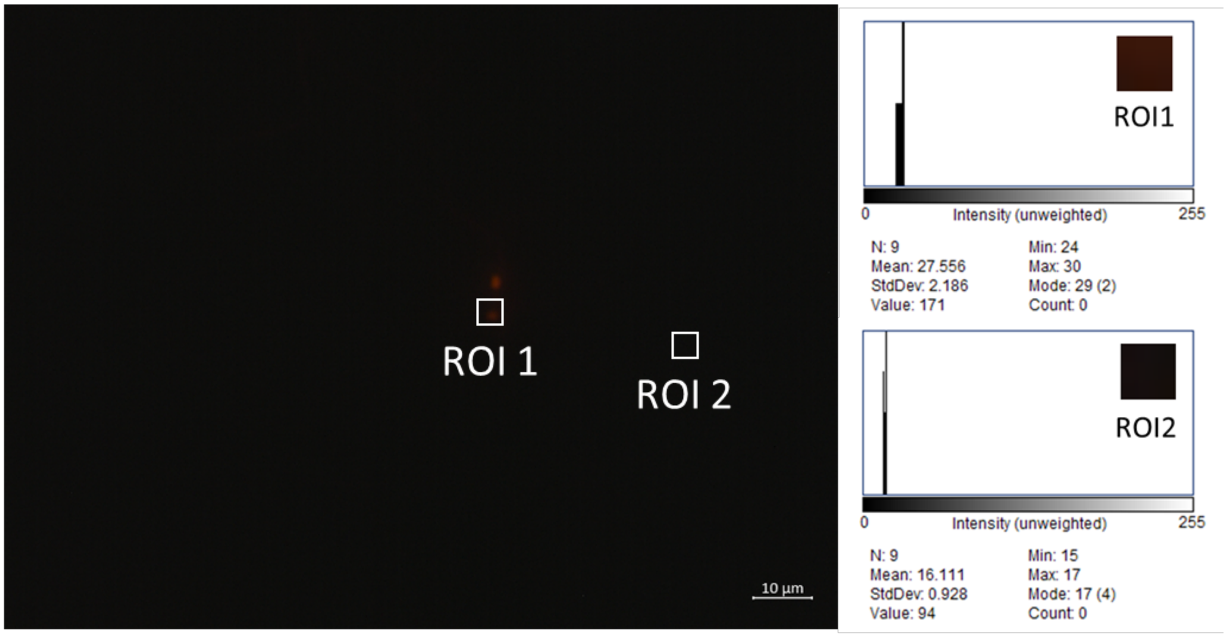

B)

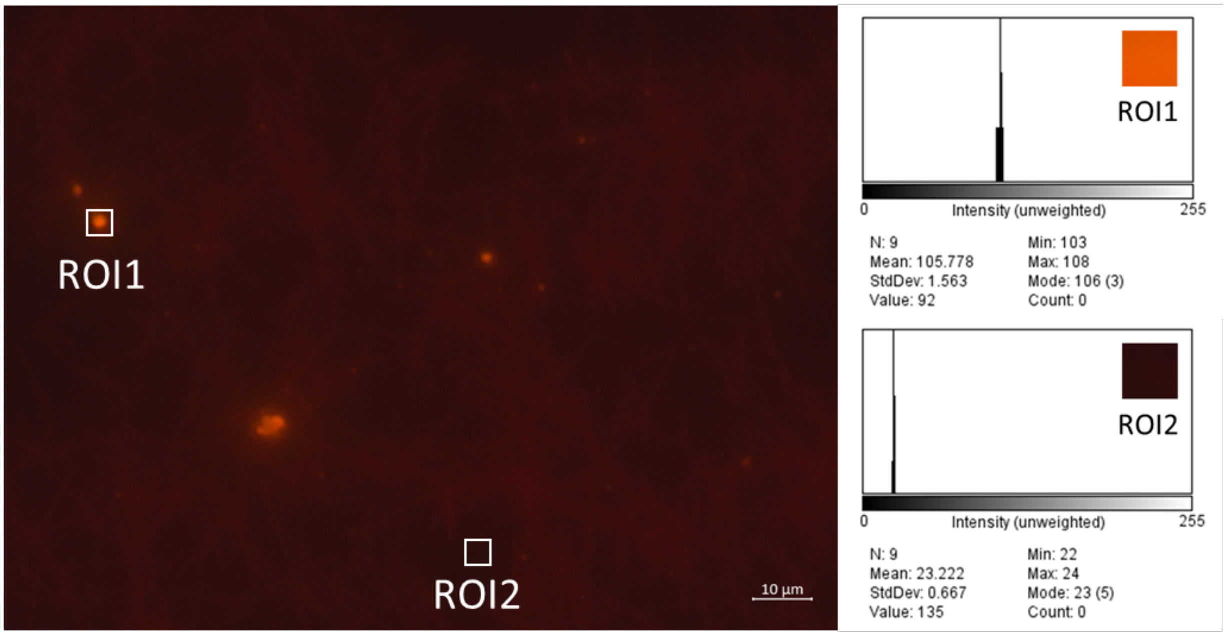

C)

$$EF = \frac{S_M/N_M}{S_G/N_G} \qquad EF = \frac{(105.778)/(23.222)}{(27.556)/(16.111)}$$

EF2 for PMMA MP: 2.65

Supplementary Figure 6. Determination of EF2 for PMMA MPs on a) glass and b) metasurface using noise (ROI 2, NG and NM) and signals (ROI 1, SG and SM) by c) calculating signal noise ratios.

A)

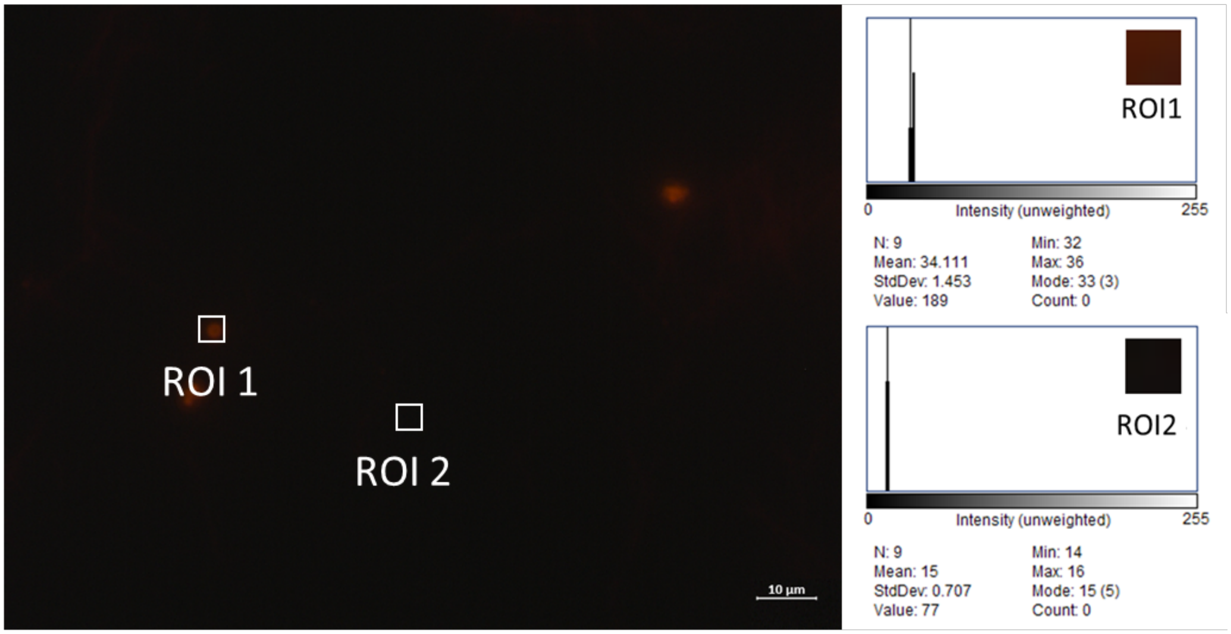

B)

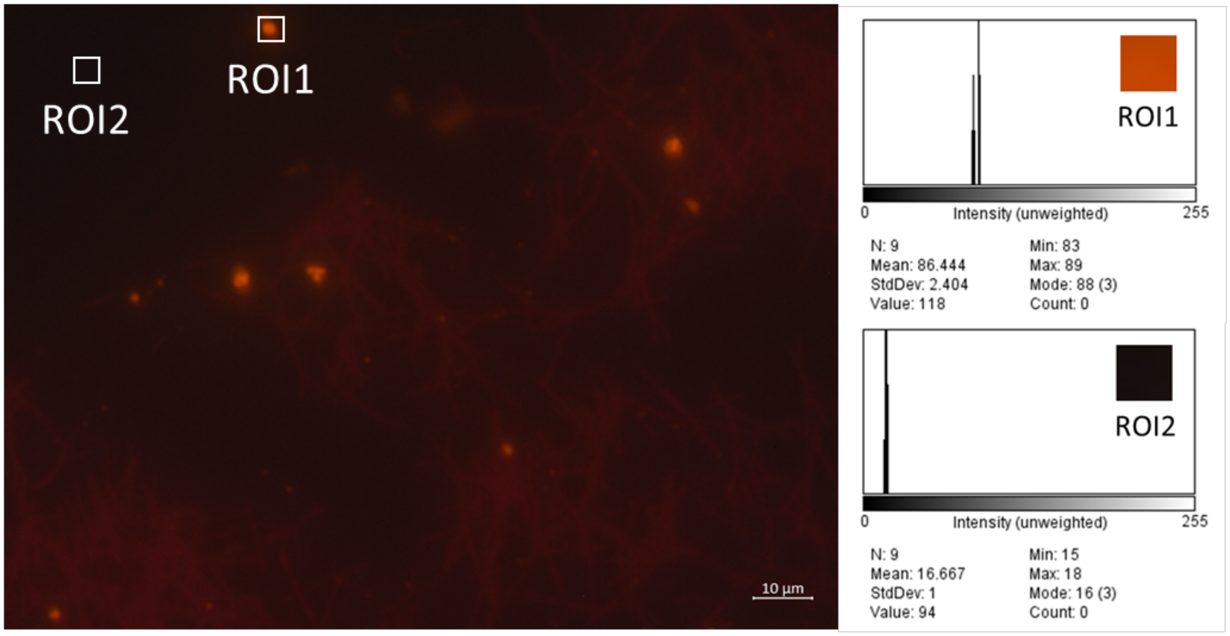

C)

$$EF = \frac{S_M/N_M}{S_G/N_G} \qquad EF = \frac{(86.444)/(16.667)}{(34.111)/(15)}$$

EF3 for PMMA MP: 2.28

Supplementary Figure 7. Determination of EF3 for PMMA MPs on a) glass and b) metasurface using noise (ROI 2, NG and NM) and signals (ROI 1, SG and SM) by c) calculating signal noise ratios.

A)

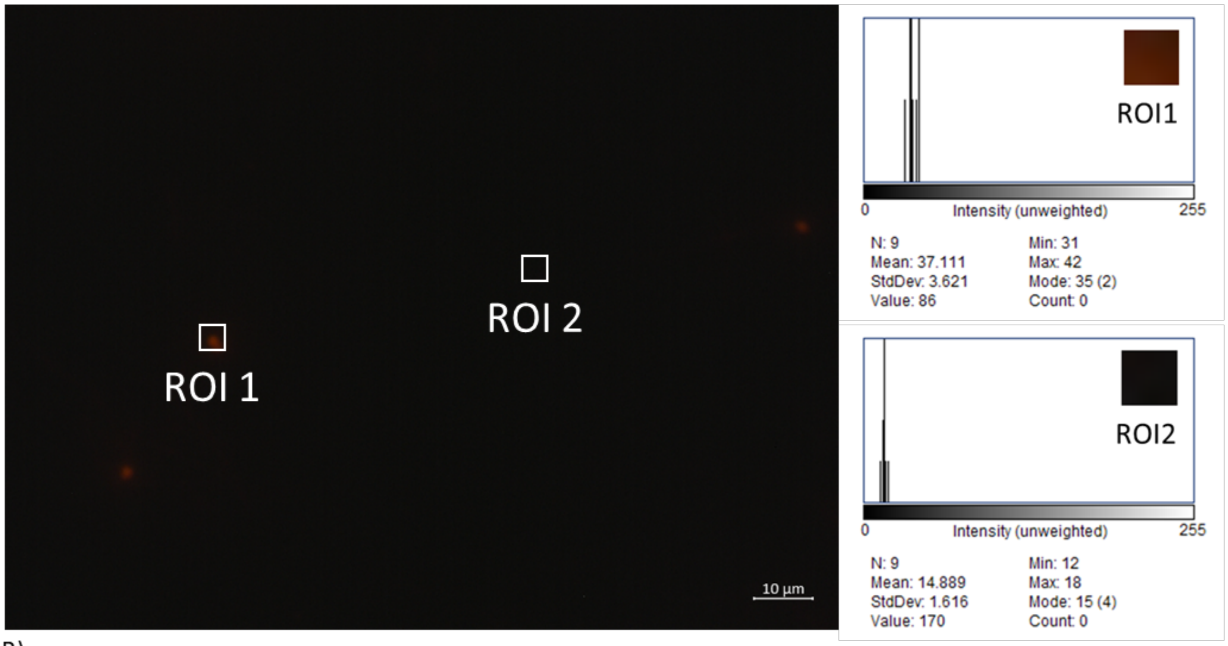

B)

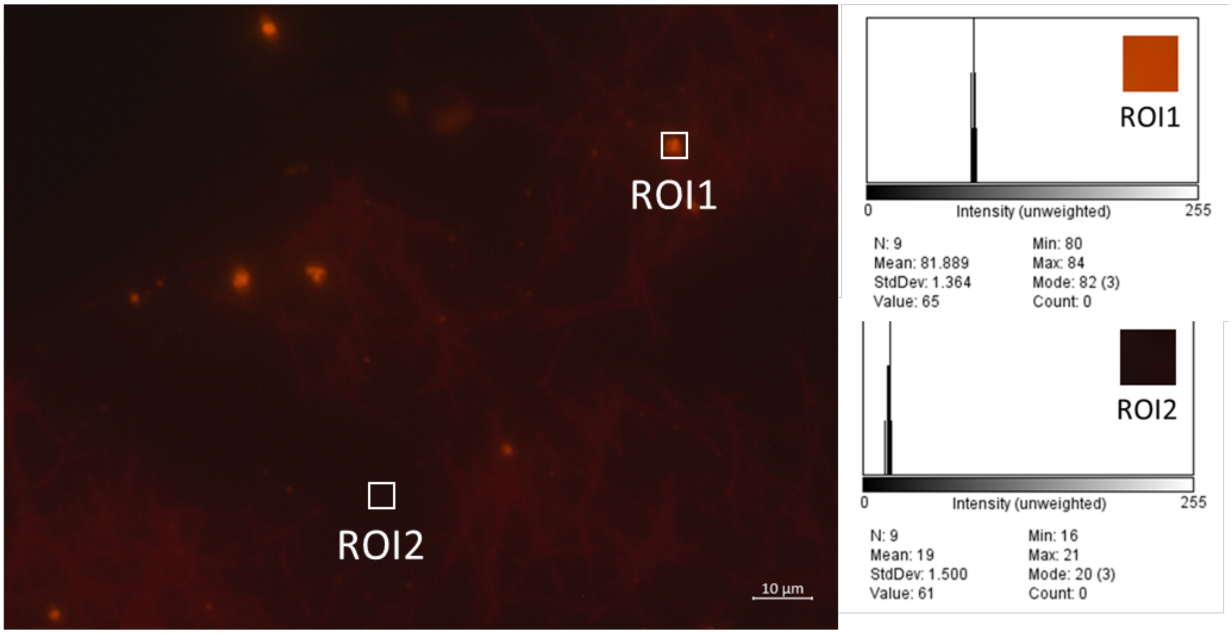

C)

$$EF = \frac{S_M/N_M}{S_G/N_G} \qquad EF = \frac{(81.889)/(19)}{(37.111)/(14.889)}$$

EF4 for PMMA MP: 1.73

Supplementary Figure 8. Determination of EF4 for PMMA MPs on a) glass and b) metasurface using noise (ROI 2, NG and NM) and signals (ROI 1, SG and SM) by c) calculating signal noise ratios.

A)

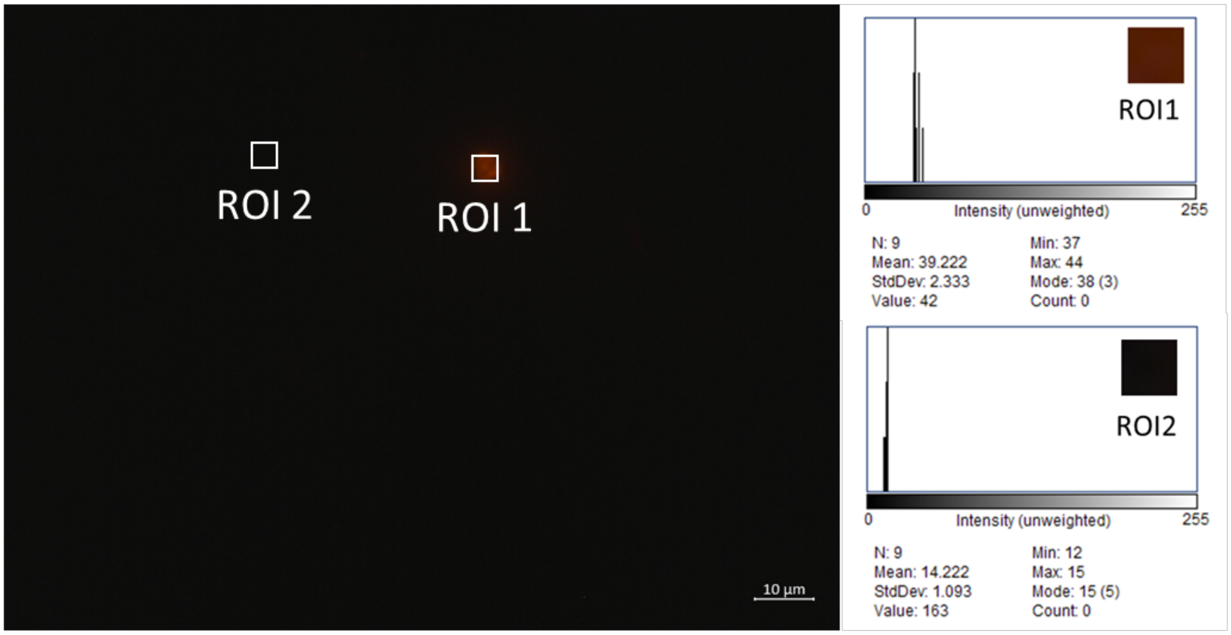

B)

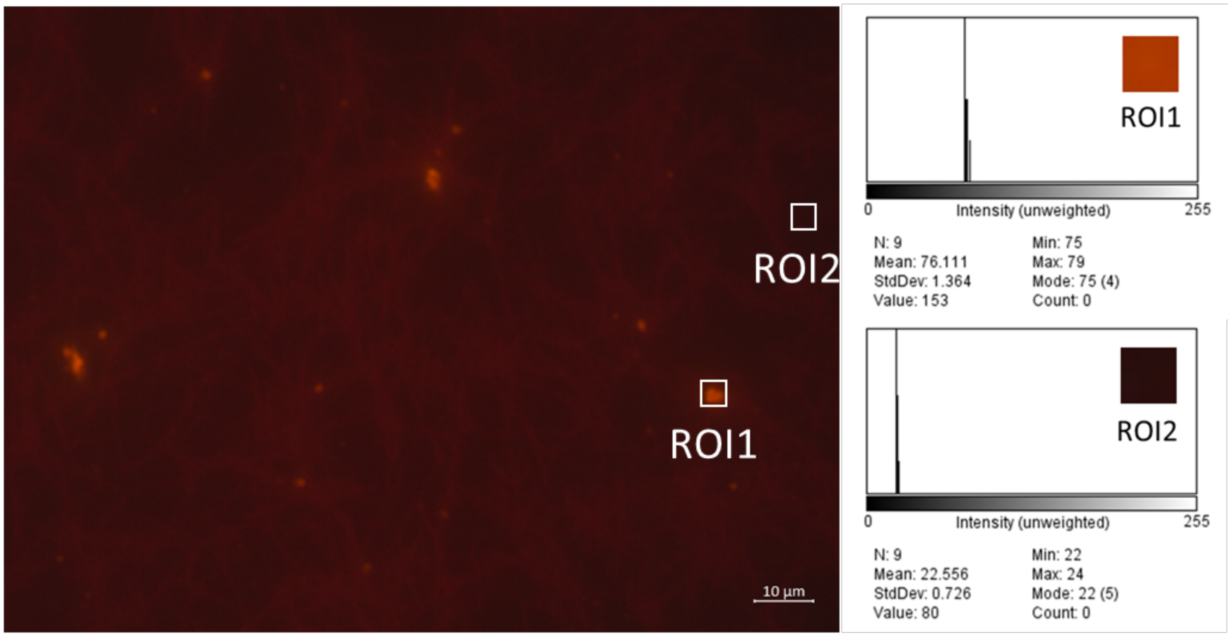

C)

$$EF = \frac{S_M/N_M}{S_G/N_G} \qquad EF = \frac{(76.111)/(22.556)}{(39.222)/(14.222)}$$

EF5 for PMMA MP: 1.22

Supplementary Figure 9. Determination of EF5 for PMMA MPs on a) glass and b) metasurface using noise (ROI 2, NG and NM) and signals (ROI 1, SG and SM) by c) calculating signal noise ratios.

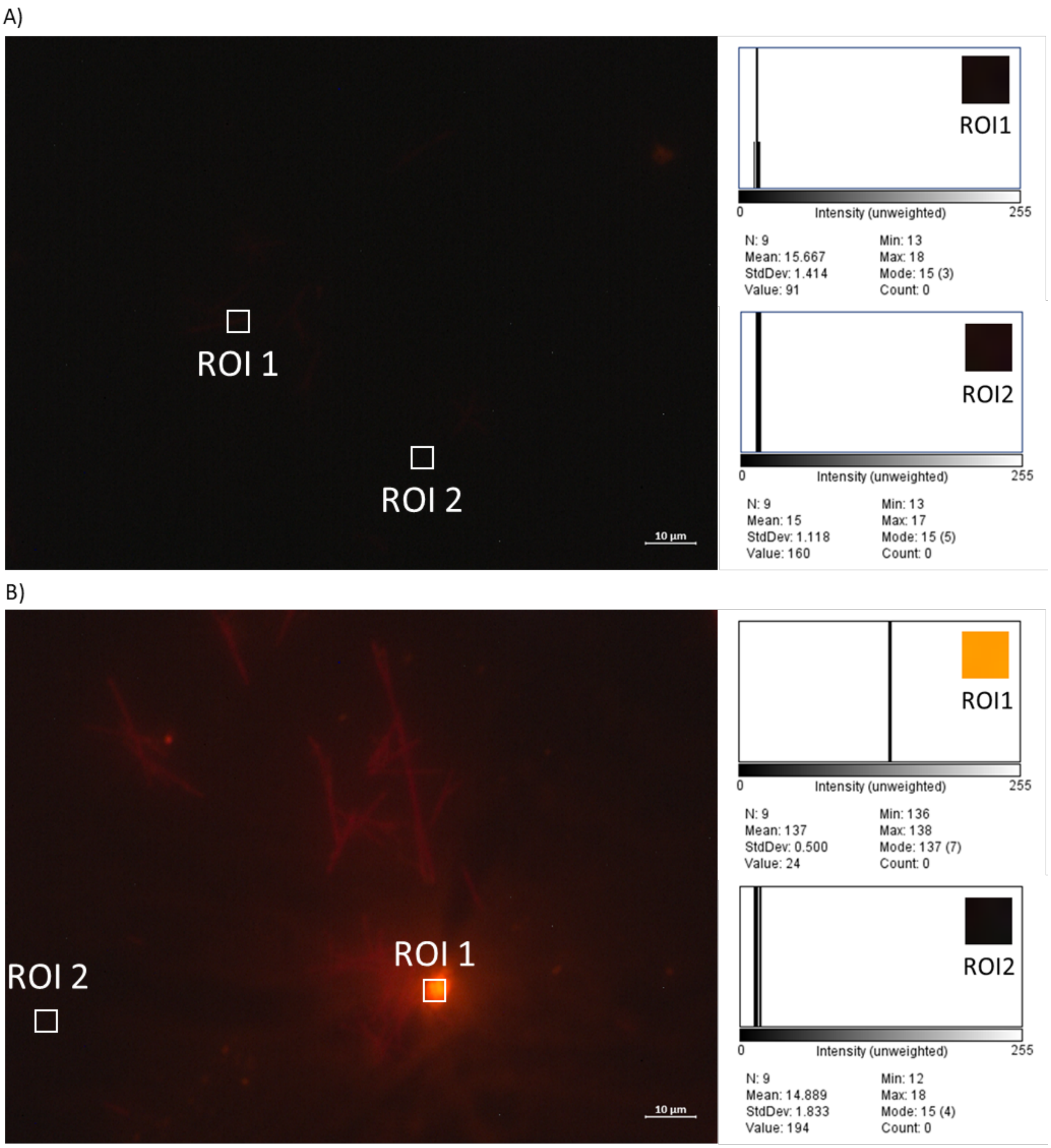

C)

$$EF = \frac{S_M/N_M}{S_G/N_G} \qquad EF = \frac{(137)/(14.889)}{(15.667)/(15)}$$

EF1 for PET MP: 8.79

Supplementary Figure 10. Determination of EF1 for PET MPs on a) glass and b) metasurface using noise (ROI 2, NG and NM) and signals (ROI 1, SG and SM) by c) calculating signal noise ratios.

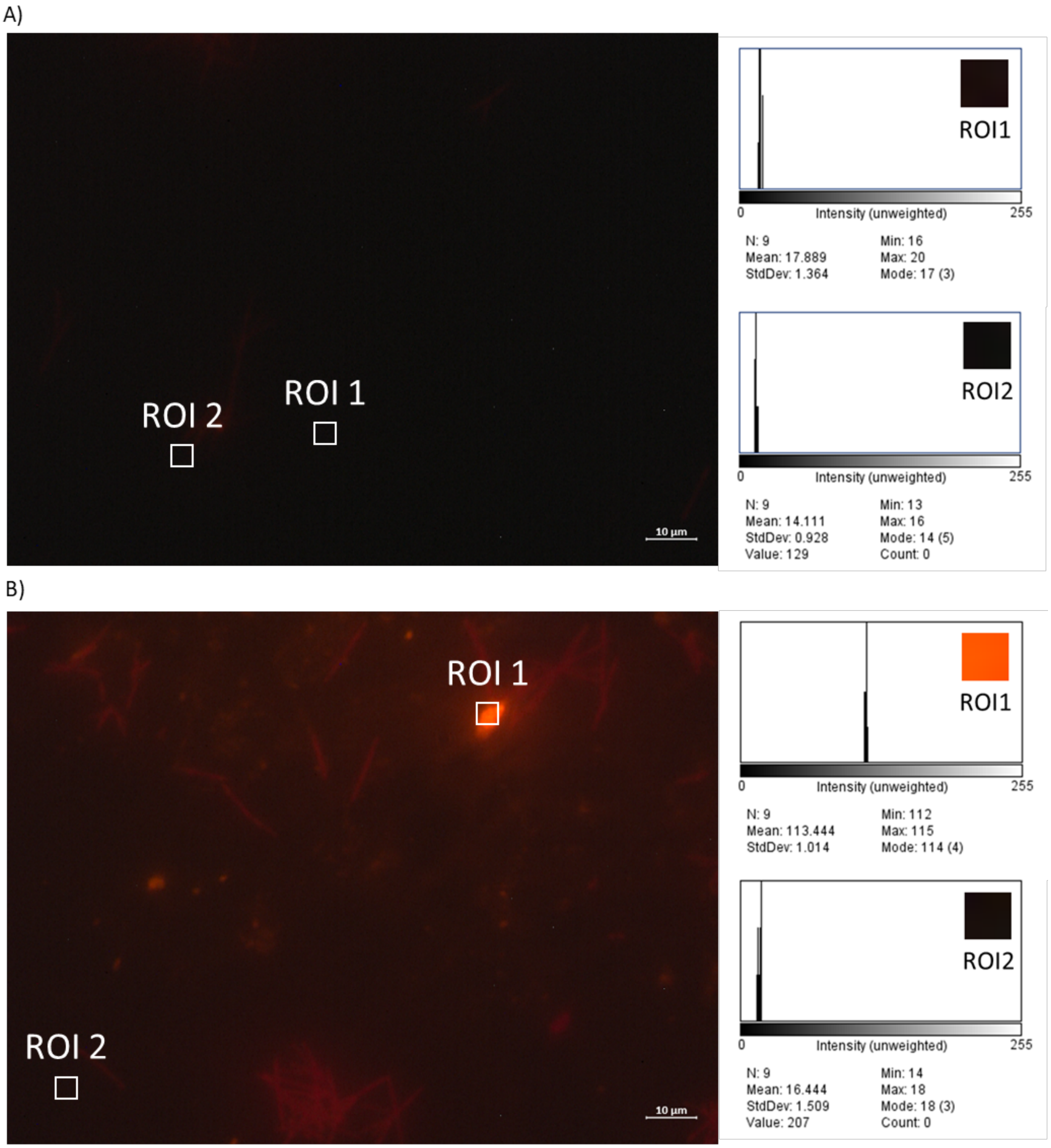

C)

$$EF = \frac{S_M/N_M}{S_G/N_G} \qquad EF = \frac{(113.444)/(16.444)}{(17.889)/(14.111)}$$

EF2 for PET MP: 5.94

Supplementary Figure 11. Determination of EF2 for PET MPs on a) glass and b) metasurface using noise (ROI 2, NG and NM) and signals (ROI 1, SG and SM) by c) calculating signal noise ratios.

A)

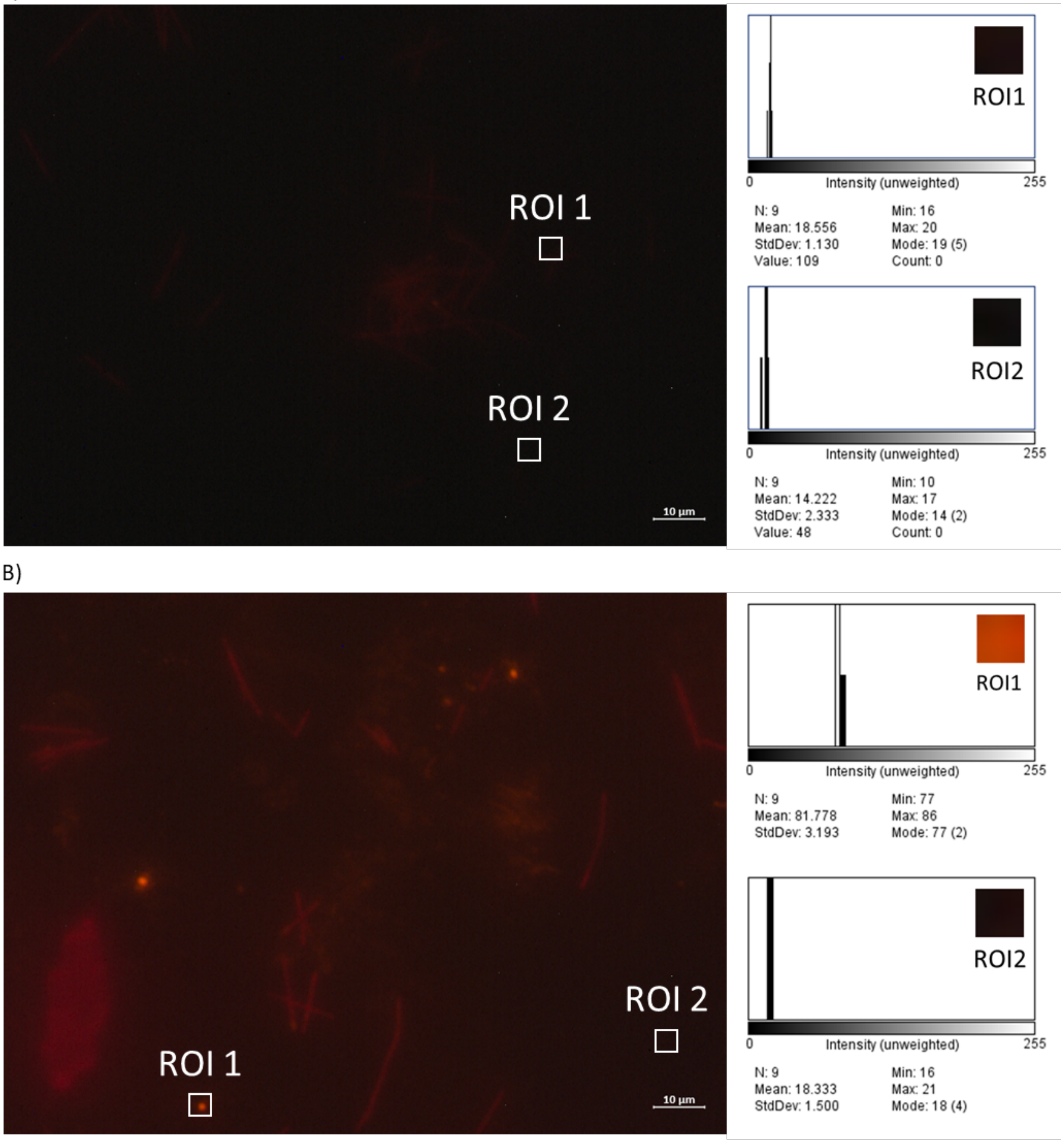

C)

$$EF = \frac{S_M/N_M}{S_G/N_G} \qquad EF = \frac{(81.778)/(18.333)}{(18.556)/(14.222)}$$

EF3 for PET MP: 3.40

Supplementary Figure 12. Determination of EF3 for PET MPs on a) glass and b) metasurface using noise (ROI 2, NG and NM) and signals (ROI 1, SG and SM) by c) calculating signal noise ratios.

A)

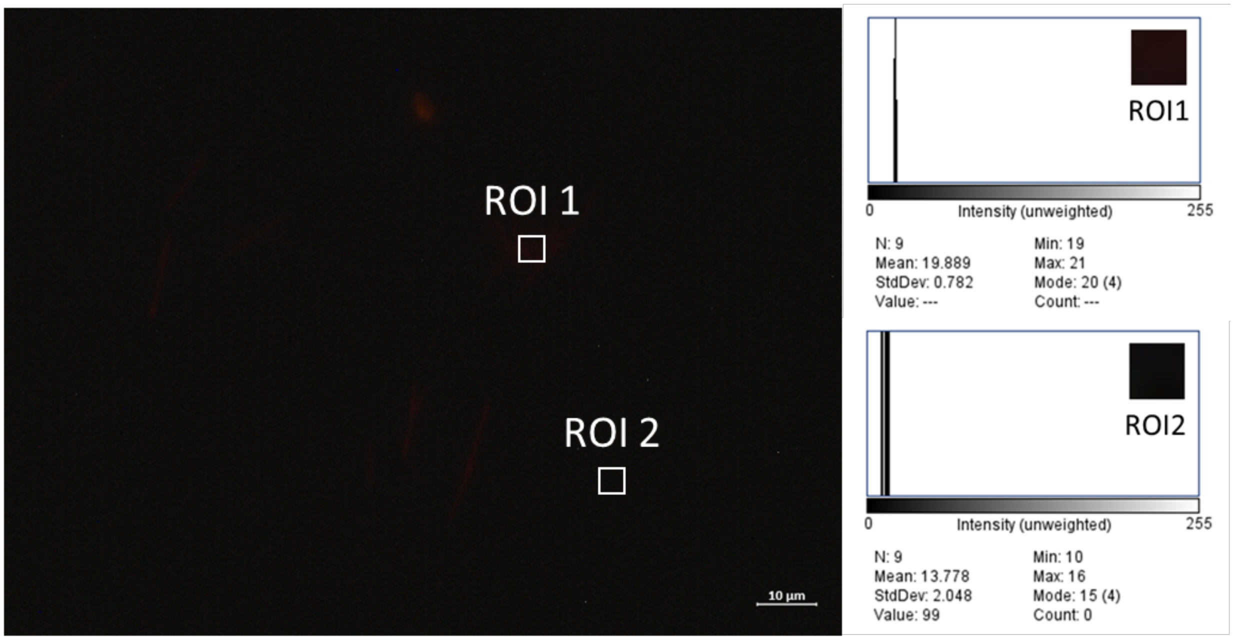

B)

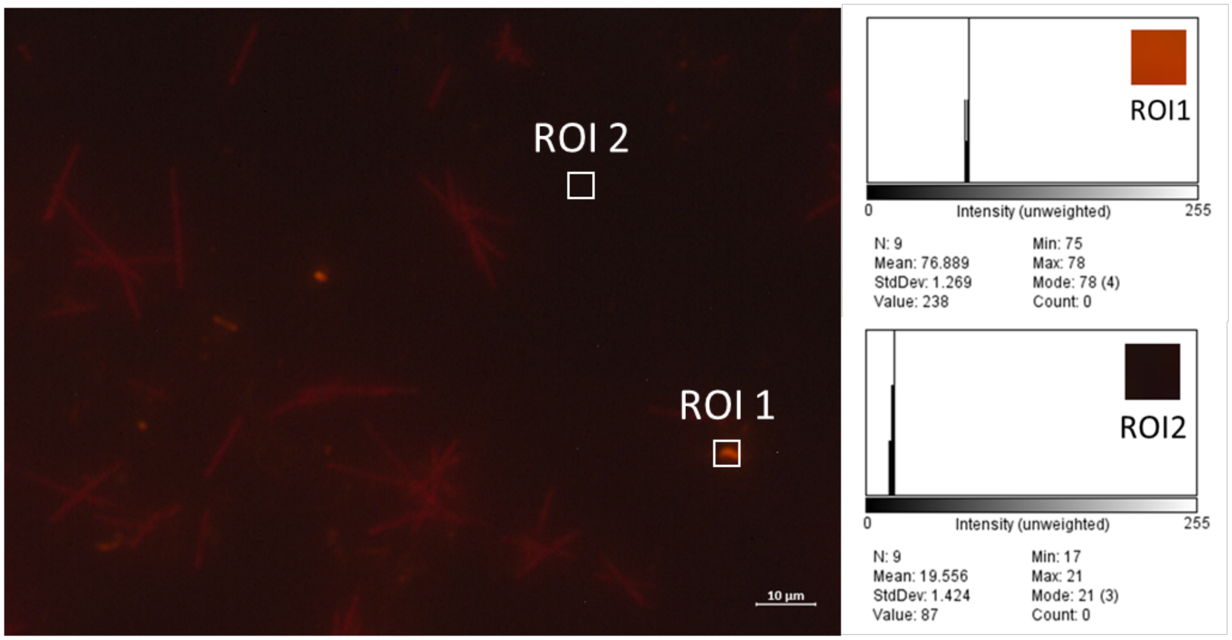

C)

$$EF = \frac{S_M/N_M}{S_G/N_G}$$

$$EF = \frac{(76.889)/(19.556)}{(19.889)/(13.778)}$$

EF4 for PET MP: 2.78

Supplementary Figure 13. Determination of EF4 for PET MPs on a) glass and b) metasurface using noise (ROI 2, NG and NM) and signals (ROI 1, SG and SM) by c) calculating signal noise ratios.

A)

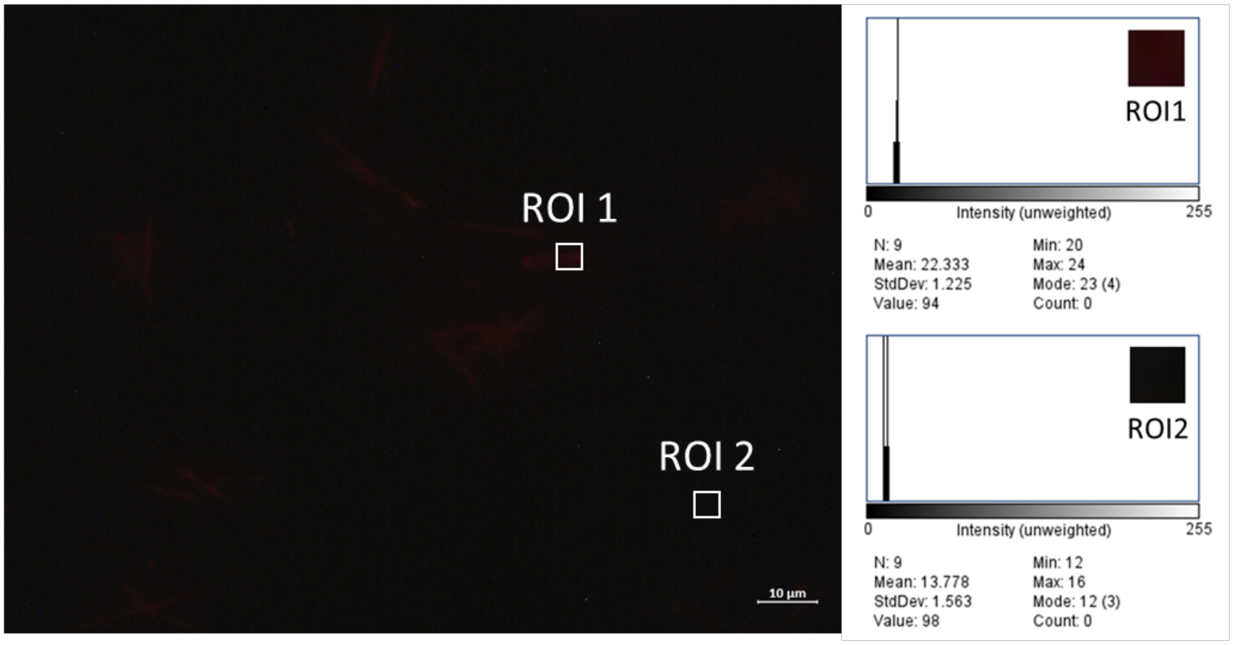

B)

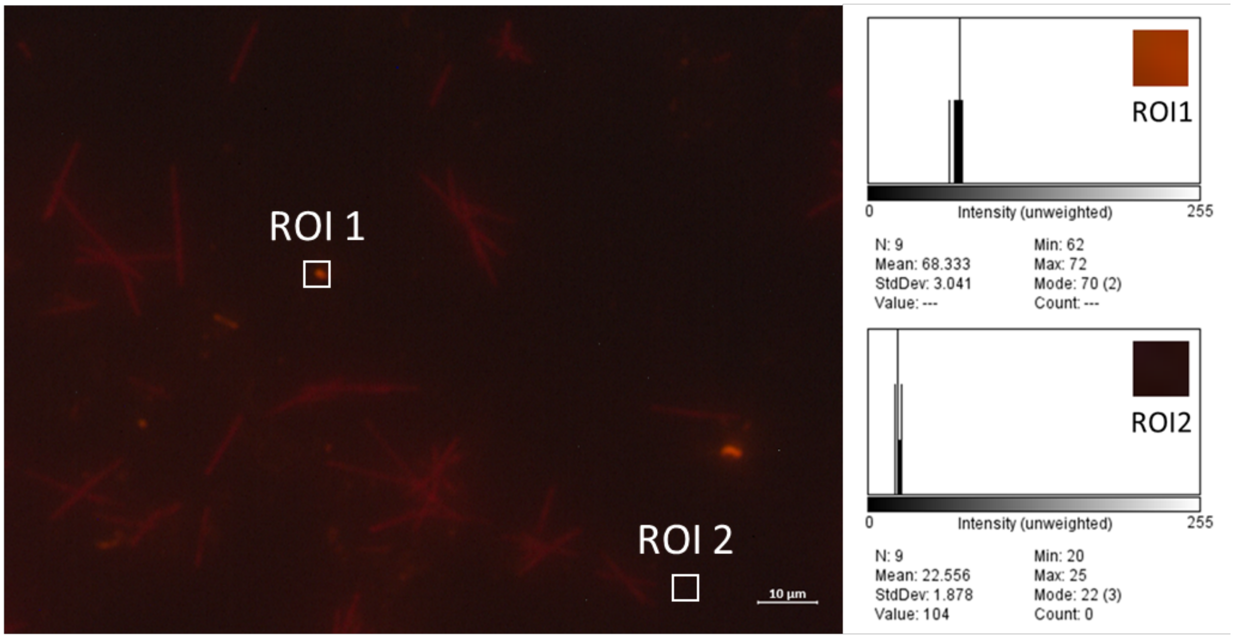

C)

$$EF = \frac{S_M/N_M}{S_G/N_G} \qquad EF = \frac{(68.333)/(22.556)}{(22.333)/(13.778)}$$

EF5 for PET MP: 1.87

Supplementary Figure 14. Determination of EF5 for PET MPs on a) glass and b) metasurface using noise (ROI 2, NG and NM) and signals (ROI 1, SG and SM) by c) calculating signal noise ratios.

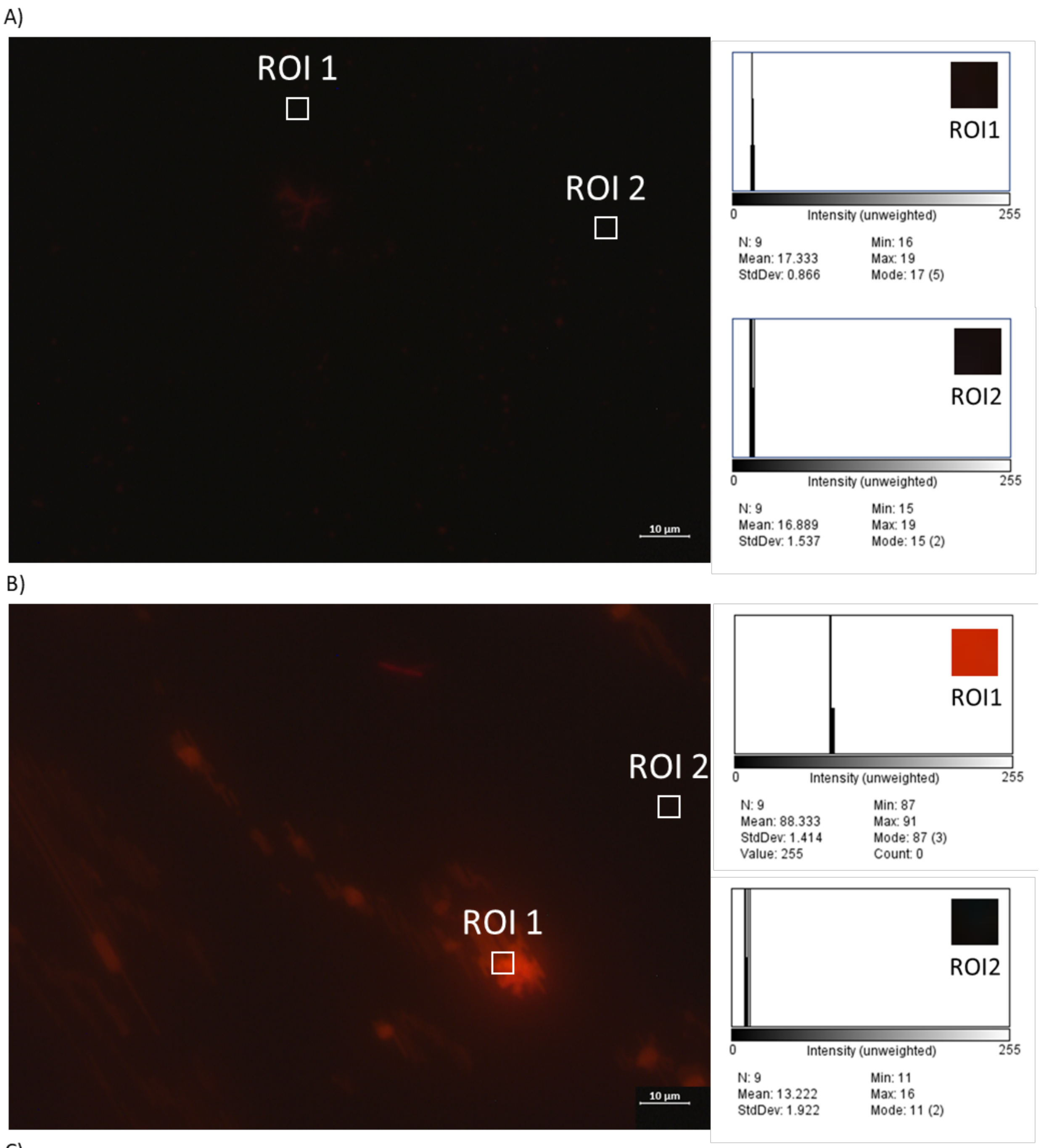

C)

$$EF = \frac{S_M/N_M}{S_G/N_G} \qquad EF = \frac{(88.333)/(13.222)}{(17.333)/(16.889)}$$

EF1 for MP in Tap: 6.52

Supplementary Figure 15. Determination of EF1 for MP in tap water on a) glass and b) metasurface using noise (ROI 2, NG and NM) and signals (ROI 1, SG and SM) by c) calculating signal noise ratios.

A)

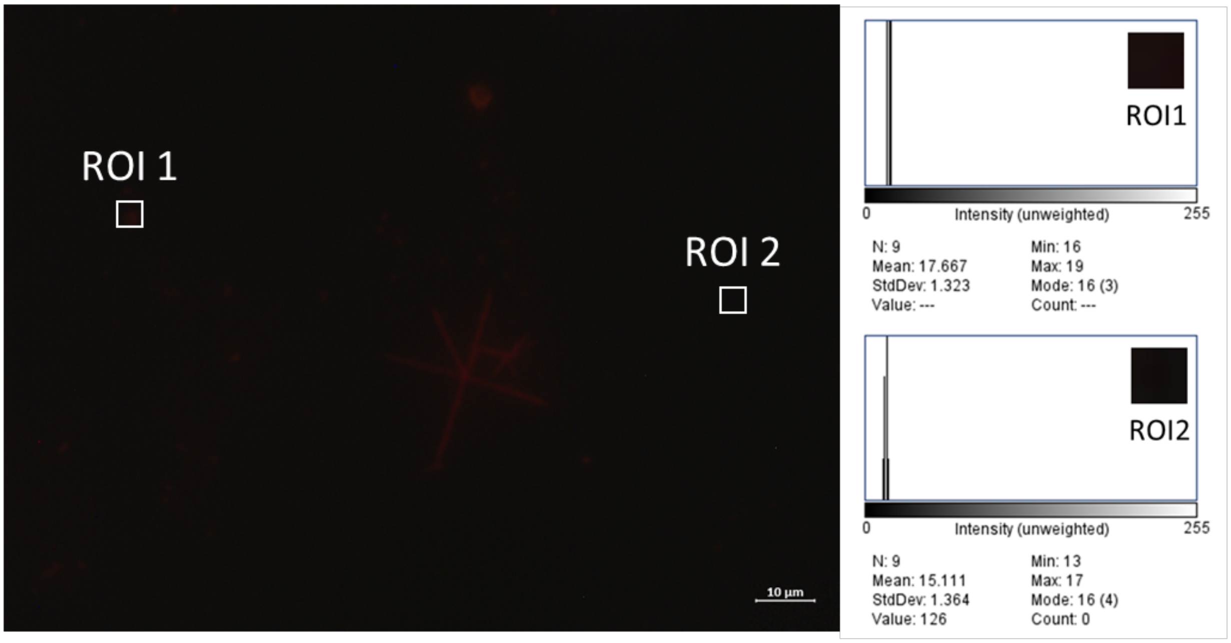

B)

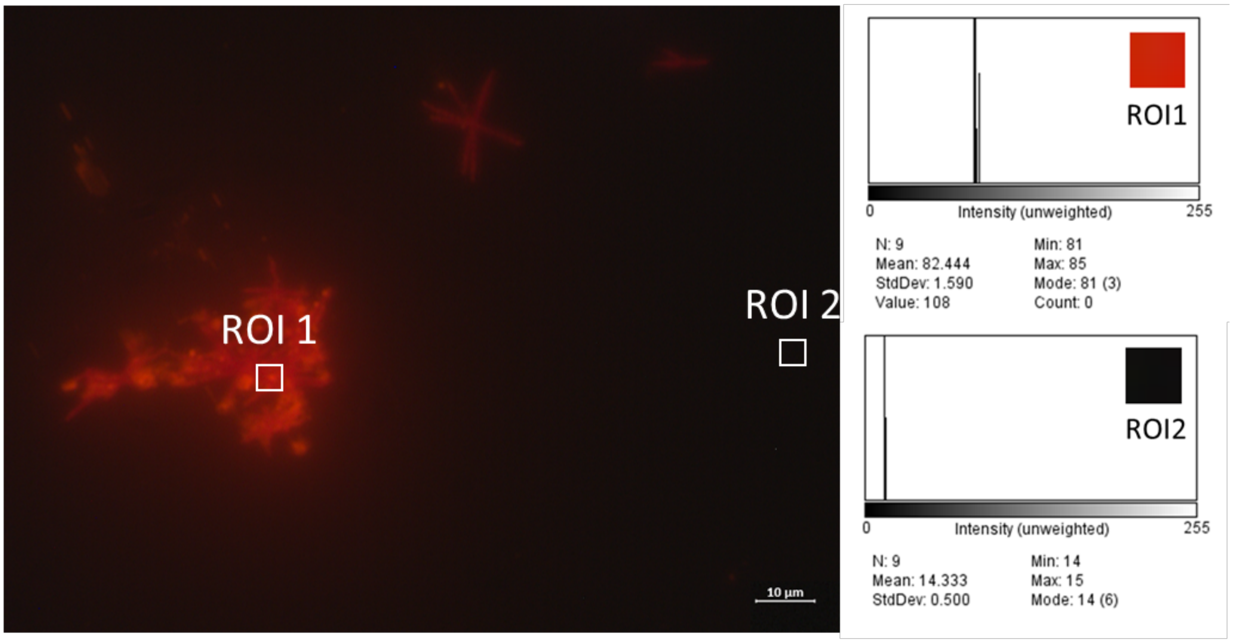

C)

$$EF = \frac{S_M/N_M}{S_G/N_G} \qquad EF = \frac{(82.444)/(14.333)}{(17.667)/(15.111)}$$

EF2 for MP in Tap: 4.90

Supplementary Figure 16. Determination of EF2 for MP in tap water on a) glass and b) metasurface using noise (ROI 2, NG and NM) and signals (ROI 1, SG and SM) by c) calculating signal noise ratios.

A)

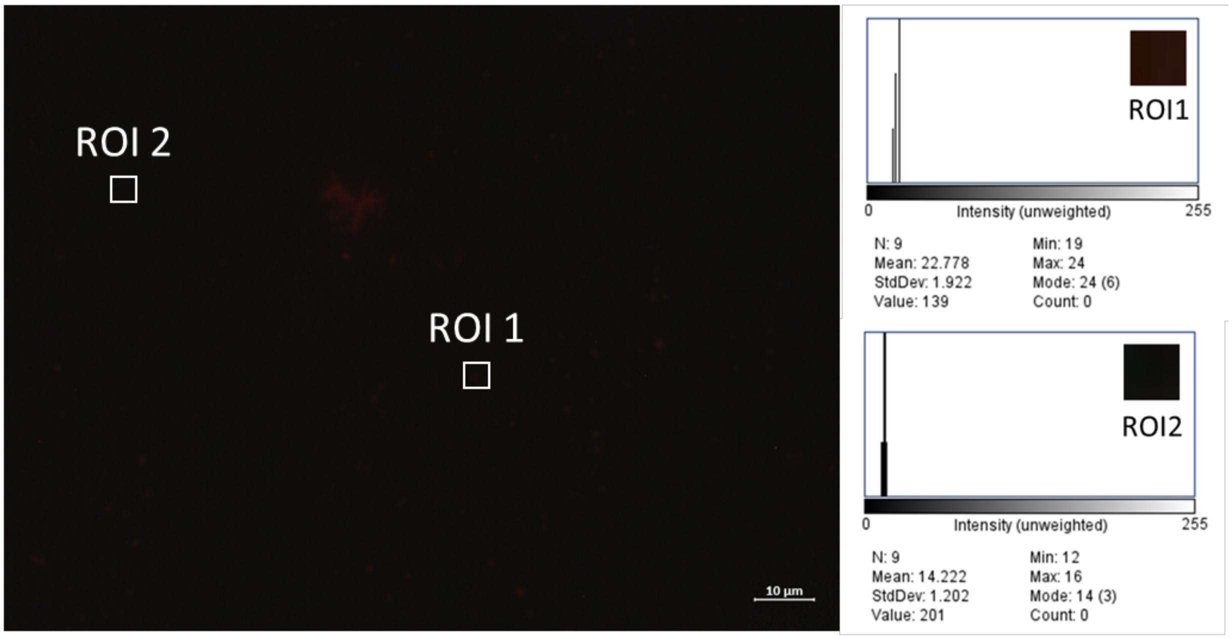

B)

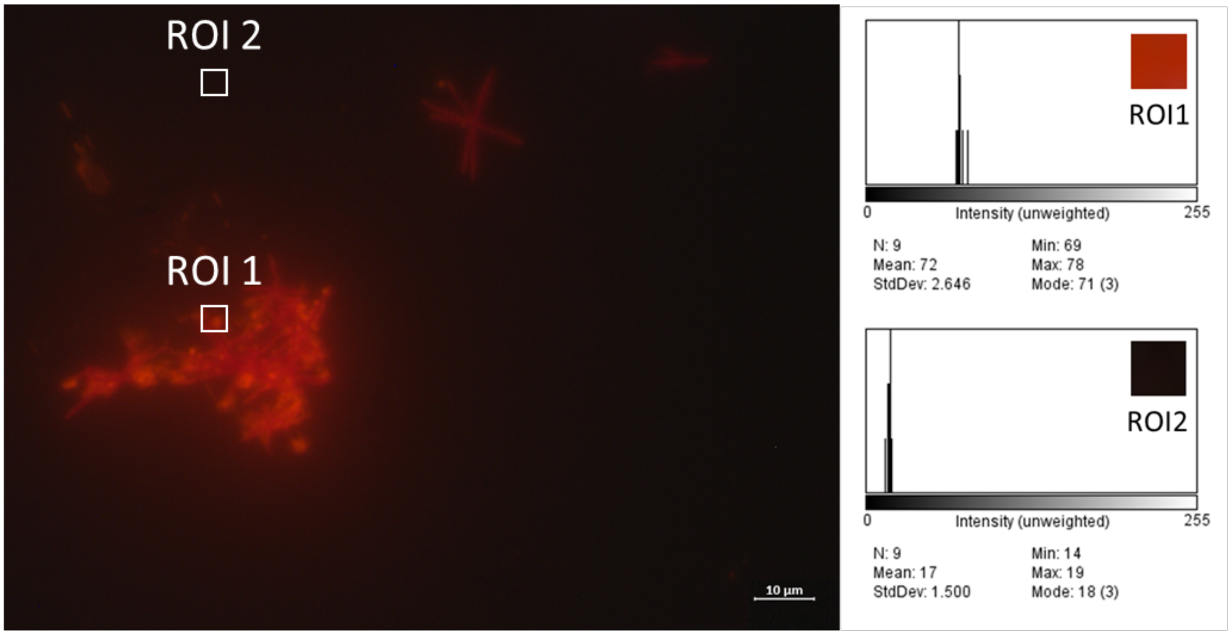

C)

$$EF = \frac{S_M/N_M}{S_G/N_G} \qquad EF = \frac{(72)/(17)}{(22.778)/(14.222)} \qquad \text{EF3 for MP in Tap: 2.63}$$

Supplementary Figure 17. Determination of EF3 for MP in tap water on a) glass and b) metasurface using noise (ROI 2, NG and NM) and signals (ROI 1, SG and SM) by c) calculating signal noise ratios.

A)

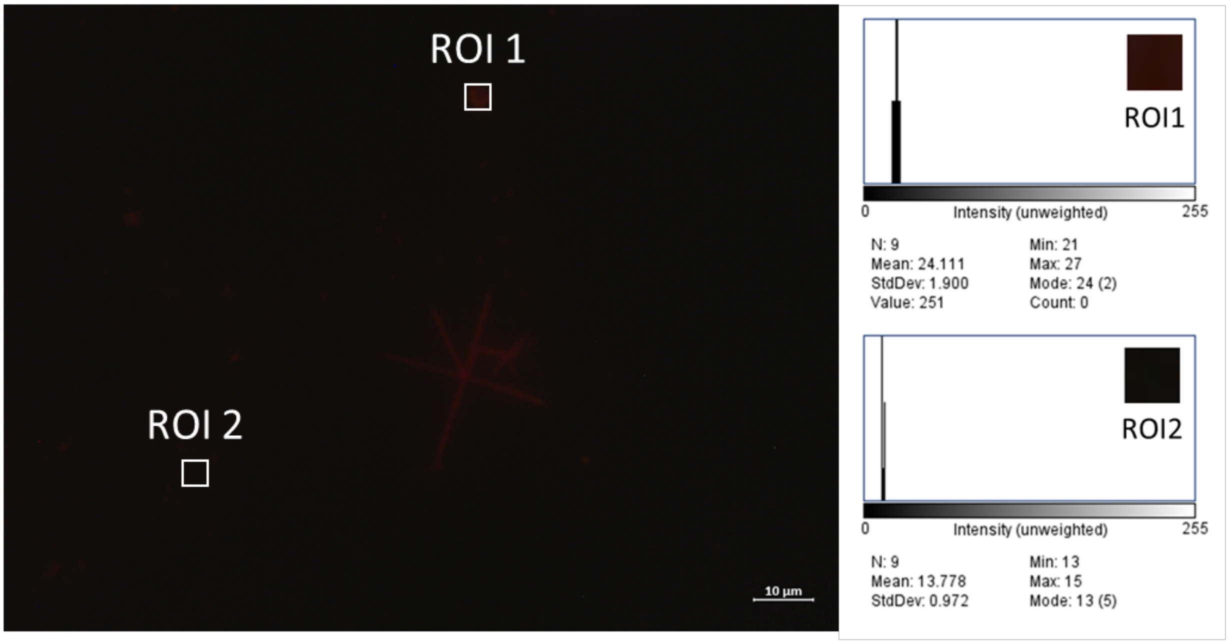

B)

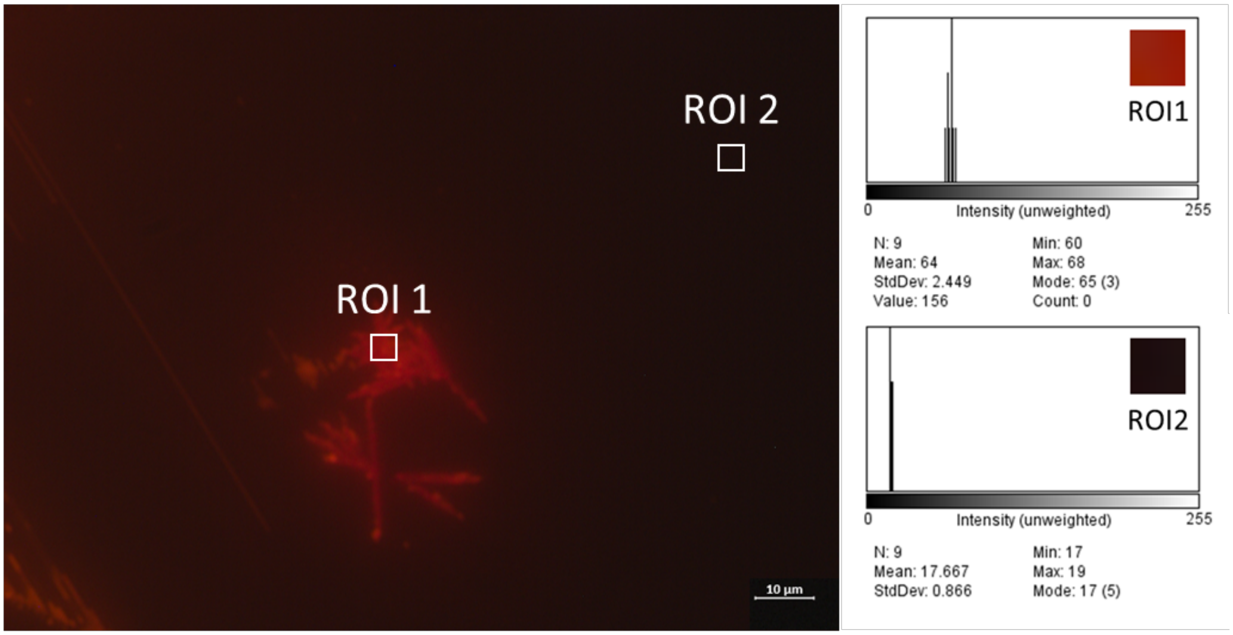

C)

$$EF = \frac{S_M/N_M}{S_G/N_G} \qquad EF = \frac{(64)/(17.667)}{(24.111)/(13.778)}$$

EF4 for MP in Tap: 2.06

Supplementary Figure 18. Determination of EF4 for MP in tap water on a) glass and b) metasurface using noise (ROI 2, NG and NM) and signals (ROI 1, SG and SM) by c) calculating signal noise ratios.

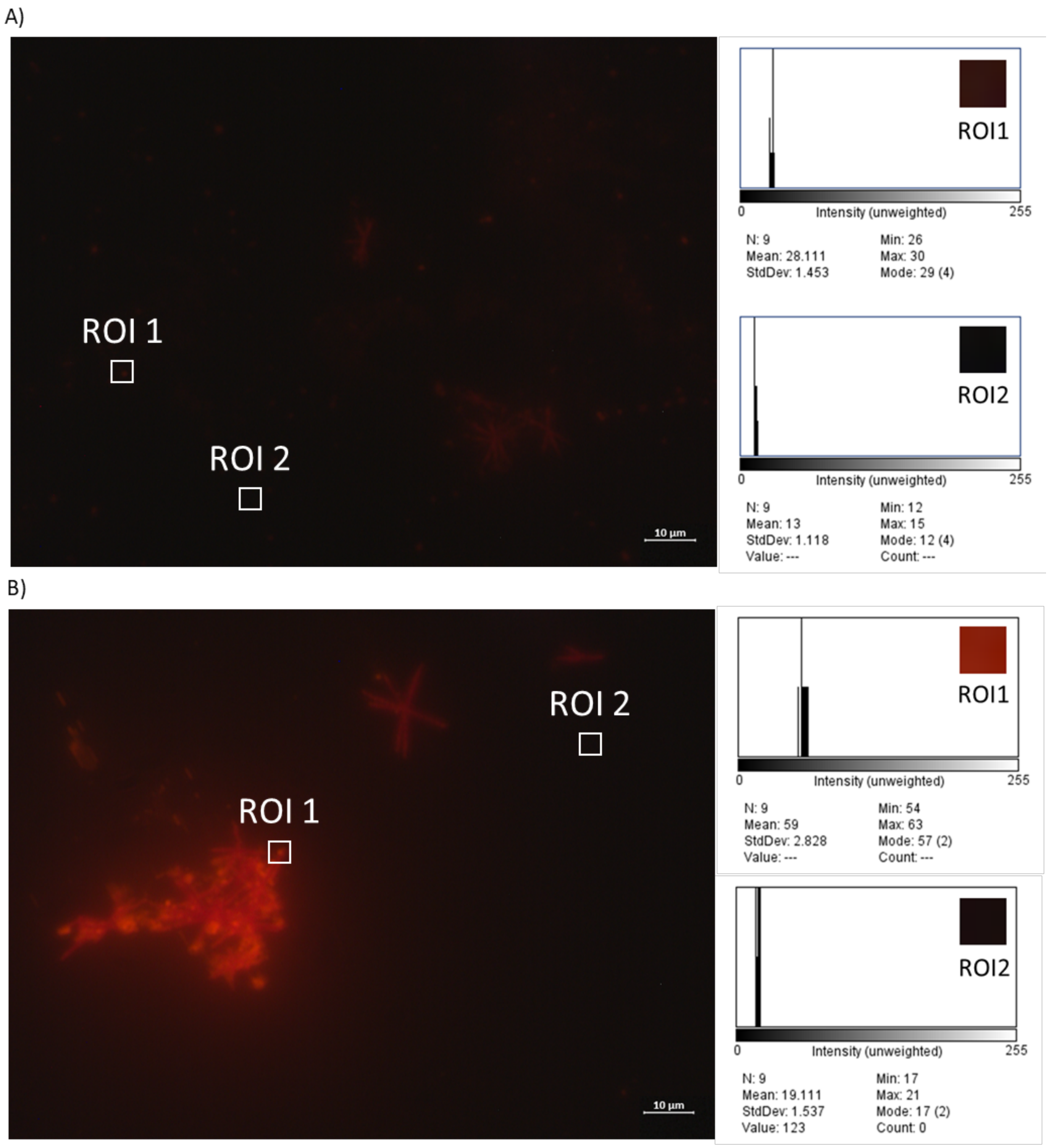

C)

$$EF = \frac{S_M/N_M}{S_G/N_G} \qquad EF = \frac{(59)/(19.111)}{(28.111)/(13)}$$

EF5 for MP in Tap: 1.42

Supplementary Figure 19. Determination of EF5 for MP in tap water on a) glass and b) metasurface using noise (ROI 2, NG and NM) and signals (ROI 1, SG and SM) by c) calculating signal noise ratios.

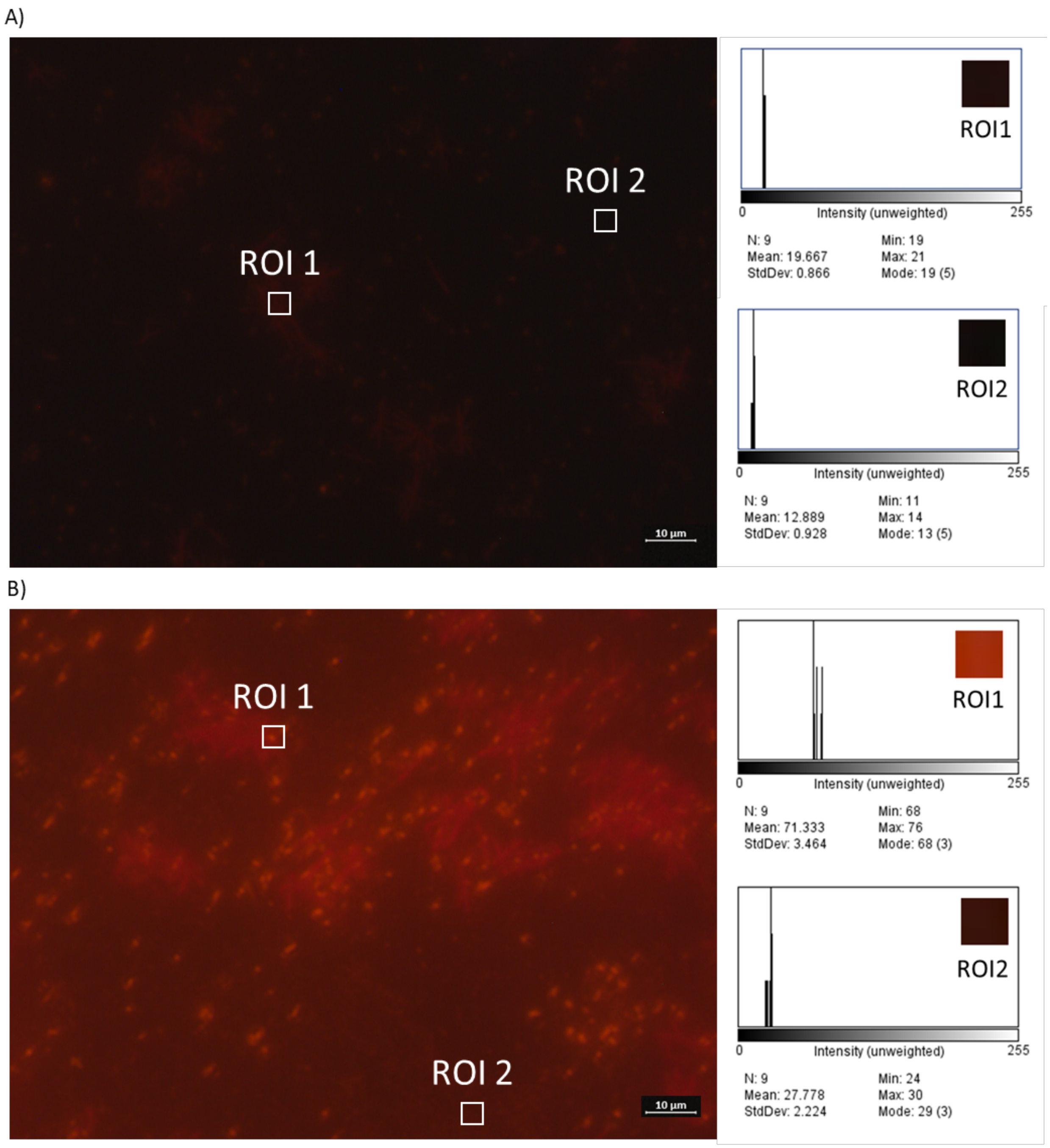

C)

$$EF = \frac{S_M/N_M}{S_G/N_G} \qquad EF = \frac{(71.333)/(27.778)}{(19.667)/(12.889)}$$

EF1 for MP in Lake: 1.68

Supplementary Figure 20. Determination of EF1 for MP in lake on a) glass and b) metasurface using noise (ROI 2, NG and NM) and signals (ROI 1, SG and SM) by c) calculating signal noise ratios.

A)

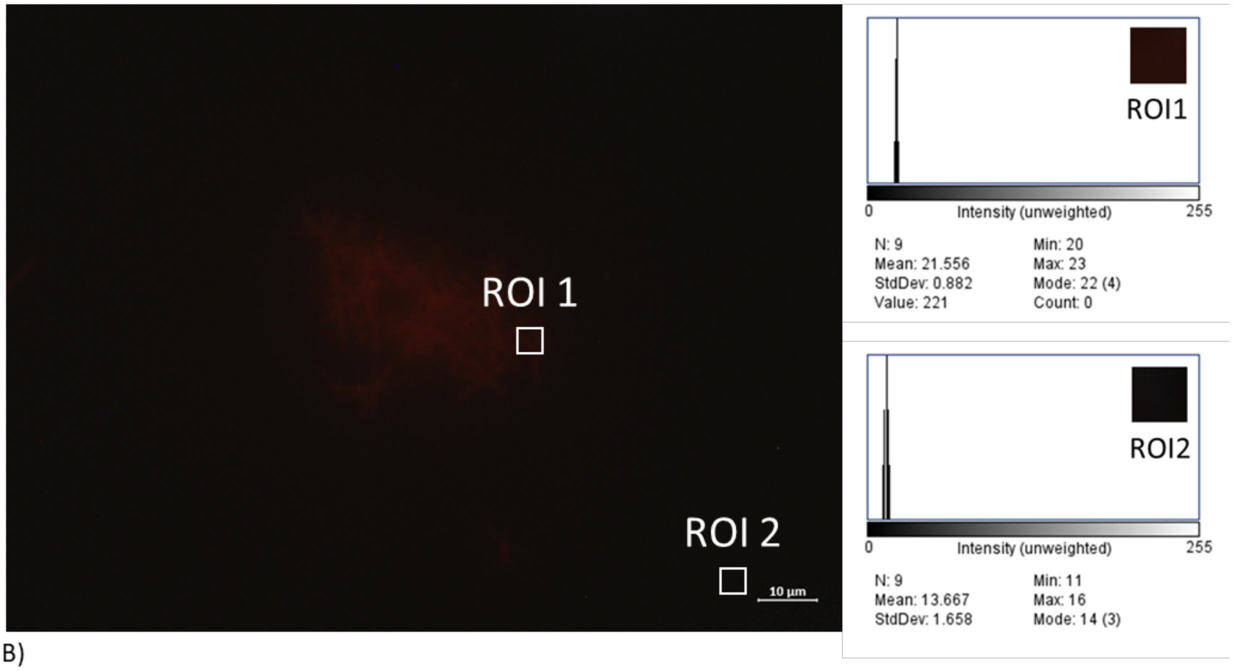

B)

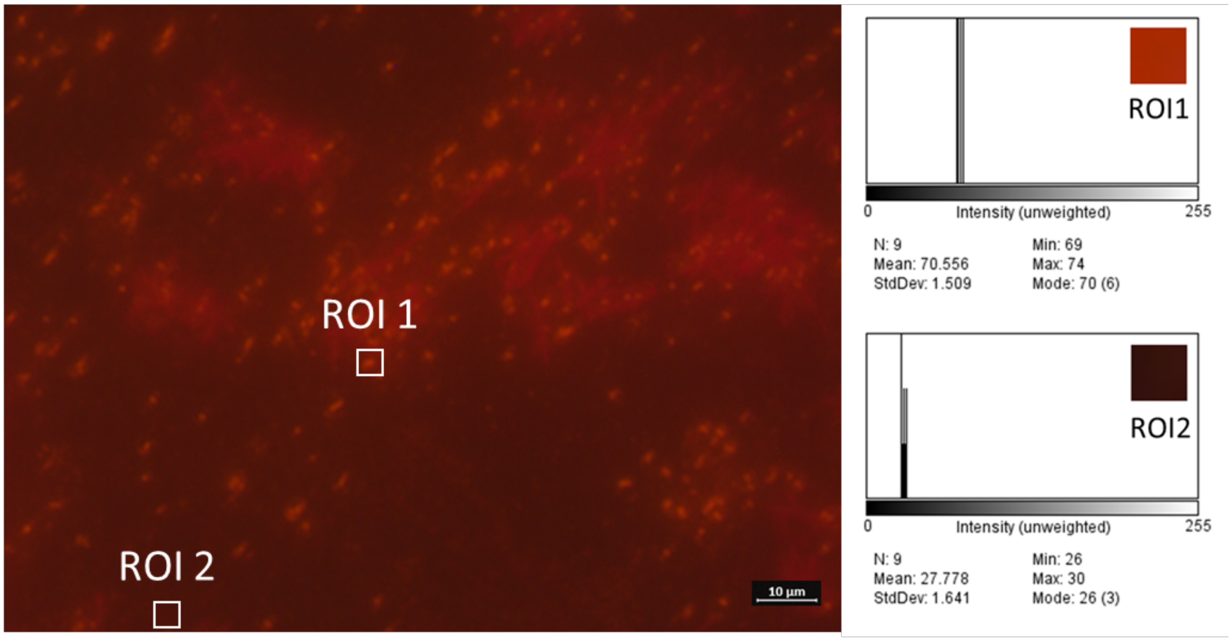

C)

$$EF = \frac{S_M/N_M}{S_G/N_G} \qquad EF = \frac{(70.556)/(27.778)}{(21.556)/(13.667)}$$

EF2 for MP in Lake: 1.61

Supplementary Figure 21. Determination of EF2 for MP in lake on a) glass and b) metasurface using noise (ROI 2, NG and NM) and signals (ROI 1, SG and SM) by c) calculating signal noise ratios.

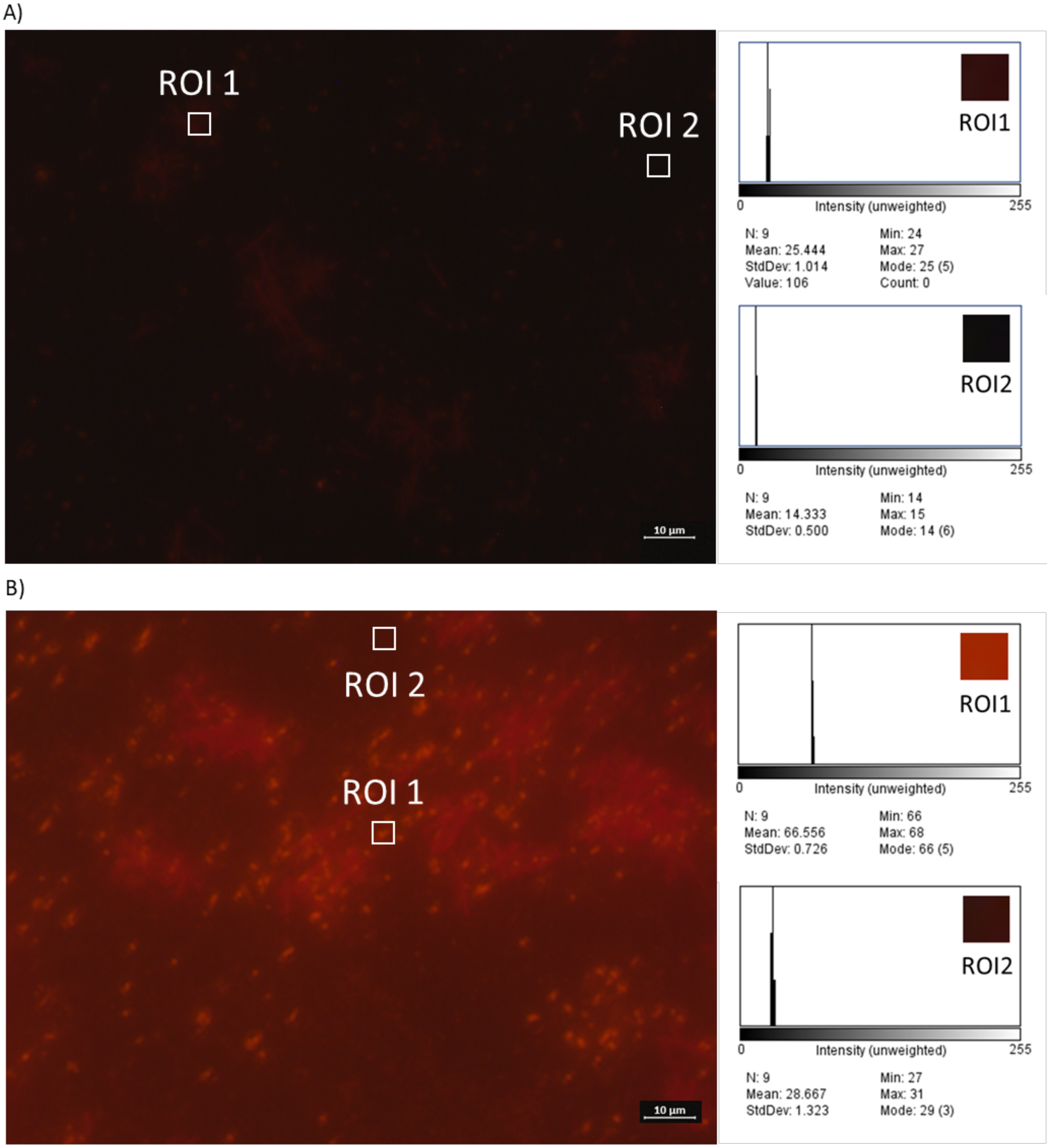

C)

$$EF = \frac{S_M/N_M}{S_G/N_G} \qquad EF = \frac{(66.556)/(28.667)}{(25.444)/(14.333)}$$

EF3 for MP in Lake: 1.30

Supplementary Figure 22. Determination of EF3 for MP in lake on a) glass and b) metasurface using noise (ROI 2, NG and NM) and signals (ROI 1, SG and SM) by c) calculating signal noise ratios.

A)

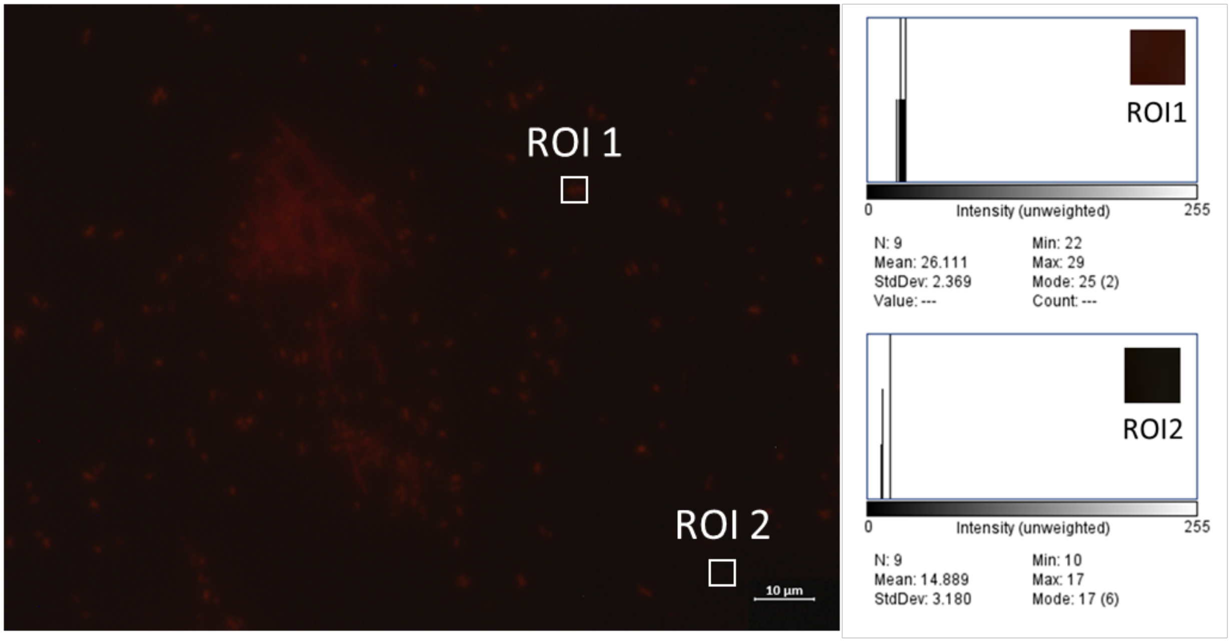

B)

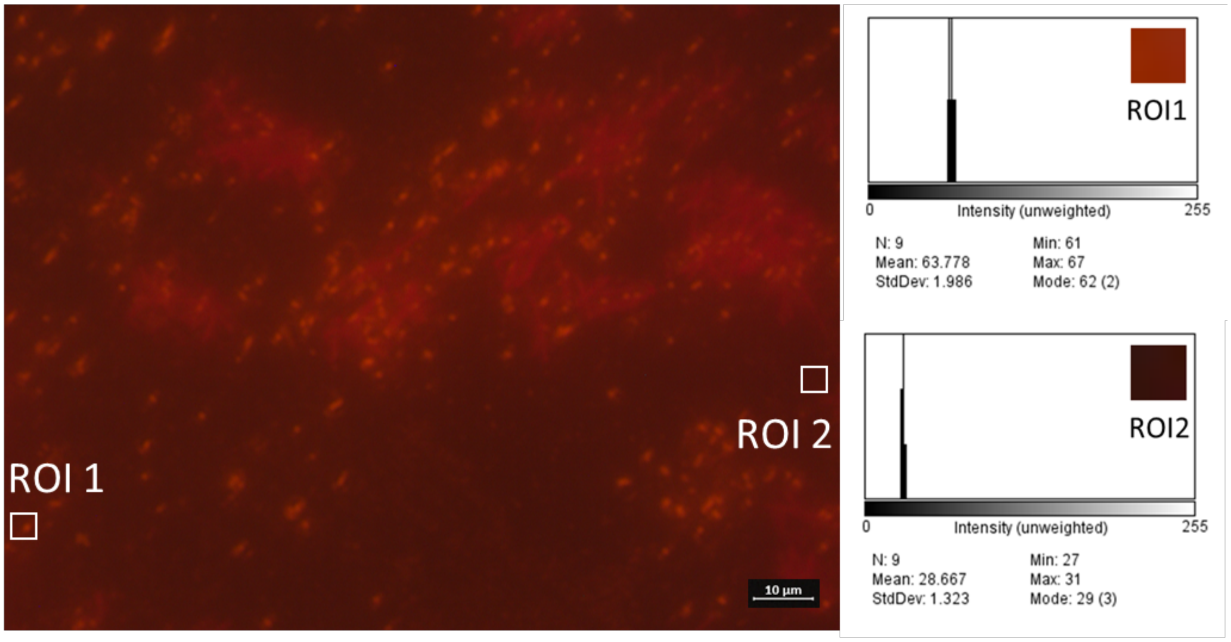

C)

$$EF = \frac{S_M/N_M}{S_G/N_G} \qquad EF = \frac{(63.778)/(28.667)}{(26.111)/(14.889)}$$

EF4 for MP in Lake: 1.27

Supplementary Figure 23. Determination of EF4 for MP in lake on a) glass and b) metasurface using noise (ROI 2, NG and NM) and signals (ROI 1, SG and SM) by c) calculating signal noise ratios.

A)

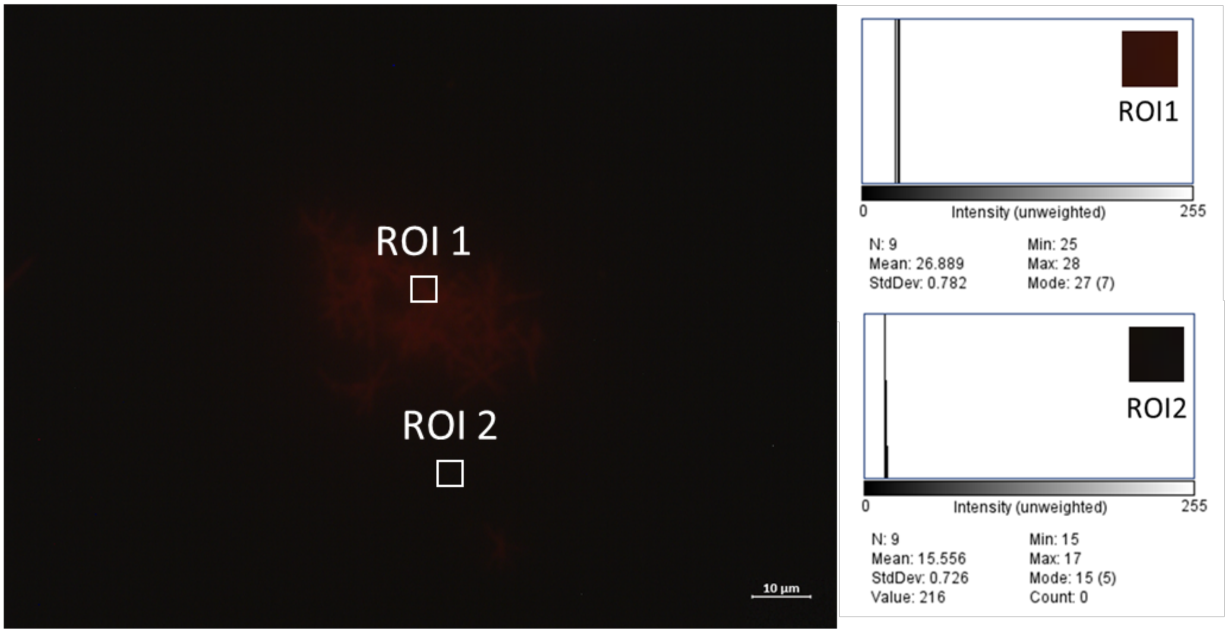

B)

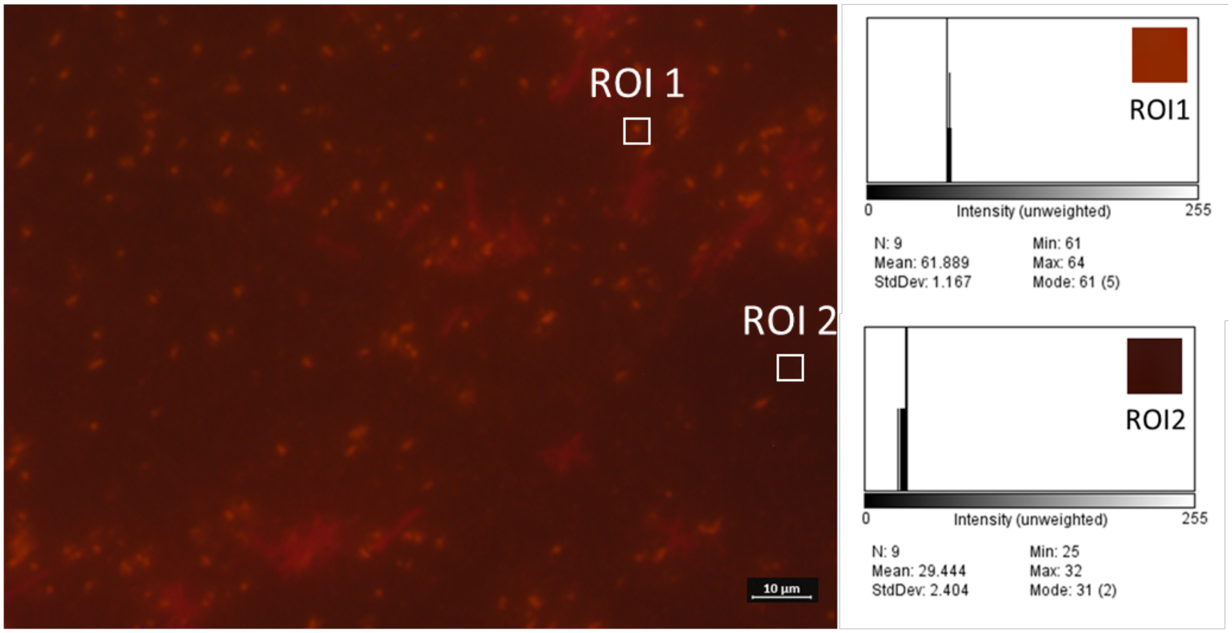

C)

$$EF = \frac{S_M/N_M}{S_G/N_G} \qquad EF = \frac{(61.889)/(29.444)}{(26.889)/(15.556)}$$

EF5 for MP in Lake: 1.21

Supplementary Figure 24. Determination of EF5 for MP in lake on a) glass and b) metasurface using noise (ROI 2, NG and NM) and signals (ROI 1, SG and SM) by c) calculating signal noise ratios.

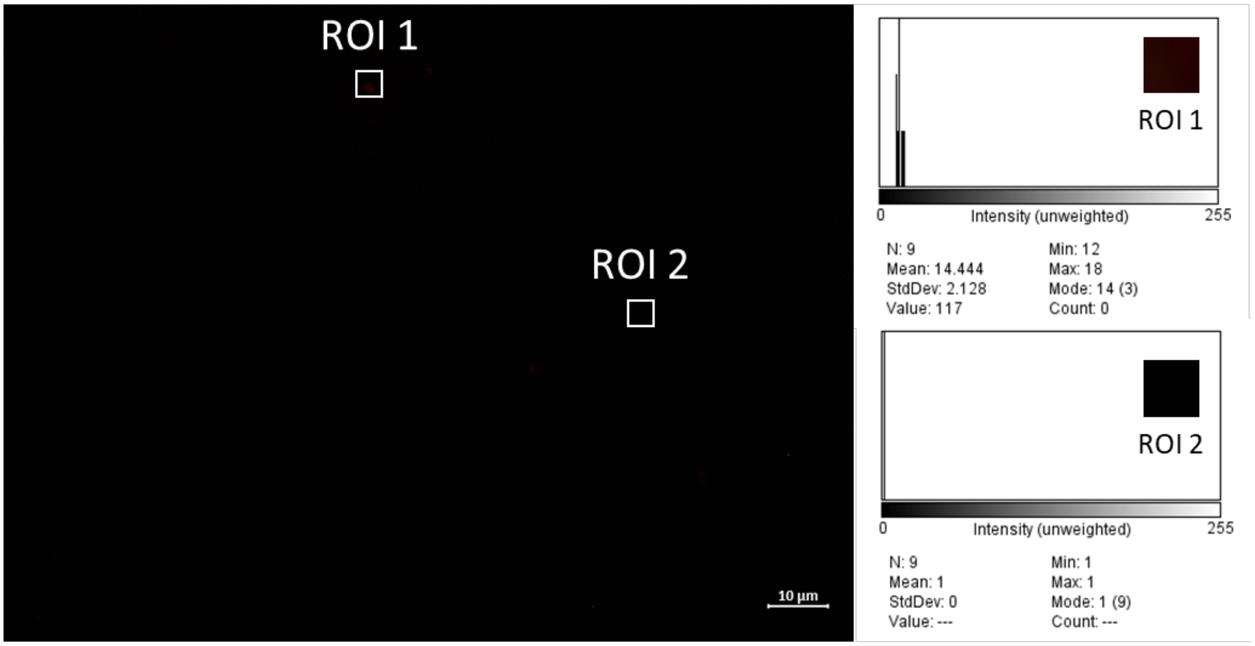

$$EF = \frac{S_M/N_M}{S_G/N_G}$$

$$EF = \frac{(14.444)/(1)}{1}$$

EF1 for ocean MP on metasurface #1: 1.21

Supplementary Figure 25. Determination of EF1 for MP in artificial ocean MP on metasurface #1.

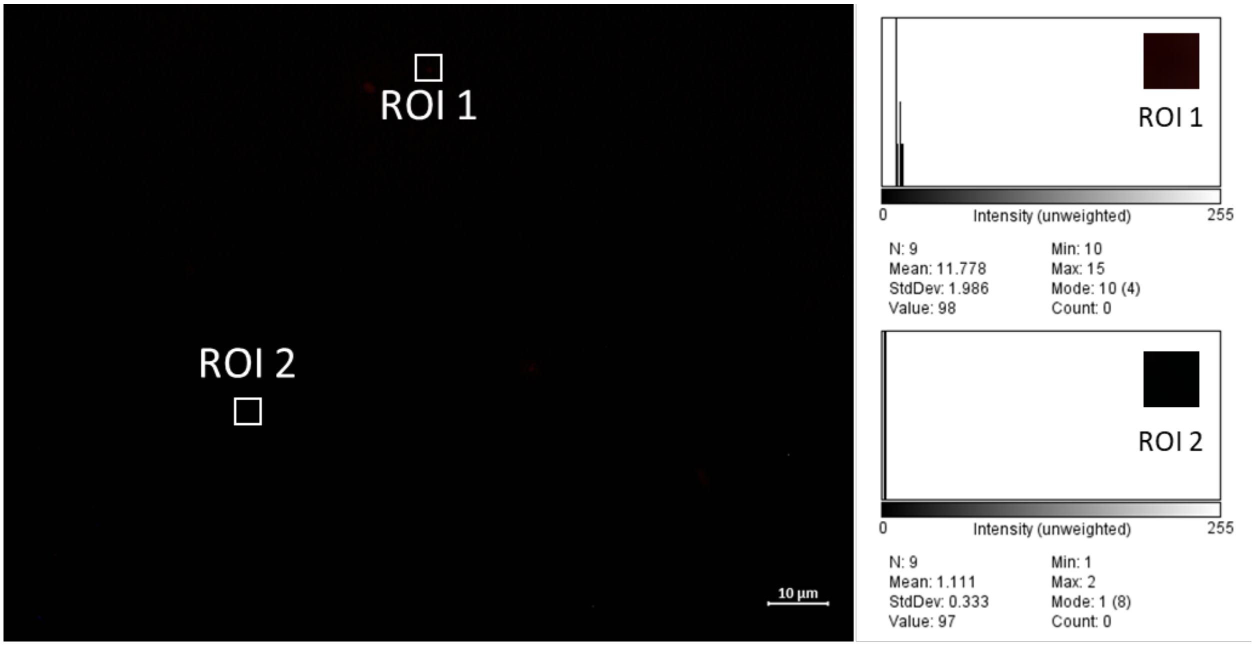

$$EF = \frac{S_M/N_M}{S_G/N_G}$$

$$EF = \frac{(11.778)/(1.111)}{1}$$

EF2 for ocean MP on metasurface #1: 10.601

Supplementary Figure 26. Determination of EF2 for MP in artificial ocean MP on metasurface #1.

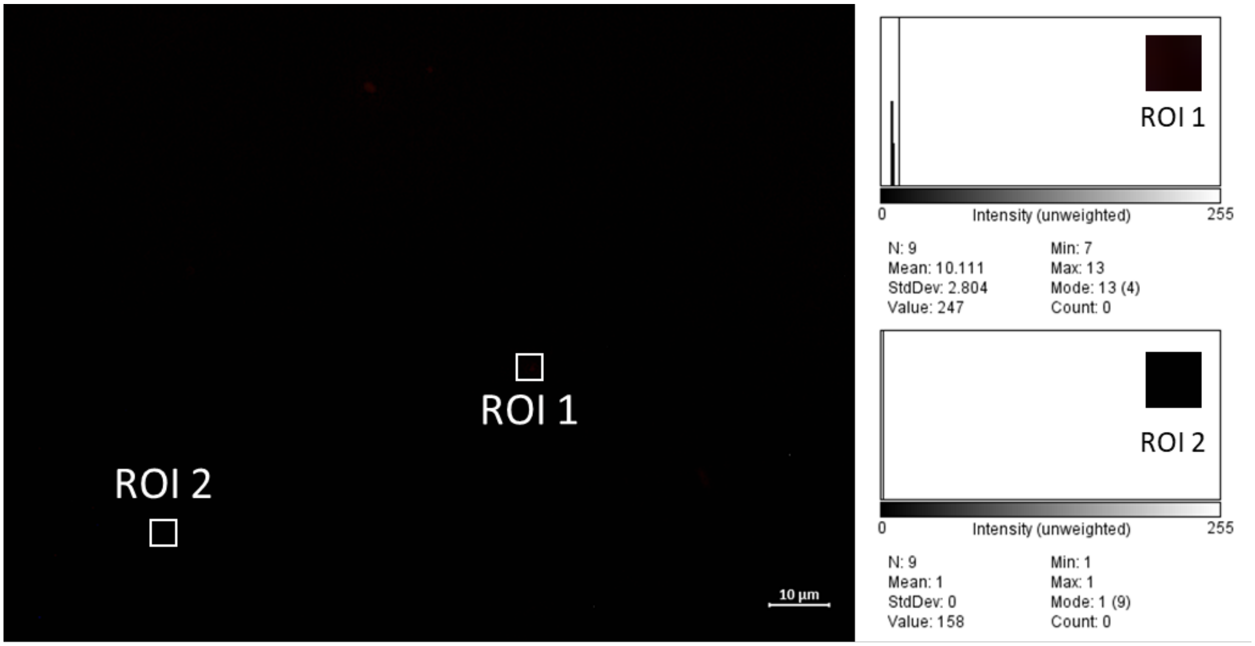

$$EF = \frac{S_M/N_M}{S_G/N_G}$$

$$EF = \frac{(10.111)/(1)}{1}$$

EF3 for ocean MP on metasurface #1: 10.111

Supplementary Figure 27. Determination of EF3 for MP in artificial ocean MP on metasurface #1.

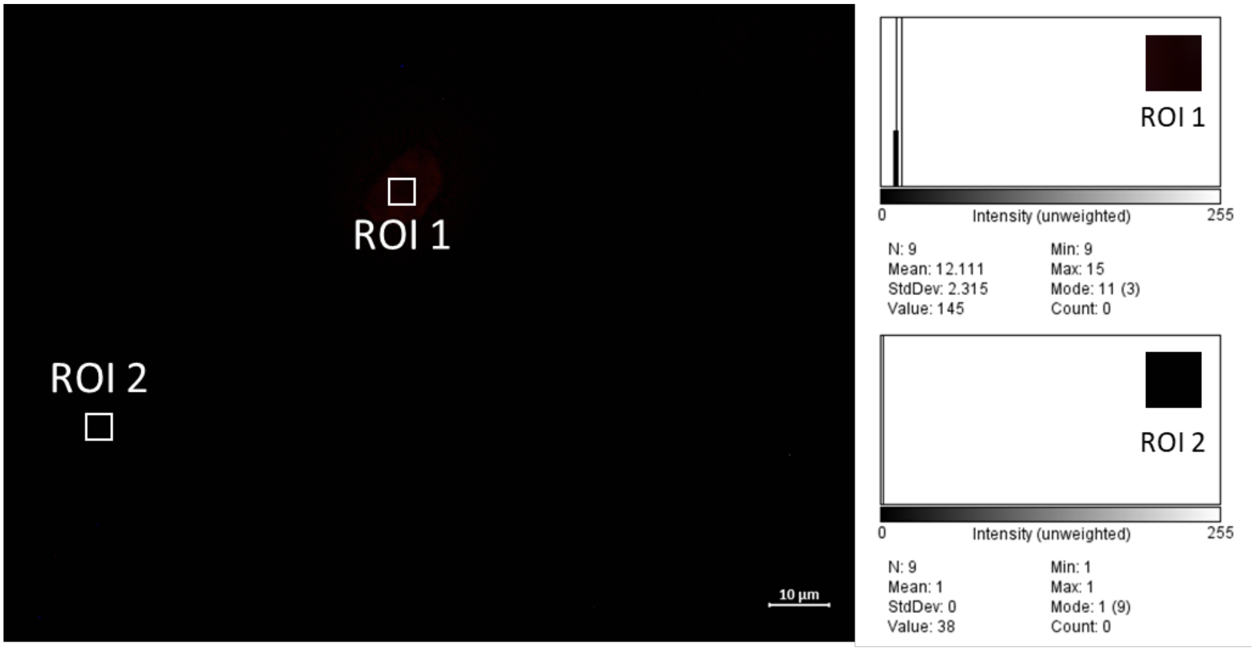

$$EF = \frac{S_M/N_M}{S_G/N_G}$$

$$EF = \frac{(12.111)/(1)}{1}$$

EF4 for ocean MP on metasurface #1: 12.111

Supplementary Figure 28. Determination of EF4 for MP in artificial ocean MP on metasurface #1.

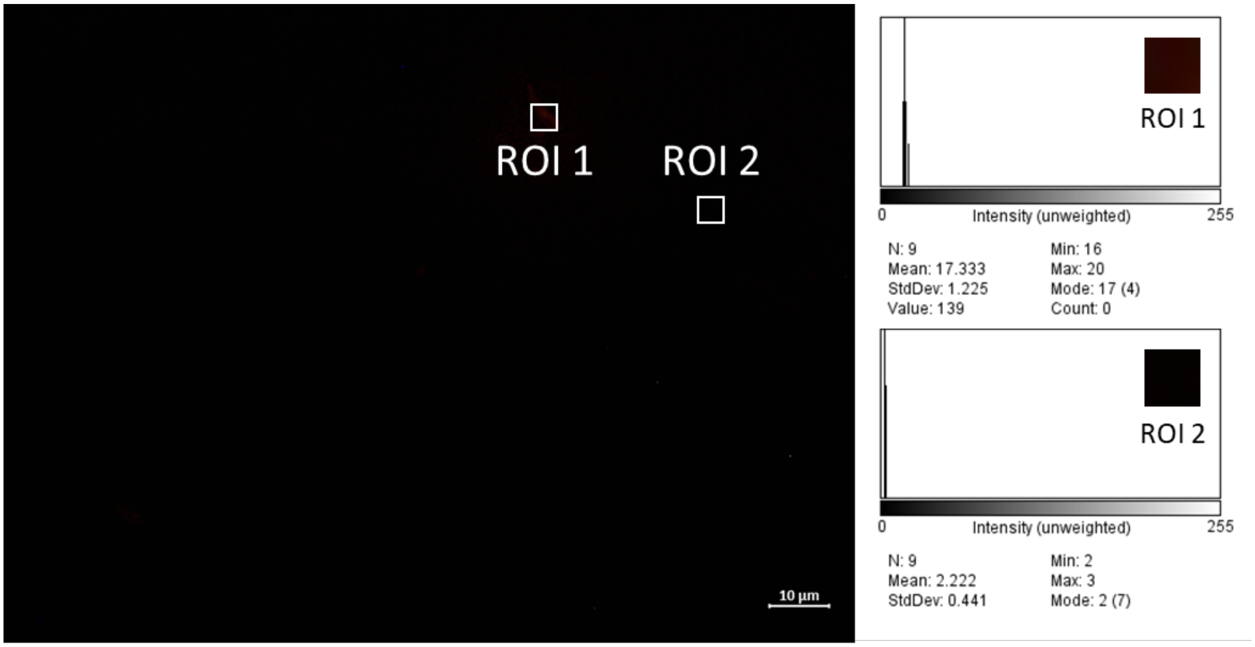

$$EF = \frac{S_M/N_M}{S_G/N_G} \qquad EF = \frac{(17.333)/(2.222)}{1}$$

EF5 for ocean MP on metasurface #1:7.801

Supplementary Figure 29. Determination of EF5 for MP in artificial ocean MP on metasurface #1.

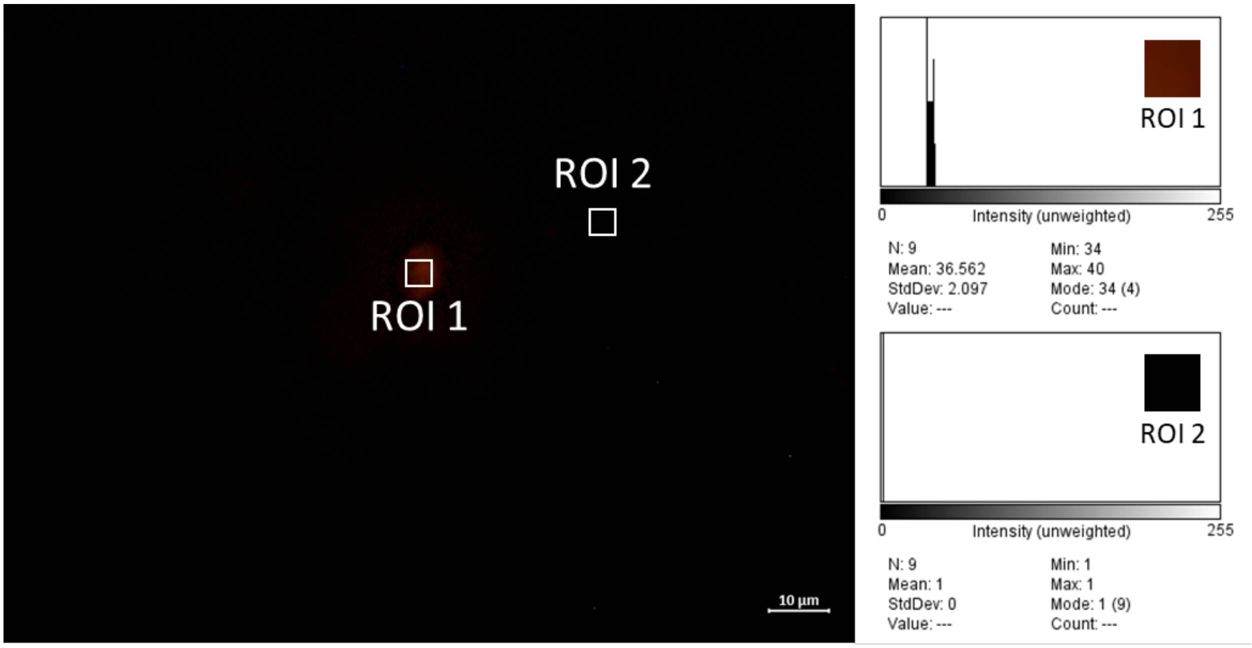

$$EF = \frac{S_M/N_M}{S_G/N_G} \qquad EF = \frac{(36.562)/(1)}{1}$$

EF1 for ocean MP on metasurface #2: 36.562

Supplementary Figure 30. Determination of EF1 for MP in artificial ocean MP on metasurface #2.

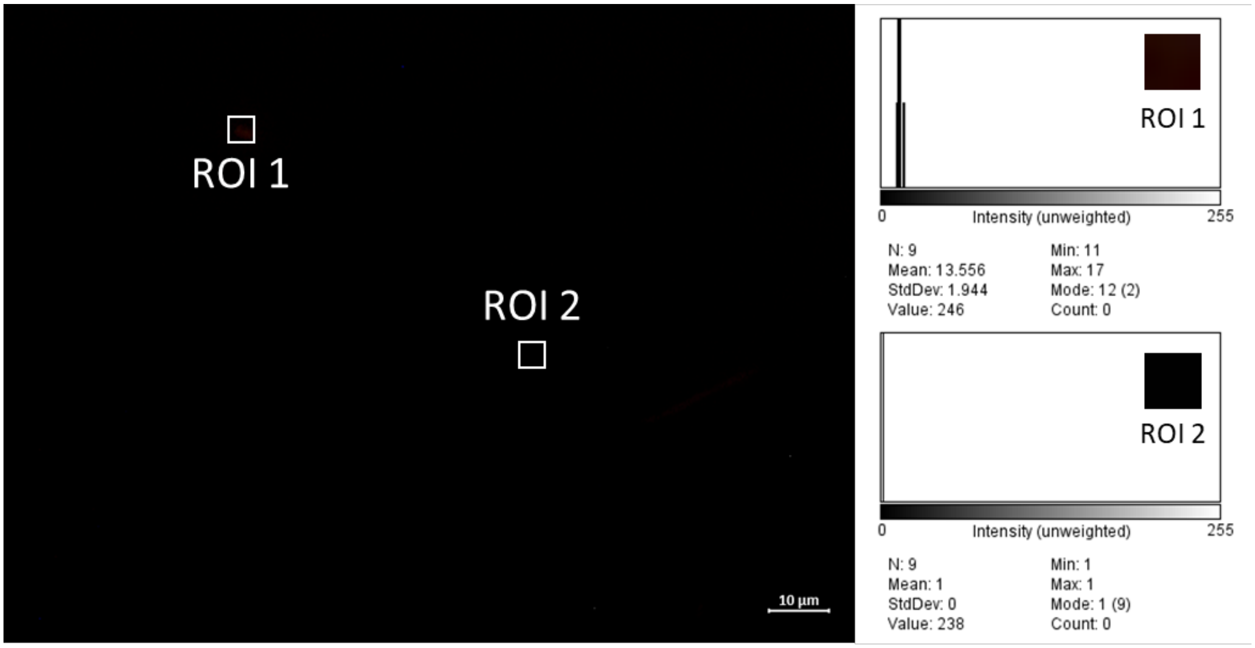

$$EF = \frac{S_M/N_M}{S_G/N_G}$$

$$EF = \frac{(13.556)/(1)}{1}$$

EF2 for ocean MP on metasurface #2: 13.556

Supplementary Figure 31. Determination of EF2 for MP in artificial ocean MP on metasurface #2.

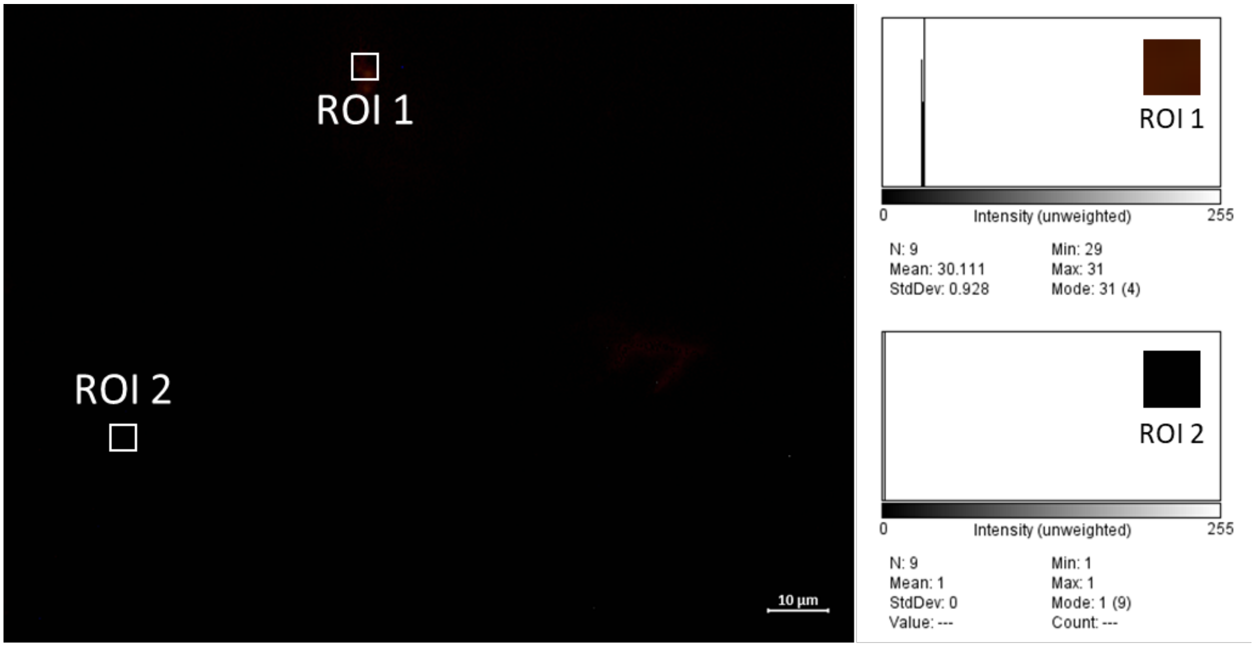

$$EF = \frac{S_M/N_M}{S_G/N_G} \qquad EF = \frac{(30.111)/(1)}{1}$$

EF3 for ocean MP on metasurface #2: 30.111

Supplementary Figure 32. Determination of EF3 for MP in artificial ocean MP on metasurface #2.

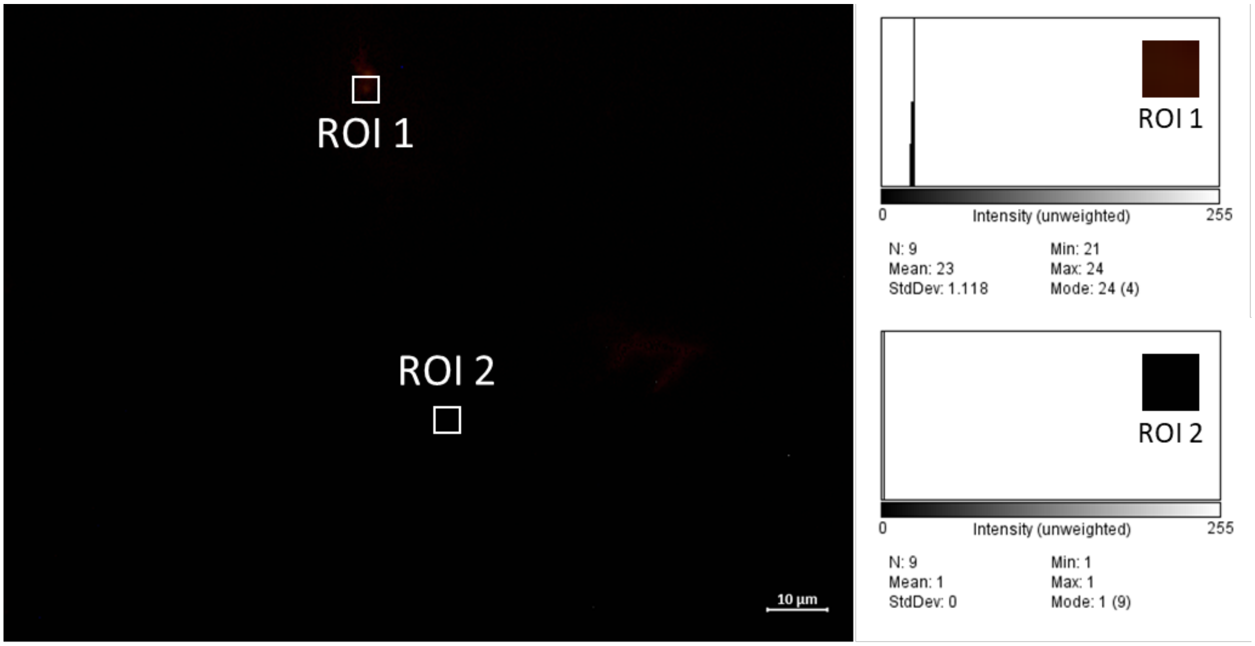

$$EF = \frac{S_M/N_M}{S_G/N_G} \qquad EF = \frac{(23)/(1)}{1}$$

EF4 for ocean MP on metasurface #2: 23

Supplementary Figure 33. Determination of EF4 for MP in artificial ocean MP on metasurface #2.

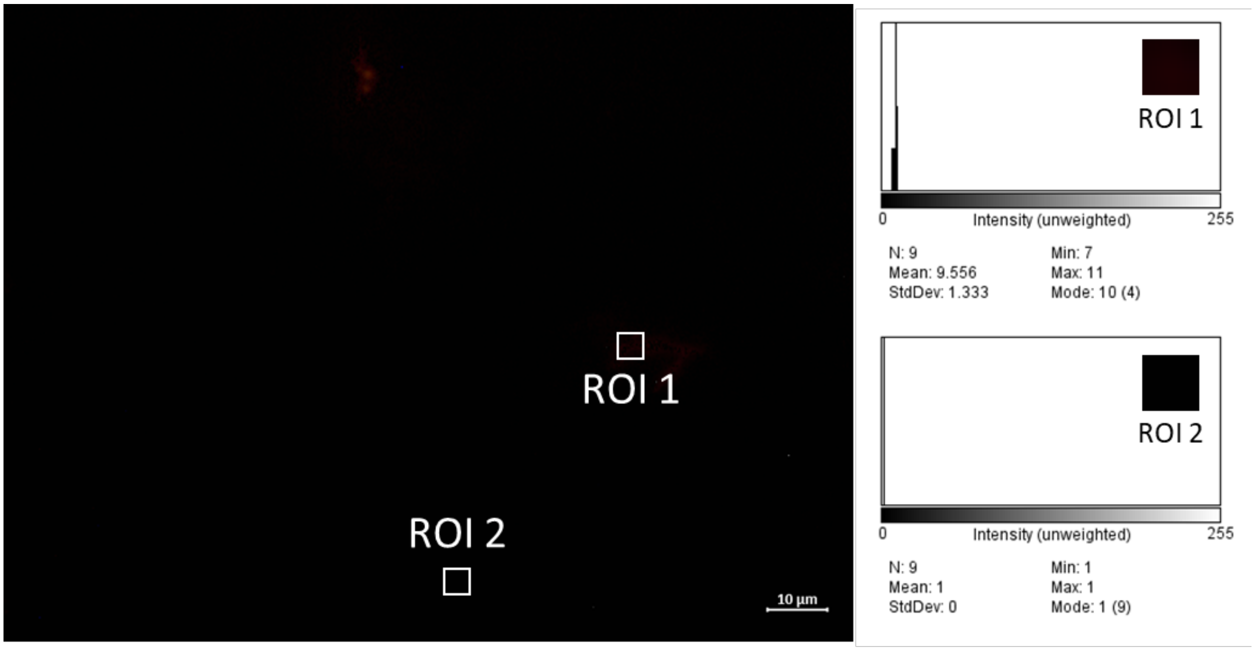

$$EF = \frac{S_M/N_M}{S_G/N_G}$$

$$EF = \frac{(9.556)/(1)}{1}$$

EF5 for ocean MP on metasurface #2: 9.556

Supplementary Figure 34. Determination of EF5 for MP in artificial ocean MP on metasurface #2.

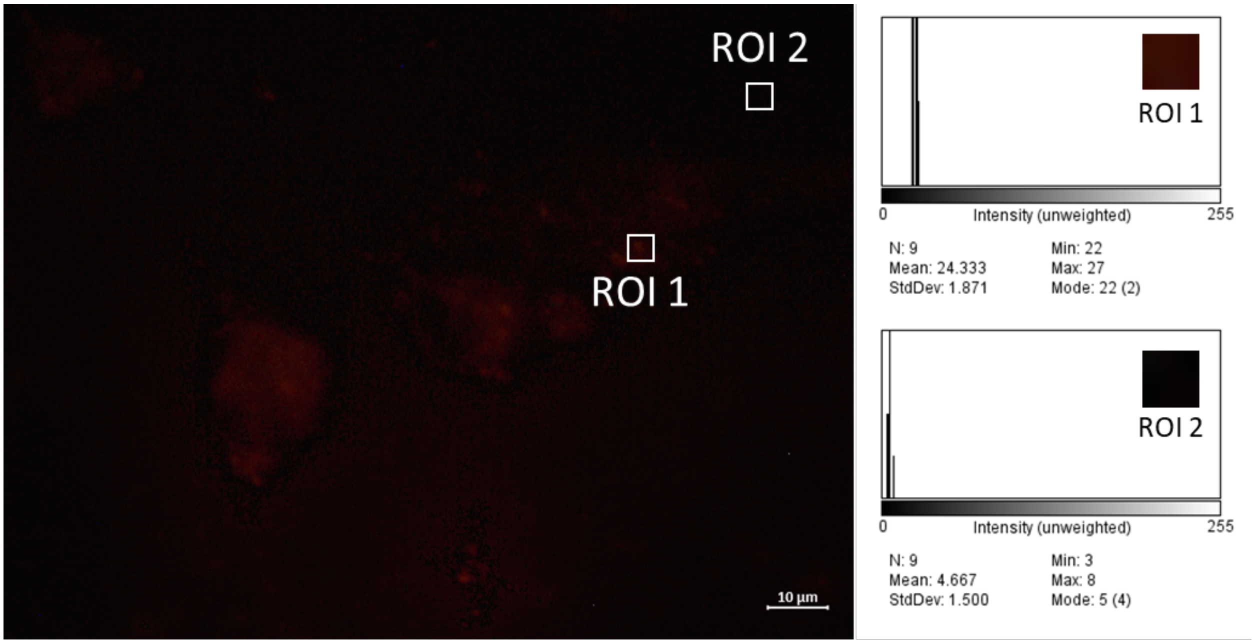

$$EF = \frac{S_M/N_M}{S_G/N_G} \qquad EF = \frac{(24.333)/(4.667)}{1}$$

EF1 for ocean MP on metasurface #3: 5.214

Supplementary Figure 35. Determination of EF1 for MP in artificial ocean MP on metasurface #3.

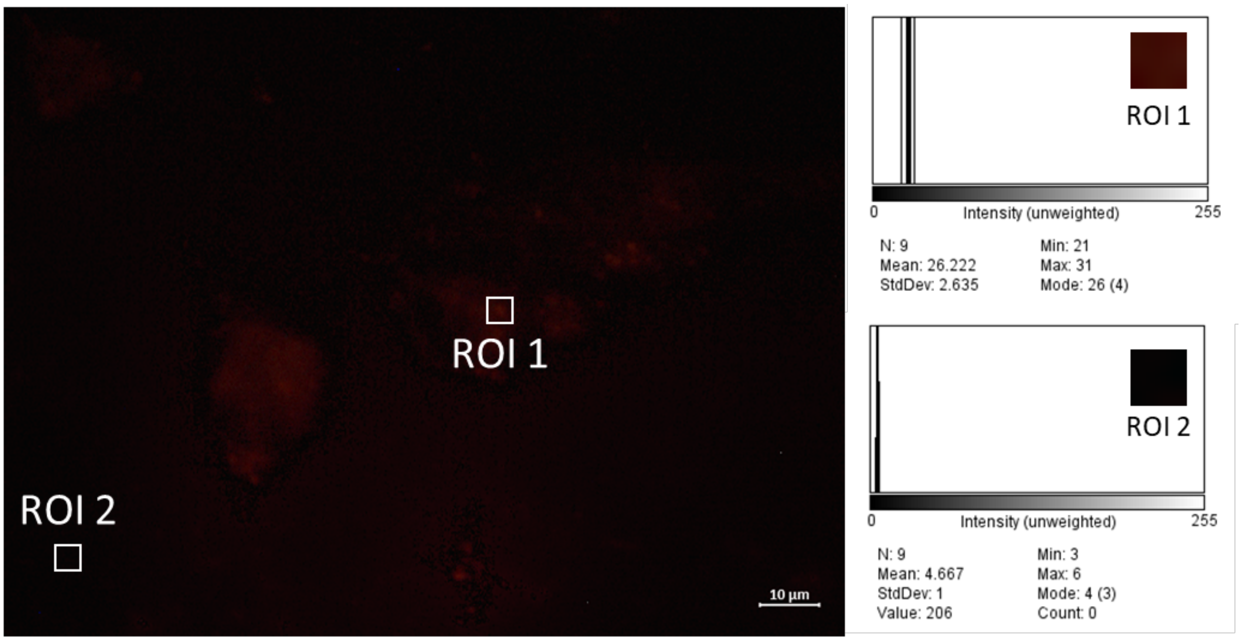

$$EF = \frac{S_M/N_M}{S_G/N_G} \qquad EF = \frac{(26.222)/(4.667)}{1}$$

EF2 for ocean MP on metasurface #3: 5.618

Supplementary Figure 36. Determination of EF2 for MP in artificial ocean MP on metasurface #3.

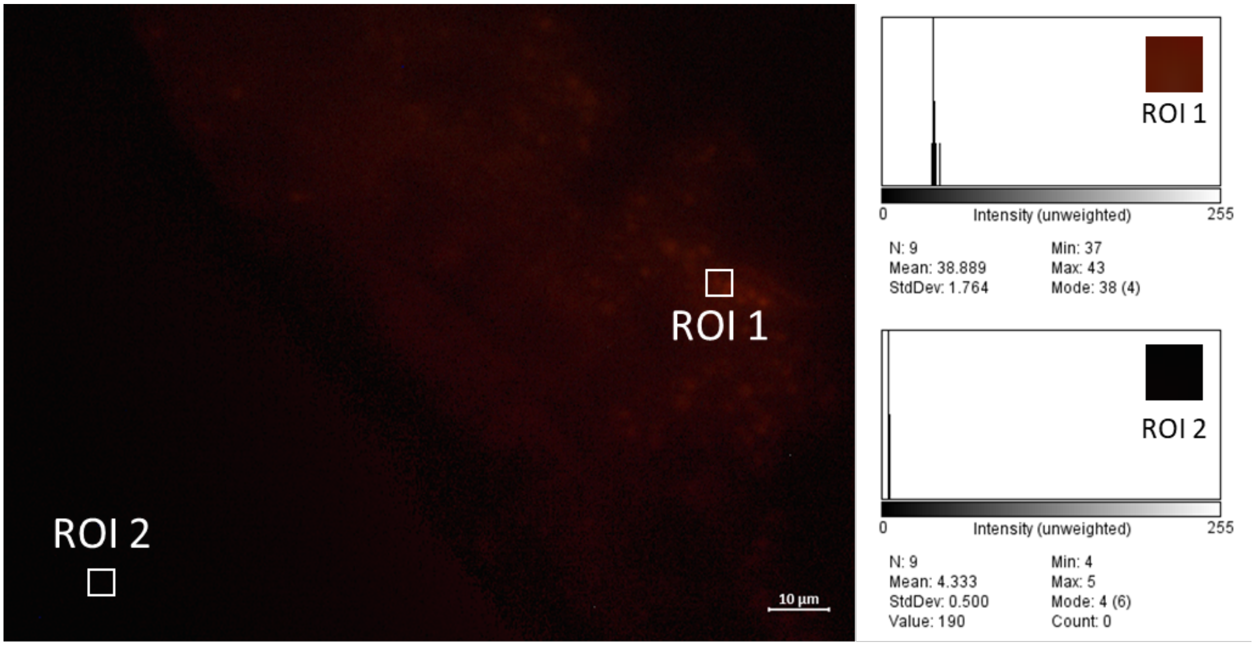

$$EF = \frac{S_M/N_M}{S_G/N_G} \qquad EF = \frac{(38.889)/(4.333)}{1}$$

EF3 for ocean MP on metasurface #3: 8.975

Supplementary Figure 37. Determination of EF3 for MP in artificial ocean MP on metasurface #3.

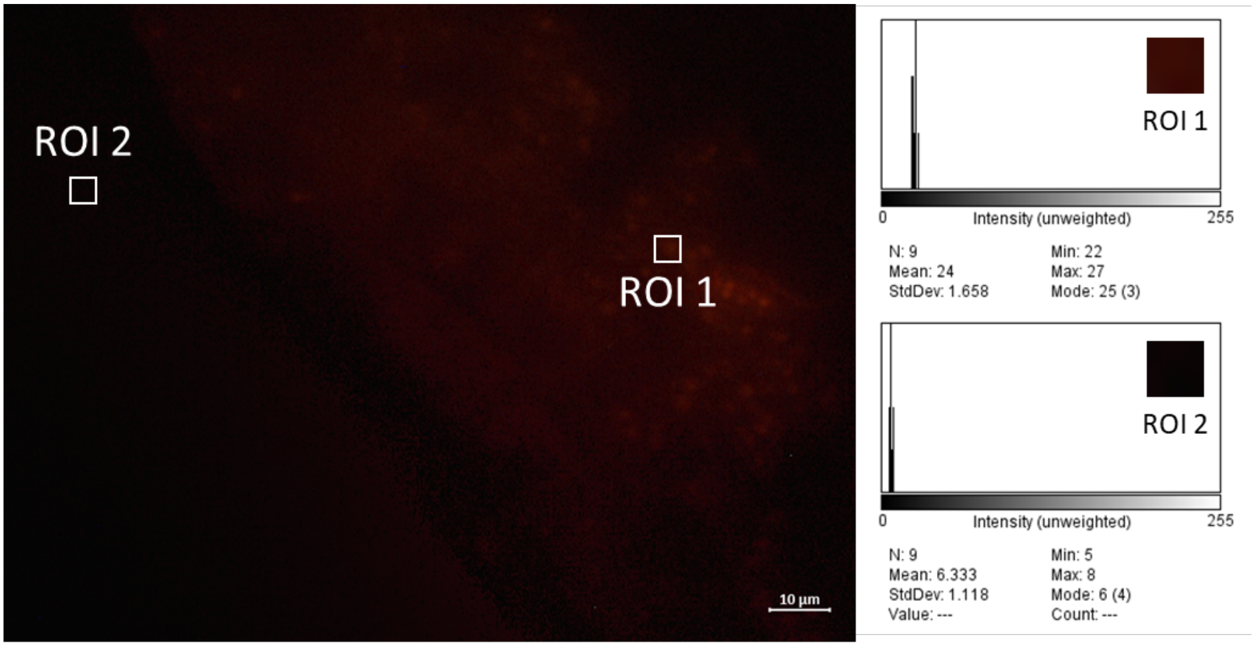

$$EF = \frac{S_M/N_M}{S_G/N_G} \qquad EF = \frac{(24)/(6.333)}{1}$$

EF4 for ocean MP on metasurface #3: 3.789

Supplementary Figure 38. Determination of EF4 for MP in artificial ocean MP on metasurface #3.

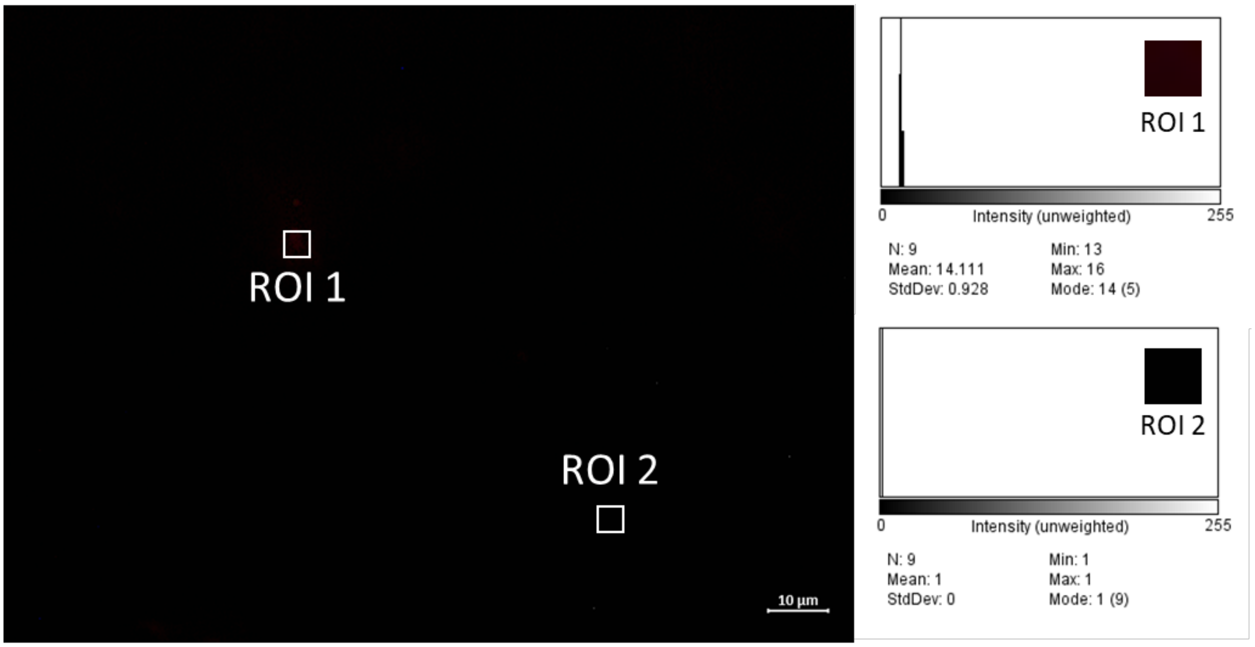

$$EF = \frac{S_M/N_M}{S_G/N_G}$$

$$EF = \frac{(14.111)/(1)}{1}$$

EF5 for ocean MP on metasurface #3: 14.111

Supplementary Figure 39. Determination of EF5 for MP in artificial ocean MP on metasurface #3.

Supplementary Table 1: Comparison of state-of-the-art studies.

Study	Limit of Detection	Size Range	Instrumentation Cost
Maes et al., 2017[1]	20 µm	20-5000 µm	Low
Elsayed et al., 2021[2]	1 µm	1-100 µm	Low
Meyers et al., 2022[3]	20 µm	20-500 µm	Low
Mesquita et al., 2022[4]	10 µm	10-500 µm	Low
Y. Zhang et al., 2022[5]	1 µm	1-100 µm	Low
M. Zhang et al., 2023[6]	10 µm	10-500 µm	Low
Bianco et al., 2023[7]	0.6 µm	0.6-15 µm	Medium
Ko & Chung, 2024[8]	200 nm	0.2-5 µm	Medium
Faramarzi et al., 2024[9]	100 nm	0.1-10 µm	High
Shin et al., 2024[10]	1 µm	1-100 µm	Low
Prasad et al., 2024[11]	1 µm	1-500 µm	Medium
**MicroMetaSense**	250 nm	0.25-100 µm	Low

References

(1) Maes, T.; Jessop, R.; Wellner, N.; Haupt, K.; Mayes, A. G. A Rapid-Screening Approach to Detect and Quantify Microplastics Based on Fluorescent Tagging with Nile Red. *Sci. Rep.* **2017**, *7* (March), 1–10. https://doi.org/10.1038/srep44501.

(2) Elsayed, A. A.; Erfan, M.; Sabry, Y. M.; Dris, R.; Gaspéri, J.; Barbier, J. S.; Marty, F.; Bouanis, F.; Luo, S.; Nguyen, B. T. T.; Liu, A. Q.; Tassin, B.; Bourouina, T. A Microfluidic Chip Enables Fast Analysis of Water Microplastics by Optical Spectroscopy. *Sci. Rep.* **2021**, *11* (1), 1–11. https://doi.org/10.1038/s41598-021-89960-4.

(3) Meyers, N.; Catarino, A. I.; Declercq, A. M.; Brenan, A.; Devriese, L.; Vandegehuchte, M.; De Witte, B.; Janssen, C.; Everaert, G. Microplastic Detection and Identification by Nile Red Staining: Towards a Semi-Automated, Cost- and Time-Effective Technique. *Sci. Total Environ.* **2022**, *823*, 153441. https://doi.org/10.1016/J.SCITOTENV.2022.153441.

(4) Mesquita, P.; Gong, L.; Lin, Y. A Low-Cost Microfluidic Method for Microplastics Identification: Towards Continuous Recognition. *Micromachines* **2022**, *13* (4). https://doi.org/10.3390/mi13040499.

(5) Zhang, Y.; Zhang, M.; Fan, Y. Assessment of Microplastics Using Microfluidic Approach. *Environ. Geochem. Health* **2022**, 1–8. https://doi.org/10.1007/s10653-022-01262-4.

(6) Zhang, M.; Wang, X.; Zhang, Y.; Fan, Y. Integrated Sample Processing and Counting Microfluidic Device for Microplastics Analysis. *Anal. Chim. Acta* **2023**, *1261*, 341237. https://doi.org/10.1016/J.ACA.2023.341237.

(7) Bianco, A.; Carena, L.; Peitsaro, N.; Sordello, F.; Vione, D.; Passananti, M. Rapid Detection of Nanoplastics and Small Microplastics by Nile-Red Staining and Flow Cytometry. *Environ. Chem. Lett.* **2023**, *21* (2), 647–653. https://doi.org/10.1007/S10311-022-01545-3/FIGURES/4.

(8) Ko, K.; Chung, H. Fluorescence Microfluidic System for Real-Time Monitoring of PS and PVC Sub-Micron Microplastics under Flowing Conditions. *Sci. Total Environ.* **2024**, *950*, 175016. https://doi.org/10.1016/J.SCITOTENV.2024.175016.

(9) Faramarzi, P.; Jang, W.; Oh, D.; Kim, B.; Kim, J. H.; You, J. B. Microfluidic Detection and

Analysis of Microplastics Using Surface Nanodroplets. *ACS Sensors* **2024**, *9* (3), 1489–1498. https://doi.org/10.1021/acssensors.3c02627.

(10) Shin, S.; Jeon, B.; Kang, W.; Kim, C.; Choi, J.; Hong, S. C.; Lee, H. H. Characterization of Microfluidic Trap and Mixer Module for Rapid Fluorescent Tagging of Microplastics. *Microfluid. Nanofluidics* **2024**, *28* (4), 1–12. https://doi.org/10.1007/S10404-024-02716-0/FIGURES/5.

(11) Prasad, S.; Bennett, A.; Triantafyllou, M. Characterization of Nile Red-Stained Microplastics through Fluorescence Spectroscopy. *J. Mar. Sci. Eng. 2024, Vol. 12, Page 1403* **2024**, *12* (8), 1403. https://doi.org/10.3390/JMSE12081403.
